# A Genetic Screen and Transcript Profiling Reveal a Shared Regulatory Program for *Drosophila* Linker Histone H1 and Chromatin Remodeler CHD1

**DOI:** 10.1534/g3.115.016709

**Published:** 2015-01-27

**Authors:** Harsh Kavi, Xingwu Lu, Na Xu, Boris A. Bartholdy, Elena Vershilova, Arthur I. Skoultchi, Dmitry V. Fyodorov

**Affiliations:** Department of Cell Biology, Albert Einstein College of Medicine, Bronx, New York 10461

**Keywords:** *Drosophila melanogaster*, *His1*, *Chd1*, chromatin, gene regulation

## Abstract

Chromatin structure and activity can be modified through ATP-dependent repositioning of nucleosomes and posttranslational modifications of core histone tails within nucleosome core particles and by deposition of linker histones into the oligonucleosome fiber. The linker histone H1 is essential in metazoans. It has a profound effect on organization of chromatin into higher-order structures and on recruitment of histone-modifying enzymes to chromatin. Here, we describe a genetic screen for modifiers of the lethal phenotype caused by depletion of H1 in *Drosophila melanogaster*. We identify 41 mis-expression alleles that enhance and 20 that suppress the effect of *His1* depletion *in vivo*. Most of them are important for chromosome organization, transcriptional regulation, and cell signaling. Specifically, the reduced viability of H1-depleted animals is strongly suppressed by ubiquitous mis-expression of the ATP-dependent chromatin remodeling enzyme CHD1. Comparison of transcript profiles in H1-depleted and *Chd1* null mutant larvae revealed that H1 and CHD1 have common transcriptional regulatory programs *in vivo*. H1 and CHD1 share roles in repression of numerous developmentally regulated and extracellular stimulus-responsive transcripts, including immunity-related and stress response-related genes. Thus, linker histone H1 participates in various regulatory programs in chromatin to alter gene expression.

The genomes of all eukaryotes are packaged into a compact nucleoprotein complex called chromatin (Van Holde 1989; Wolffe 1998). The histones are a family of basic proteins that play key roles in organizing chromatin structure. There are five major classes of histones, the core histones H2A, H2B, H3, and H4, and the linker histone usually referred to as H1. The nucleosome core particle is the highly conserved repeating unit of chromatin. It is formed through association of an octamer of the four core histones with ∼145 bp of DNA. The linker histone H1 binds to the nucleosome core particle near the site at which DNA enters and exits the core particle. Binding of H1 results in protection of an additional ∼20 bp of the linker DNA between core particles. H1 binding also stabilizes the association between DNA and core particles and facilitates folding of oligonucleosome arrays into higher-order structures (Ramakrishnan 1997; Wolffe 1997; Lever *et al.* 2000).

Chromatin is a dynamic structure that undergoes a variety of modifications, including methylation of DNA, posttranslational modifications of histones, and local changes in compaction. H1 binding to chromatin is highly dynamic with an average residence time of minutes (Misteli *et al.* 2000; Bustin *et al.* 2005). Therefore, H1 is expected to play a key role in regulating these changes in chromatin organization. Until recently, it was thought H1 functions primarily as a chromatin architectural protein. However, there are now an increasing number of reports of physical interactions between H1 and a variety of chromatin modifiers and transcriptional regulators, including enzymes that methylate DNA and histones (Lu *et al.* 2013; Yang *et al.* 2013), transcription factors (Lee *et al.* 2004; Kim *et al.* 2008), nucleolar proteins (Kalashnikova *et al.* 2013), and other factors (McBryant *et al.* 2010). These findings suggest that there are multiple cellular pathways that intersect with H1. To begin to identify these pathways and their components, we undertook a genetic screen for modifiers of the lethal phenotype caused by depletion of H1 in *Drosophila melanogaster*.

Unbiased genetic screens for dominant enhancers and suppressors of phenotypes associated with a loss of function of a target gene are widely used to identify novel pathways of cellular metabolism and unknown factors that contribute to well-characterized biological processes (Spector and Osley 1993; Breslow *et al.* 2008; Fillingham *et al.* 2008). Unicellular model eukaryotic organisms, although better adapted for genetic studies, are not really suitable for analyzing interactions with H1 because they do not express canonical linker histones or their H1 homologs are not essential. Therefore, *Drosophila* offers one of the best opportunities for investigating genetic interactions with H1 *in vivo*. The genetic screen described here relies on UAS-controlled mis-expression of target genes in the collection of *EP* insertion lines (Rørth 1996). The modular UAS-GAL4 system makes use of two types of transgenes. One of them directs tissue-specific expression of yeast GAL4 transactivator, which drives conditional expression of endogenous fly genes via binding to multiple UAS sites of the partner *P{EP}* transgenes randomly inserted throughout the genome. This system has been used previously to identify the network of factors that functionally interact with *brm* (Armstrong *et al.* 2005), *dom* (Kwon *et al.* 2013), and a dominant-negative allele of *Iswi* (Burgio *et al.* 2008).

Unlike classical “synthetic lethal or sick” interaction screens in yeast, our strategy allows us to analyze interactions of *Drosophila* mis-expression alleles with a “hypomorphic” allele of *His1*, which affords several advantages. First, this type of screen provides an opportunity to identify both enhancers and suppressors of *His1*. Second, it produces a quantitative measure of interactions. And, finally, it has the potential to uncover not only factors that have a competitive/cooperative function with H1 but also genes that are epistatic with *His1* (*e.g.*, regulators of H1 expression and deposition into chromatin). Unexpectedly, in the genetic screen described here, we identified *Chd1*, which encodes a SNF2-related ATPase, as an enhancer of *His1*. Furthermore, by analyzing transcript profiles in H1-depleted and *Chd1* mutant larvae, we found that these two factors share a genome-wide role in repression of numerous gene targets *in vivo* in *Drosophila*. These results suggest a mechanism for cooperative regulation of genetic activity by chromatin structural proteins (linker histone H1) and remodeling enzymes (CHD1).

## Materials and Methods

### Fly strains and genetics

Flies were grown on standard corn meal, sugar, and yeast medium with Tegosept. Stocks and crosses were maintained in circulating air or water thermostats at 18–29°. The null allele of *Chd1* (*Chd1^1^*) as well as *pINT-H1^4M^* and *pINT-H1^1M^ P*-element insertion alleles (second and third chromosomes, respectively) that encompass a *UAS*-driven *His1* hairpin transgene and produce moderate H1 knockdown under the control of *Tubulin*- or *Act5C-GAL4* drivers were described elsewhere (Konev *et al.* 2007; Lu *et al.* 2009). The *P{Act5C-GAL4}25FO1* driver, balancer lines and the collection of *EP* mis-expression insertions (Rørth 1996) were obtained from the Bloomington Stock Center.

The recombinant *Act5C-GAL4*, *pINT-H1^4M^* second chromosome was constructed in a series of crosses and balanced. For initial viability tests, 10 virgin *Act5C-GAL4*, *pINT-H1^4M^/SM5* or *Act5C-GAL4/SM5* females were mated with 10 *w^1118^* males and reared at 20, 23, 26, or 29°. After 3 d of egg deposition, the parents were transferred to a fresh vial, allowed to deposit eggs for an additional 3 d, and discarded. The adult F1 progeny carrying either the balancer (*Cy*) or the GAL4 driver-carrying chromosome (*Cy^+^*) were scored. The offspring were scored twice in each of the two vials at 2 d and 5 d after the beginning of eclosion, and the four numbers were combined.

*Chd1^1^/CyO*; *Tub-GAL4/TM3*, *Sb* and *Chd1^1^/CyO*; *pINT-H1^1M^* fly lines were constructed in a series of crosses. To examine potential genetic interactions of *Chd1* and *His1*, virgin *Chd1^1^/CyO*; *Tub-GAL4/TM3*, *Sb* females were mated with *Chd1^1^/CyO*; *pINT-H1^1M^* males and reared at 18, 22, 26, or 29°. The adult progeny carrying the TM3 balancer chromosome (*Sb*) were discarded, and the remaining flies were scored as either *Chd1* homozygotes (*Cy^+^*) or heterozygotes (*Cy*). An *inter se* cross of *Chd1^1^/CyO* flies was performed as a control, and *Chd1* homozygotes and heterozygotes were scored as above.

### Genetic screen

**Figure 1:**
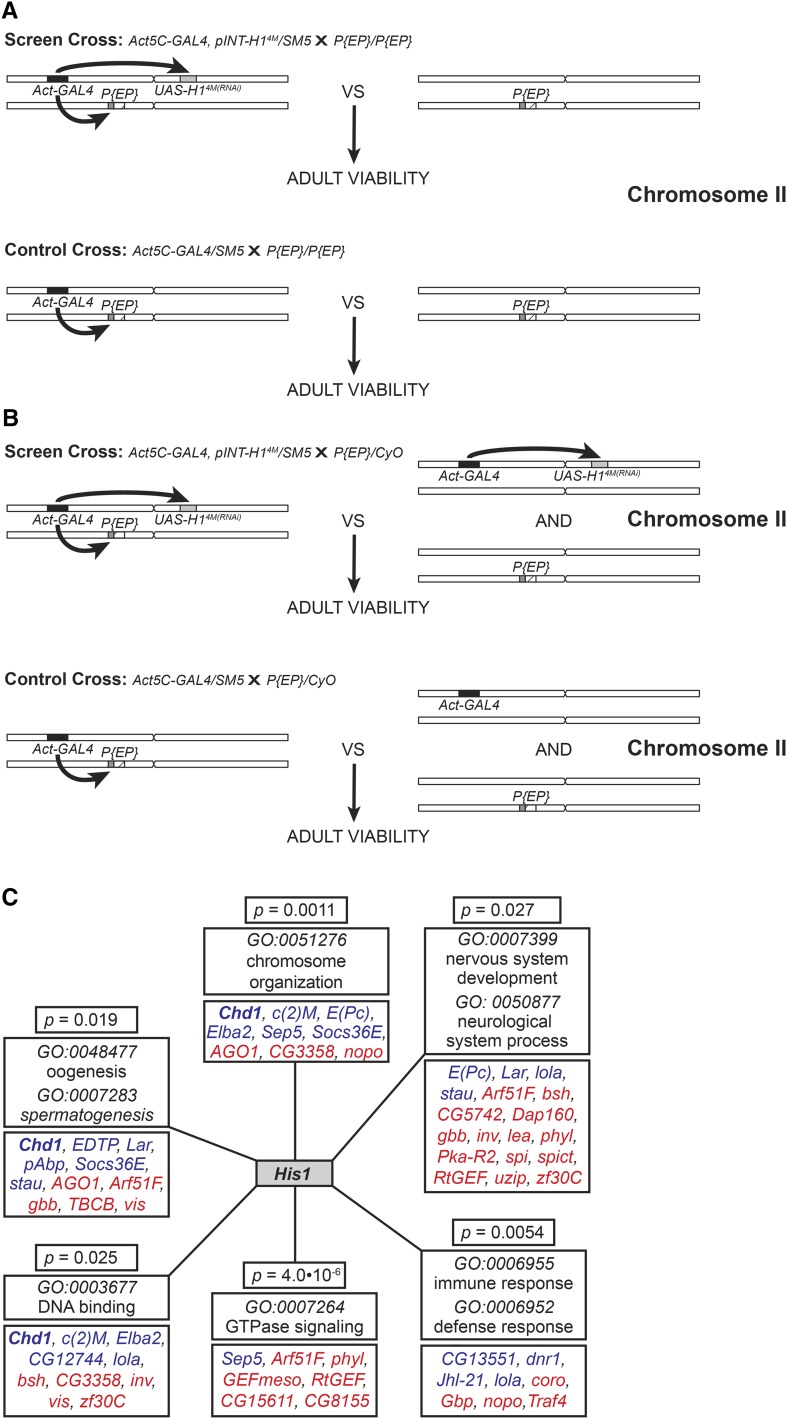
Genetic screen for modifiers of *His1*-dependent adult viability using mis-expression alleles on the second chromosome of *Drosophila melanogaster*. (A) A genetic cross for screening homozygous viable and fertile *EP* insertions on the second chromosome. Heterozygous *Act5C-GAL4*, *pINT-H1^4M^/SM5* females were mated to *P{EP}/P{EP}* males, and the ratios of H1-depleted and normal adult progeny were scored based on the balancer phenotypic marker (*Cy*). Positions of *Act5C-GAL4*, *pINT-H1^4M^* and *P{EP}* insertions are indicated by black, gray, and white boxes, respectively. The endogenous gene affected by the UAS promoter in the *P{EP}* insertion is indicated by a hatched box. (B) A genetic cross for screening homozygous lethal and male sterile *EP* insertions on the second chromosome. Heterozygous *Act5C-GAL4*, *pINT-H1^4M^/SM5* females were mated to *P{EP}/SM5* or *P{EP}/CyO* males, and the ratios of H1-depleted and normal adult progeny were scored based on the balancer phenotypic marker (*Cy*). (C) Over-represented GO terms and their members among alleles genetically interacting with *His1*. Gene symbols representing enhancers and suppressors are indicated by red and blue type, respectively. *P* values are calculated by the hypergeometric test.

Ten virgin *Act5C-GAL4*, *pINT-H1^4M^/SM5* or *Act5C-GAL4/SM5* females were mated with 10 males carrying an *EP* insertion on the second chromosome (534 alleles), either homozygous or balanced heterozygous (Figure 1, A and B), and reared at 27°. Otherwise, the crosses were performed, and the adult progeny were scored exactly as described above. At least 50 adult F1 flies were scored for each cross.

### Statistical analyses of genetics interactions

All statistical tests were performed separately for data sets of viability numbers from crosses with homozygous and heterozygous *EP* fathers. Probability values and odds ratios (OR) were calculated using the two-tailed Fisher’s exact and binomial tests to reject the null hypothesis (variation of viability through random, independent actions of H1 knockdown and gene mis-expression in the *EP* alleles). *P*<0.01 was considered statistically significant.

To identify enriched GO term (biological function) classes that are significantly enriched (*P*<0.05) among genetic interactors, the NIH web-based tool DAVID (Huang da *et al.* 2009a,b) was used. *P* values for enrichment of individual and combined GO terms were re-calculated by the hypergeometric test.

### Microarray data analyses

The RNA expression profiling data were processed and analyzed using the GeneSpring GX11 software (Agilent Technologies) as described (Lu *et al.* 2013). Briefly, the raw data (.cel) files were normalized at the probe level by Robust Multichip Average algorithm (RMA) (Irizarry *et al.* 2003), and the significant differential abundance between RNAi and corresponding control conditions was performed using *t*-test statistics (*P*<0.05, alpha level) and varying fold-change thresholding (ranging from 25% to 100% of reproducible change, based on the target modulation stringency). The functional annotations of the resulting gene lists were performed using DAVID.

For analyses of a correlation between transcriptional repression targets of various chromatin effectors, Gene Expression Omnibus (GEO) database entries were analyzed by the GEO web-based tool, tabulated in Excel, cross-referenced according to the Affymetrix probe IDs or Flybase IDs and sorted by the relative fold-change and statistical significance. *P* values were calculated by the hypergeometric test.

### Quantitative real-time RT-PCR

H1 expression was reduced to ∼5% of wild-type level in *pINT-H1^5M^/Tub-GAL4* L3 larvae at 29°. Animals that underwent reduction of expression of an unrelated gene *Nau* were used as a control (Lu *et al.* 2009). Total RNA from 10 to 20 pairs of salivary glands from larvae of each genotype was isolated by Trizol extraction (Invitrogen) and quantitated with a NanoDrop 1000 Spectrophotometer (Thermo Scientific); 1 µg total RNA was treated with RNAse-free DNase I (Promega) and random-primed cDNA was prepared using the SuperScript II kit (Invitrogen). Real-time quantitative PCR amplification reactions were performed in an ABI Prism 7700 sequence detection system (Applied Biosystems). One-step RT-PCR was performed using SYBR Green Quantitative RT-PCR kit as per the manufacturer’s instructions. Primer sequences are available on request. To quantitate the expression levels, CT values of an endogenous reference gene, *rp49*, were included. All reactions were performed in triplicate, along with no-template controls.

## Results and Discussion

### Genetic screen for enhancers and suppressors of H1 RNAi-induced lethality

The conventional strategy for genetic interaction screens in *Drosophila* using a collection of UAS mis-expression insertion alleles (Rørth 1996) often utilizes tissue-specific GAL4 drivers and relies on modification of visible phenotypes in adult eye, wing, leg, or notum (Arancio *et al.* 2010; Kwon *et al.* 2013). Positive and negative genetic interactions are typically scored as modifications of the phenotype strength and/or penetrance. Unfortunately, the depletion of H1 by RNAi in a tissue-specific manner fails to consistently produce such visible phenotypes (data not shown). However, viability of adult flies that carry a UAS-driven H1 RNAi transgene and a ubiquitous GAL4 driver varies over a wide range (from apparently normal viability to complete lethality) depending on the temperature and inversely correlates with the extent of H1 depletion (Lu *et al.* 2009). Therefore, we decided to base our genetic screen on increased or decreased adult viability under conditions of moderate H1 depletion and mis-expression of the second chromosome genes using the *EP* collection of UAS-controlled mis-expression alleles.

First, to streamline the screen, we prepared a recombinant *Act5C-GAL4*, *pINT-H1^4M^/SM5* fly line. This driver-transgene combination allele is homozygous lethal; however, the balanced heterozygous allele can be maintained at 18° without an apparent effect on the endogenous H1 protein level or adult viability. Because we wanted to screen for both potential enhancers and suppressors of the H1 RNAi-induced phenotype, we tested the effect on viability in a range of temperatures (Table 1). To this end, we crossed heterozygous *w^1118^*; *Act5C-GAL4*, *pINT-H1^4M^/SM5* females to *w^1118^* males and scored the ratios of H1-depleted and normal progeny based on the balancer phenotypic marker (*Cy*). As a control, similar crosses were performed with *w^1118^*; *Act5C-GAL4/SM5* females. At 26°, H1 depletion resulted in a substantial decrease in the proportion of *Cy^+^* progeny (to ∼52% of the expected Mendelian ratio indicating approximately three-fold decrease in viability, relative to the unaffected siblings). In contrast, ubiquitous GAL4 expression alone did not result in a significant decrease of viability at any temperature (Table 1). Therefore, we decided to perform the screen at 27°. We anticipated that the partial decrease of viability achieved at 27°, although statistically significant in comparison with the control, would provide sufficient dynamic range to allow detection of both enhancement and suppression to achieve statistical significance (*P*<0.01) while scoring a relatively small number of adult progeny (50–100).

**Table 1:** Temperature-dependent viability of *Act5C-GAL4*, *pINT-H1^4M^/+* adult flies

*w^1118^*	Temperature			
Cross With	20°	23°	26°	29°
*Act5C-GAL4*, *pINT-H1^4M^/SM5*	91/178 (102%)	68/152 (89%)	40/155 (52%)	5/232 (4%)
*Act5C-GAL4/SM5* (control)	77/148 (104%)	104/203 (102%)	91/193 (94%)	80/176 (91%)
*P*	0.87	0.23	4.4⋅10^−5^	1.5⋅10^−26^

Heterozygous *Act5C-GAL4*, *pINT-H1^4M^/SM5*, *Cy* and *Act5C-GAL4/SM5*, *Cy* females were mated with *w^1118^* males. The crosses were set at indicated temperatures. Viability was scored as the number of eclosed *Cy^+^* adults relative to the total number of offspring. Percent viability compared with the expected number of *Cy^+^* flies (calculated from the Mendelian distribution) is shown in parentheses. Probability values are calculated by the chi-square two-way test.

For the actual genetic screen, all crosses were performed exactly as the control experiments (Table 1), except males of the *EP* insertion alleles on the second chromosome were substituted for the *w^1118^* males; 433 of the *EP* insertions are homozygous viable, and males with a homozygous insertion were used for the crosses (Figure 1A). For lethal and male sterile insertions (110), we crossed heterozygous males (balanced with *CyO* or *SM5*) and adjusted the scoring accordingly (Figure 1B). In the screen and control crosses, the GAL4 driver targets ubiquitous mis-expression of both the tested genes and H1-specific dsRNA to all tissues of the developing progeny.

Mis-expression of individual genes alone had variable effects on viability of adult *Act5C-GAL4/P{EP}* offspring, ranging from complete lethality to a significant increase (Table 2 and data not shown). For instance, whereas *P{EP}Traf4^EP578^* insertion is homozygous viable, ubiquitous mis-expression of Traf4 in *Act5C-GAL4/P{EP}Traf4^EP578^* adults causes lethality (0% of *Cy^+^* adults are recovered in the offspring). In contrast, mis-expression of MESK2 in *Act5C-GAL4/P{EP}MESK2^EP2347^* flies increases the proportion of *Cy^+^* adults in the progeny of the cross to ∼66% or ∼132% relative to the expected Mendelian ratio (Table 2). The distribution of viability effects was close to normal (binomial), with a mean fraction of *Act5C-GAL4/P{EP}* adults of ∼47% (∼94% of expected) in crosses with homozygous fathers or ∼33% (99% of expected) in crosses with heterozygous fathers.

**Table 2:** Second chromosome *EP* alleles that genetically interact with *His1*

*EP* Allele	*EP* Males	H1KD + *EP*: *Cy*	H1KD + *EP*: *Cy^+^*	*EP*: *Cy*	*EP*: *Cy^+^*	NE	*P* (FET)	*P* (bin)	Gene Symbol	Flybase ID
*P{EP}Dap160^EP2543^*	hom	135	0	70	4	**0.00**	6.1E-08	4.3E-10	*Dap160*	FBgn0023388
*P{EP}G3075*	hom	92	0	58	5	**0.00**	1.2E-07	3.0E-13	*bsh*	FBgn0000529
*P{EP}G18270*	hom	135	0	97	6	**0.00**	6.1E-06	4.3E-08	*phyl*	FBgn0013725
*P{EP}gbb^G12649^*	het	66	0	31	24	**0.00**	1.2E-07	4.1E-11	*gbb*	FBgn0024234
*P{EP}G2573*	hom	67	0	87	3	**0.00**	2.6E-06	2.8E-11	*CG10631*	FBgn0032817
*P{EP}G3720*	hom	63	0	114	36	**0.00**	6.4E-06	1.3E-10	*CG15611*	FBgn0034194
*P{EP}slim^G8310^*	hom	81	0	56	8	**0.00**	5.8E-07	1.7E-09	*slim*	FBgn0261477
*P{EP}mrj^EU639^*	hom	111	0	107	2	**0.00**	2.8E-03	8.6E-03	*mrj*	FBgn0034091
*P{EP}pain^EP2251^*	hom	113	0	81	2	**0.00**	1.3E-04	2.2E-04	*pain*	FBgn0060296
*P{EP}RpS15^G3660^*	het	73	1	141	55	**0.11**	1.9E-06	1.0E-09	*RpS15*	FBgn0034138
*P{EP}CG5846^G2283^*	hom	63	2	71	76	**0.16**	2.5E-05	3.8E-08	*CG5846*	FBgn0032171
*P{EP}vis^G2720^*	hom	65	3	39	39	**0.23**	7.8E-04	1.0E-07	*vis*	FBgn0033748
*P{EP}MESK2^EP2347^*	hom	62	4	18	35	**0.24**	4.1E-03	1.5E-06	*MESK2*	FBgn0043070
*P{EP}CG5742^G8286^*	hom	97	3	68	31	**0.25**	2.0E-05	3.5E-12	*CG5742*	FBgn0034304
*P{EP}G2957*	hom	82	4	56	42	**0.28**	7.1E-03	4.2E-05	*hbs*	FBgn0029082
*P{EP}Nedd8^G7338^*	hom	87	5	99	97	**0.29**	2.6E-07	3.3E-13	*Nedd8*	FBgn0032725
*P{EP}TBCB^G2655^*	het	82	4	35	19	**0.30**	3.5E-03	1.0E-03	*TBCB*	FBgn0034451
*P{EP}CG13751^G18007^*	hom	72	4	82	62	**0.32**	1.1E-03	6.6E-08	*CG13751*	FBgn0033340
*P{EP}CG8155^G2736^*	hom	105	6	92	71	**0.32**	2.0E-04	7.2E-11	*CG8155*	FBgn0034009
*P{EP}CG3838^G18268^*	hom	57	6	33	44	**0.41**	3.3E-03	9.8E-05	*CG3838*	FBgn0032130
*P{EP}Ipk2^G3545^*	hom	135	12	141	135	**0.41**	2.9E-07	1.7E-14	*Ipk2*	FBgn0031267
*P{EP}nopo^G5845^*	hom	59	6	48	50	**0.44**	3.3E-03	6.7E-05	*nopo*	FBgn0034314
*P{EP}inv^GE10665^*	hom	76	1	49	4	**0.46**	7.7E-06	2.6E-11	*inv*	FBgn0001269
*P{EP}lea^EP2582^*	hom	54	1	106	12	**0.47**	2.5E-04	7.1E-08	*lea*	FBgn0002543
*P{EP}zf30C^G2920^*	hom	64	1	68	6	**0.50**	4.8E-05	2.3E-09	*zf30C*	FBgn0022720
*P{EP}EP437*	hom	71	6	65	39	**0.51**	6.7E-03	2.6E-06	*GEFmeso*	FBgn0050115
*P{EP}coro^GE15547^*	hom	84	13	45	64	**0.52**	9.2E-03	1.0E-04	*coro*	FBgn0265935
*P{EP}spict^EP2202^*	hom	94	19	37	79	**0.54**	9.4E-03	1.0E-03	*spict*	FBgn0032451
*P{EP}CG12484^EP2424^*	hom	99	12	77	61	**0.57**	2.4E-04	8.0E-15	*CG12484*	FBgn0086604
*P{EP}AGO1^G2846^*	hom	81	10	67	52	**0.59**	3.2E-03	1.8E-05	*AGO1*	FBgn0262739
*P{EP}Pka-R2^EP2162^*	hom	117	18	72	73	**0.60**	5.3E-03	3.7E-06	*Pka-R2*	FBgn0022382
*P{EP}CG3358^G7969^*	hom	71	14	35	49	**0.62**	9.3E-03	4.4E-03	*CG3358*	FBgn0033117
*P{EP}CG3476^G2643^*	hom	103	17	76	77	**0.63**	4.0E-03	1.3E-10	*CG3476*	FBgn0031881
*P{EP}spi^EP2378^*	het	90	10	137	59	**0.63**	7.9E-04	1.4E-11	*spi*	FBgn0005672
*P{EP}RtGEF^G3647^*	hom	51	9	95	105	**0.63**	5.3E-03	3.2E-06	*RtGEF*	FBgn0015803
*P{EP}uzip^EP1153^*	hom	113	21	67	73	**0.66**	8.5E-03	1.9E-08	*uzip*	FBgn0004055
*P{EP}Arf51F^EP2612^*	hom	136	22	110	91	**0.68**	1.5E-03	3.2E-13	*Arf51F*	FBgn0013750
*P{EP}homer^EP2141^*	hom	104	18	67	59	**0.69**	9.5E-03	8.8E-09	*homer*	FBgn0025777
*P{EP}Oatp30B^EP2644^*	hom	94	16	91	77	**0.70**	7.8E-03	3.9E-08	*Oatp30B*	FBgn0032123
*P{EP}G19340*	hom	128	24	110	96	**0.73**	7.5E-03	9.9E-08	*Gbp*	FBgn0034199
*P{EP}G3678*	hom	124	23	114	85	**0.79**	9.7E-03	2.6E-07	*CG13220*	FBgn0033608
*P{EP}CG12744^GE13306^*	hom	65	43	30	29	**1.27**	9.6E-03	4.7E-03	*CG12744*	FBgn0033459
*P{EP}stau^G2796^*	hom	101	65	69	70	**1.28**	2.2E-03	1.4E-03	*stau*	FBgn0003520
*P{EP}lola^EP2537^*	het	91	38	106	51	**1.28**	8.5E-03	6.3E-03	*lola*	FBgn0005630
*P{EP}G2086*	hom	69	48	60	59	**1.28**	8.1E-03	2.1E-03	*Spp*	FBgn0031260
*P{EP}Sep5^G6155^*	hom	50	55	42	98	**1.31**	3.8E-06	1.2E-07	*Sep5*	FBgn0026361
*P{EP}EP2520*	het	116	59	97	46	**1.34**	2.4E-03	2.4E-03	*Lar*	FBgn0000464
*P{EP}E(Pc)^EP608^*	hom	86	55	99	109	**1.34**	2.5E-03	1.4E-03	*E(Pc)*	FBgn0000581
*P{EP}Thiolase^G3215^*	hom	69	56	56	55	**1.35**	1.1E-03	9.1E-04	*Thiolase*	FBgn0025352
*P{EP}EDTP^EP2390^*	hom	68	69	78	84	**1.37**	1.4E-03	2.2E-04	*EDTP*	FBgn0027506
*P{EP}G12586*	hom	82	67	82	94	**1.39**	7.9E-03	6.7E-03	*CG13551*	FBgn0040660
*P{EP}CG43340^G19466^*	hom	71	63	92	88	**1.40**	2.9E-04	7.3E-05	*CG43340*	FBgn0263077
*P{EP}Elba2^G17999^*	hom	54	44	88	76	**1.43**	2.3E-03	2.0E-03	*Elba2*	FBgn0031435
*P{EP}Socs36E^G2762^*	hom	93	76	70	58	**1.47**	5.3E-04	1.1E-04	*Socs36E*	FBgn0041184
*P{EP}EP993*	hom	51	44	68	57	**1.48**	1.3E-03	1.1E-03	*CG30069*	FBgn0050069
*P{EP}EP2515*	het	67	44	98	39	**1.59**	5.8E-03	3.2E-03	*dnr1*	FBgn0260866
*P{EP}pAbp^EP310^*	hom	78	41	106	57	**1.62**	5.5E-03	2.7E-03	*pAbp*	FBgn0265297
*P{EP}JhI-21^GE15185^*	hom	113	35	106	36	**1.74**	7.2E-03	4.9E-06	*Jhl-21*	FBgn0028425
*P{EP}G2213*	hom	61	35	173	54	**1.97**	7.4E-03	3.8E-03	***Chd1***	FBgn0250786
*P{EP}c(2)M^EP2115^*	hom	43	52	42	21	**2.15**	1.3E-05	1.7E-06	*c(2)M*	FBgn0028525
*P{EP}Traf4^EP578^*	hom	84	5	91	0	**INF**	7.1E-04	1.1E-08	*Traf4*	FBgn0026319

Statistically significant results of the viability-based screen for genetic modifiers of *His1* are presented. *EP* Allele, the second chromosome *EP* insertion allele identifier; *EP* Males, the homozygous/heterozygous males used in the screen and control crosses; H1KD + *EP*: *Cy*, the number of balancer-carrying adult F1 progeny in the screen cross; H1KD + *EP*: *Cy^+^*, the number of *Act5C-GAL4*, *pINT-H1^4M^/P{EP}* adult F1 progeny; *EP*: *Cy*, the number of balancer-carrying adult F1 progeny in the control cross; *EP*: *Cy^+^*, the number of *Act5C-GAL4/P{EP}* adult F1 progeny; NE, normalized effect of the *EP* allele on H1KD adult viability; *P* (FET), probability value from the Fisher’s exact test; *P* (bin), probability value from the binomial test. A gene symbol (conventional gene name) and its associated Flybase ID are shown for genes that are affected by every particular *EP* allele.

When the same genes were mis-expressed in the H1 knockdown background (in crosses with *Act5C-GAL4*, *pINT-H1^4M^/SM5* mothers), the mean fraction of *Act5C-GAL4*, *pINT-H1^4M^/P{EP}* adults was ∼24% (∼48% of expected) for homozygous crosses and ∼21% for heterozygous crosses (∼63% of expected), consistent with the control numbers (Table 1). Note that the higher proportion of *Act5C-GAL4*, *pINT-H1^4M^/P{EP}* escapers in the heterozygous cross was due to the scoring method, when a portion of the balancer-containing animals also undergo H1 depletion (Figure 1B). Importantly, simultaneous depletion of H1 and UAS-driven mis-expression of target genes modified and often reversed the effects on viability. For instance, H1 knockdown partially rescued the lethality of *Traf4* mis-expression but reduced the viability of *MESK2*-expressing flies to ∼12% of expected (Table 2).

We hypothesized that the variable effect on viability by a combination of H1 depletion and ubiquitous mis-expression of certain genes reflects their specific genetic interactions. To systematically examine modifications of the phenotype across the whole collection of *EP* alleles, we performed two statistical tests to assess nonrandom effects on viability. First, in a binomial test, we assumed a normal distribution of viability numbers for both the screen (*Act5C-GAL4*, *pINT-H1^4M^*) and control (*Act5C-GAL4*) crosses and calculated *P* values for deviation from the expected numbers. (The “expected” numbers assume independent effects of H1 depletion and gene mis-expression on viability.) Second, we performed the Fisher’s exact test (which does not depend on the normal distribution of data) using a representative gene as a reference. Normalized effect (NE) numbers were used as the measure of gene-specific effects and signify increased or decreased viability for *Act5C-GAL4*, *pINT-H1^4M^/P{EP}* flies normalized to viability for *Act5C-GAL4/P{EP}* flies and median viability of *Act5C-GAL4*, *pINT-H1^4M^/P{EP}* flies across the whole collection of second chromosome *EP* alleles (in lieu of the wild-type isogenic control). The OR in both the Fisher’s exact and binomial tests strongly correlated with the calculated NE for each *EP* allele. We defined results as statistically significant when relative viability of H1-depleted adults was affected more than 25% by the *EP*-driven mis-expression (NE<0.8 or NE>1.25), and when *P* values calculated in both the Fisher’s exact and binomial tests were less than 0.01 (Table 2). Our analyses identified 41 *EP* alleles as significant enhancers and 20 as suppressors of the *His1* knockdown-induced lethality.

### Genetic enhancers and suppressors of *His1* effect on fly viability

To classify alleles that genetically interact with *His1*, we performed Gene Ontology (GO) term analysis using the NIH web-based toll DAVID (Huang da *et al.* 2009a,b) according to their putative molecular function and involvement in biological processes. This survey reveals a statistically significant enrichment of *EP* insertions that affect the expression of particular functional groups of genes (Figure 1C, Supporting Information, Table S1). These interactions strongly suggest that H1 plays important regulatory roles in the cognate biological processes.

Predictably, nine *EP* insertions (∼15% of the identified genetic interactors) are upstream of genes that control chromatin structure and chromosome organization/metabolism. Five of them are suppressors of H1 depletion-mediated lethality. It is possible that the reduction of H1 expression can be partially compensated by ubiquitous mis-expression of *EP*-driven genes due to their ability to modify chromatin structure and activity. For instance, we observe a strong positive genetic interaction between *His1* and *E(Pc)*, a member of the *PcG* gene family. It is possible that H1 is biochemically involved in Polycomb silencing and, vice versa, Polycomb-dependent repression and modification of chromatin structure may partly compensate for the global loss of H1. Interestingly, a biochemical link has been previously proposed between H1-mediated oligonucleosome fiber compaction and Polycomb silencing: H1 can stimulate the enzymatic activity of *Drosophila* PRC2 complex on a dispersed oligonucleosome substrate (Yuan *et al.* 2012) and that of a homologous human EZH2 complex on a dinucleosome substrate (Martin *et al.* 2006) *in vitro*. Our genetic screen in *Drosophila* has identified several additional putative effectors of chromatin organization as modifiers of *His1* phenotypes. For example, *Socs36E* genetically interacts with *Stat92E* (Singh *et al.* 2010), which codes for the single STAT protein in *Drosophila*. The newly discovered interaction between *His1* and *Socs36E* is consistent with the role that JAK/STAT pathway plays in the deposition of H1 protein into larval chromatin and in heterochromatic silencing (Xu *et al.* 2014). Also, mis-expression of Elba2, a subunit of an embryonic boundary complex Elba (Aoki *et al.* 2012), partially ameliorates the deleterious effect of H1 depletion, which suggests a putative role for the linker histone in the insulator-dependent repression of enhancer-promoter interactions *in vivo*.

Consistent with the role of H1 in gene regulation, 10 *EP* insertions that interact with *His1* (∼16%) potentially drive mis-expression of transcription factors and other DNA-binding proteins. Interestingly, a large group of genetic modifiers of *His1* [18 (or ∼30% of all modifiers)] have functions in the nervous system development and/or activity, and the majority of genetic interactions between *His1* and members of this group are negative. The enrichment of neuronal genes among *His1* interactors suggests that H1 may play a specialized role in regulation of their chromatin structure and activity. A chromatin-dependent epigenetic mechanism is known to be essential for silencing a broad range of neuronal genes in all non-neural tissues across metazoan evolution. A common repressor element, RE1/NRSE, in their regulatory regions binds a ubiquitous (outside of the nervous system) transcription factor REST/NRSF (Tramtrack in *Drosophila*), which in turn recruits a histone-modifying complex CoREST that can remove histone acetyl and H3K4-methyl active epigenetic marks (Andres *et al.* 1999; Dallman *et al.* 2004; Qureshi *et al.* 2010). In the future, it will be interesting to examine whether H1 specifically interacts with CoREST *in vitro* and *in vivo*.

*His1* also interacts genetically with genes involved in stress response–related signaling. H1 depletion–induced lethality is consistently modified by mis-expression of genes that are involved in immune and defense responses [eight *EP* alleles (∼13%)]. One model that would explain the modification of the H1 depletion-induced lethality by mis-expression of genes involved in stress responses is that H1 itself plays a role in tissue-specific regulation of many genes that belong to this class. Ubiquitous mis-expression of a single gene might amplify or diminish the deleterious effect of their simultaneous global de-regulation caused by the knockdown of H1 alone. Therefore, we examined the influence of strong H1 depletion on expression of immunity-related and stress response–related genes in larval salivary glands. We analyzed our previously reported microarray-based transcript profiling (Lu *et al.* 2013) using DAVID and discovered that the immunity-related and stress response–related genes are significantly enriched among target genes that are mis-regulated by H1 RNAi (Table 3). Few genes in these GO term classes were negatively affected by the H1 knockdown (5 compared with 116 that are significantly upregulated). Thus, H1 functions primarily to repress immunity-related and stress response–related genes.

**Table 3:** GO term analyses of transcriptional targets of H1: immune and stress response

GOTERM_BP_FAT	Count	*P*	Genes	Fold Enrichment
***UP***				
GO:0033554 cellular response to stress	61	4.0E-11	*Debcl*, *Mms19*, *CG9272*, *lok*, *nod*, *CG31953*, *XRCC1*, *Top3alpha*, *Snm1*, *tefu*, *Ku80*, *Ubc6*, *okr*, *Pms2*, *msn*, *Rrp1*, *Rev1*, *spn-B*, *CG5524*, *spn-A*, *Lig4*, *dre4*, *mei-9*, *rad50*, *mre11*, *Btk29A*, *CG11403*, *Gen*, *mus210*, *CG2183*, *CG6812*, *p53*, *DNApol-eta*, *Jafrac1*, *mus209*, *pic*, *egr*, *puc*, *Irbp*, *pyd*, *hay*, *Xbp1*, *agt*, *mus205*, *CG5316*, *mus201*, *Ercc1*, *Fen1*, *rpr*, *Msh6*, *Ssrp*, *CG9203*, *Tfb4*, *spel1*, *Chrac-14*, *tos*, *CG11329*, *mu2*, *mus308*, *Corp*, *gkt*, *Src64B*	2.4
GO:0006952 defense response	42	8.3E-04	*CG10433*, *SPE*, *Sr-CI*, *Tollo*, *egr*, *IM10*, *Pmi*, *cactin*, *vir-1*, *CG16799*, *CG10345*, *IM3*, *IM1*, *Tsf1*, *PPO1*, *TotB*, *GNBP2*, *GNBP1*, *TotA*, *TotC*, *TotF*, *PGRP-SA*, *PGRP-SC2*, *Drs*, *Hf*, *PGRP-LD*, *He*, *CecC*, *Hml*, *kn*, *emp*, *LysX*, *ECSIT*, *CG33470*, *Ect4*, *AttD*, *psh*, *PGRP-SB2*, *Traf-like*, *TotZ*, *Tep3*, *Tep4*, *PGRP-LC*, *CG13422*	1.7
GO:0006955 immune response	38	6.1E-03	*mus209*, *SPE*, *Sr-CI*, *Tollo*, *egr*, *IM10*, *CG3074*, *Pmi*, *vir-1*, *mbo*, *CG16799*, *IM3*, *IM1*, *PPO1*, *TotB*, *GNBP2*, *GNBP1*, *TotA*, *TotC*, *TotF*, *PGRP-SA*, *PGRP-SC2*, *Drs*, *Hf*, *PGRP-LD*, *CecC*, *He*, *Hml*, *kn*, *LysX*, *ECSIT*, *CG33470*, *Ect4*, *AttD*, *psh*, *PGRP-SB2*, *TotZ*, *dos*, *Tep4*, *PGRP-LC*	1.5
GO:0009411 response to UV	7	6.4E-03	*hay*, *TotZ*, *p53*, *TotB*, *TotA*, *TotC*, *TotF*	3.7
GO:0009636 response to toxin	8	4.9E-02	*Cyp4aa1*, *Ctp6a9*, *kraken*, *Cyp6a2*, *Cyp4ac1*, *Cyp12a4*, *GstE9*, *Cyp4ac2*	2.3
***DOWN***				
GO:0070301 cellular response to hydrogen peroxide	5	2.2E-02	*Prx6005*, *Jafrac2*, *Prx2540-1*, *Pxn*, *Prx2540-2*	4.3

Immunity-related, cellular defense and stress response genes that are regulated by H1 based on transcript profiling in salivary glands of H1-depleted L3 larvae (Lu *et al.* 2013) grouped in functional clusters. GOTERM_BP_FAT (lower levels of biological process ontology) terms are indicated. *P* values and fold enrichment were calculated using the NIH web-based tool DAVID. Only the GO term classes that exhibit *P*<0.05 for relative enrichment are shown. *UP*, genes upregulated more than two-fold in the H1 knockdown salivary glands relative to the control salivary glands; *DOWN*, genes downregulated more than two-fold.

### Immunity-related genes are regulatory targets of H1

**Figure 2:**
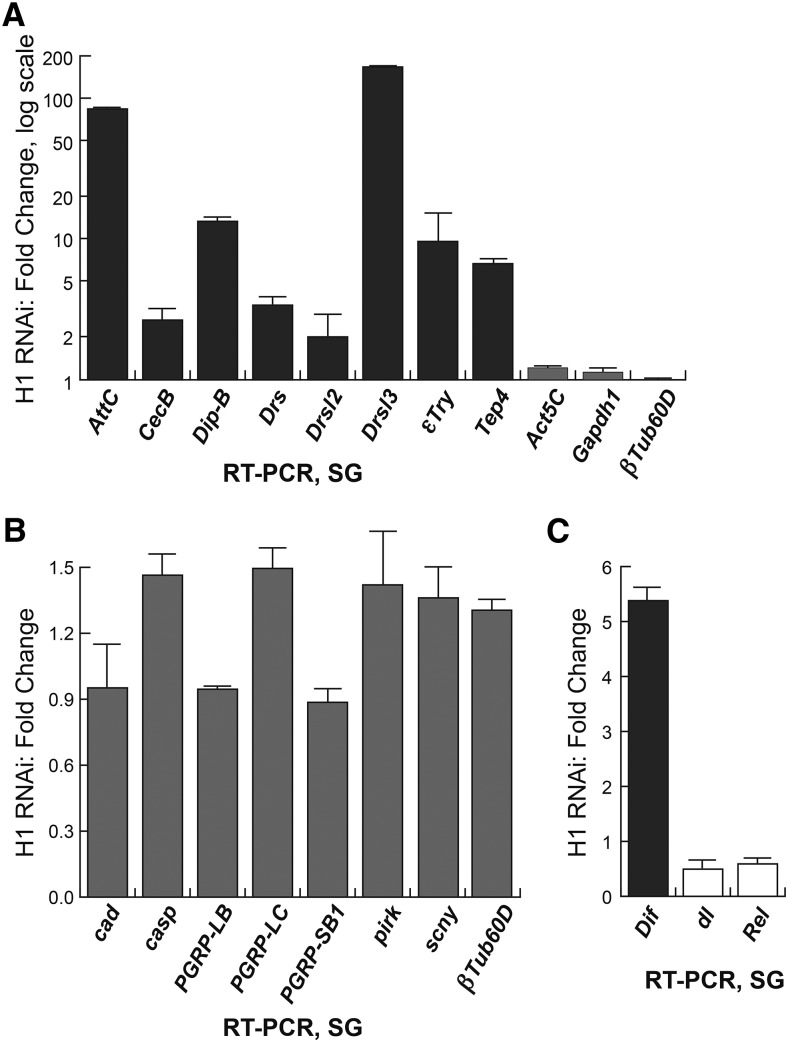
Regulation of immunity and stress response–related genes by H1. (A) Multiple immune response and stress response–related genes are upregulated in H1-depleted larval salivary glands. Real-time RT-PCR assays of transcripts were performed in H1-depleted and control salivary glands. Fold changes are calculated as a ratio of signals for H1-depleted samples to those for control samples. Values are normalized to the expression of *rp49*, are representative of three independent experiments, and are presented in a logarithmic scale. SDs are shown as error bars. Transcripts for *Act5C*, *Gapdh1*, and *βTub60D* are shown as controls. (B) Antimicrobial peptide (AMP) genes are not substantially affected by H1 knockdown in salivary glands. Real-time RT-PCR assays of transcripts were performed in H1-depleted and control salivary glands, analyzed, and presented as in (A). Transcript for *βTub60D* is shown as a control. (C) NFκB-family transcription factors Dorsal (*dl*), Dorsal-related immunity factor (*Dif*), and Relish (*Rel*) are not coordinately regulated on H1 depletion in salivary glands. Real-time RT-PCR assays of transcripts were performed in H1-depleted and control salivary glands, analyzed, and presented as in (A). Black, white, and gray bars indicate transcripts that are significantly upregulated, downregulated, or not affected, respectively, in the H1 knockdown.

To confirm the effects of H1 on these genes, we performed quantitative RT-PCR to compare mRNA expression of some of the immune response–related genes in H1-depleted and control salivary glands. We discovered a consistent correspondence between the microarray and RT-PCR data: genes that were detected as repression targets of H1 by microarray analyses also exhibited upregulation in H1-depleted salivary glands when examined by RT-PCR (Figure 2A and data not shown). Many additional immune response–related genes that were not originally identified as H1 targets by microarray analyses also exhibit strong upregulation (Figure 2A). It is of interest that many of these genes are repressed by a chromatin-remodeling factor CHD1 (Sebald *et al.* 2012), and *Chd1* was identified as one of the *His1*-interacting genes in our mis-expression screen (Table 2).

Considering a strong correlation between CHD1-mediated and H1-mediated repression of the immune response–related genes and the genetic interactions between *Chd1* and *His1*, we expected that *Chd1* and *His1* would share other features of their transcriptional regulatory programs. For instance, antimicrobial peptide (AMP) genes are strongly repressed in *Chd1/Chd1* null larvae (Sebald *et al.* 2012). However, when we analyzed the expression of AMP genes by real-time RT-PCR in H1-depleted samples, we observed little or no effect (Figure 2B). Thus, H1 and CHD1 appear to share regulatory functions for some but not all categories of gene targets, and the repression of multiple AMP genes in the *Chd1* mutant is not phenocopied by H1 depletion.

Also, the concerted regulation of immune response genes by CHD1 and H1 may depend on their similar roles in regulation of the Toll and immune deficiency (IMD) pathways through master transcription factors of NFκB-family, Dorsal, Dif, and Relish (Hetru and Hoffmann 2009). For instance, *dl*, but not other NFκB-related genes, is upregulated in *Chd1* mutant larvae (Sebald *et al.* 2012). This suggests that CHD1 and H1 might regulate multiple immunity-related genes through their coordinate regulation of Dorsal expression. However, when we analyzed the transcripts of *Drosophila* NFκB factors in H1-depleted salivary glands by RT-PCR, we did not observe regulatory effects consistent with this hypothesis: the expression of *Dif* was moderately elevated, whereas *dl* and *Rel* were downregulated (Figure 2C). Therefore, the concerted repression of immune response genes by H1 and CHD1 likely depends on NFκB-independent regulatory mechanisms.

### *His1* and *Chd1* share a subset of regulatory target genes in *Drosophila* genome-wide

It is important that in our genetic screen, ubiquitous mis-expression of *Chd1* suppresses H1 depletion-dependent reduction in fly viability. Thus, we expected that reduced expression of CHD1 in homozygous *Chd1* null progeny of *Chd1/CyO inter se* crosses would enhance the semi-lethal phenotype caused by RNAi-mediated H1 depletion. To test this hypothesis, we crossed *Chd1^1^/CyO*; *pINT-H1^1M^* and *Chd1^1^/CyO*; *Tub-GAL4/TM3*, *Sb* parents at various temperatures and scored the relative viability of *Chd1^1^* homozygotes among *Sb^+^* (H1-depleted) progeny (Table 4). We again observed a strong genetic interaction between *His1* and *Chd1*. In particular, at 26° *Chd1* mutation significantly enhanced the reduced viability of H1-depleted animals. Combined, these results suggest a strong link between the biological functions of CHD1 and H1 *in vivo*.

**Table 4:** Genetic interactions of *Chd1* and *His1*

Scored Genotypes	Temperature			
	18°	22°	26°	29°
*Chd1^1^/Chd1^1^*; *pINT-H1^1M^/Tub-GAL4 vs. Chd1^1^/CyO*; *pINT-H1^1M^/Tub-GAL4*	12/112 (32%)	5/151 (10%)	1/83 (4%)	0/4 (0%)
*Chd1^1^/Chd1^1^ vs. Chd1^1^/CyO*	16/118 (41%)	15/170 (26%)	17/163 (31%)	16/142 (34%)
*P*	0.51	0.041	0.0086	0.50

Double-heterozygous *Chd1^1^/CyO*; *Tub-GAL4/TM3*, *Sb* females were mated to *Chd1^1^/CyO*; *pINT-H1^1M^/pINT-H1^1M^* males. The crosses were set at indicated temperatures. The effect of *Chd1* on viability in the H1 knockdown background was scored as the number of eclosed *Cy^+^*, *Sb^+^* adults relative to the number of all *Sb^+^* siblings. In control crosses, *Chd1^1^/CyO* flies were mated *inter se*, and relative numbers of eclosed *Cy^+^* adults were scored relative to the total number of F1 progeny. Percent viability compared with the expected number calculated from the Mendelian distribution is shown in parentheses. *P* values are calculated by the chi-square two-way test.

**Figure 3:**
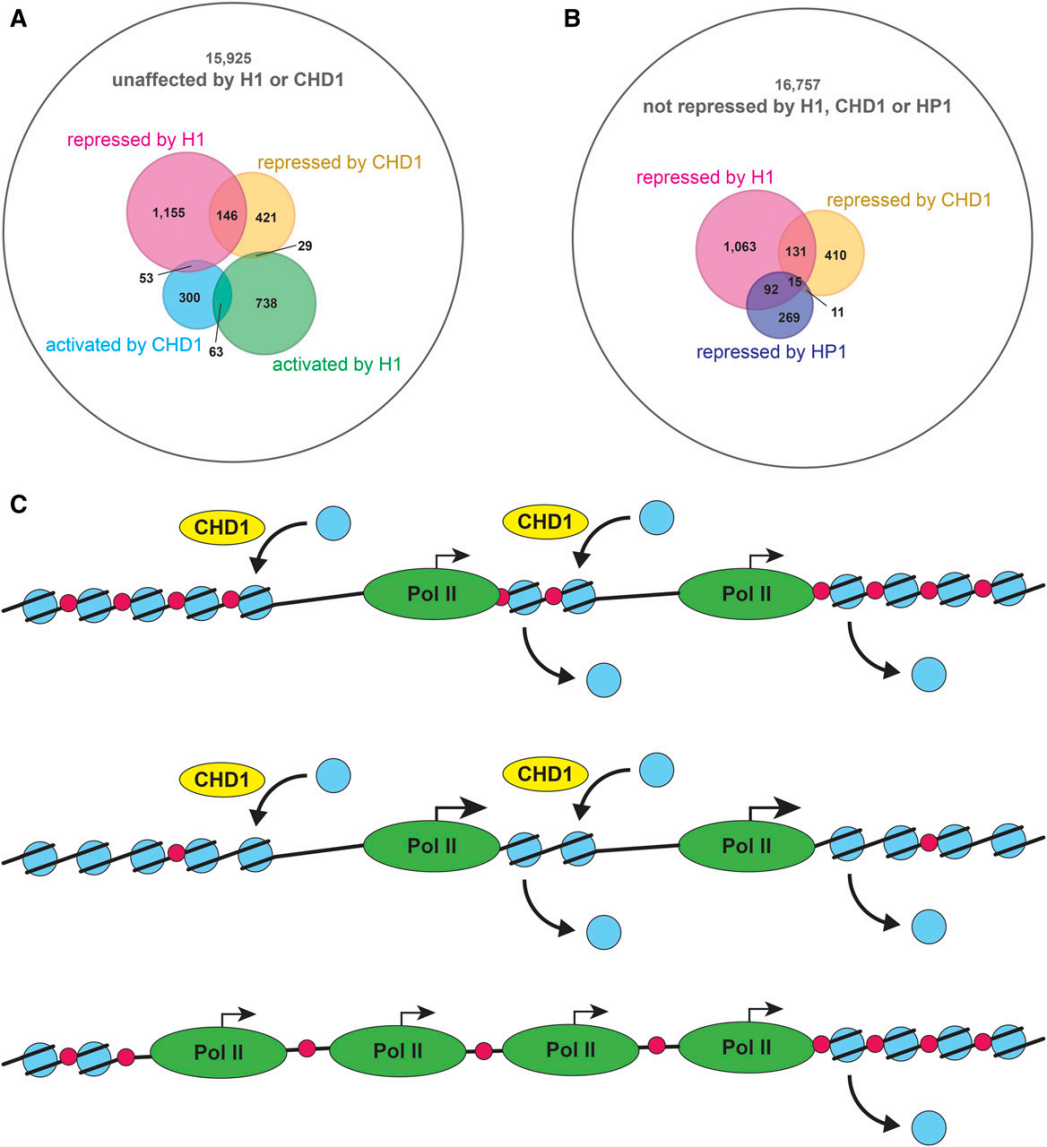
Shared transcriptional repression programs of H1 and CHD1. (A) Transcripts that are demonstrated by microarray analyses to be repressed by H1 or CHD1 in *Drosophila in vivo* exhibit a significant overlap. Circles in the Venn diagram show all transcripts spotted on the microarray (white), upregulated (pink) or downregulated (green) in the H1 knockdown, and upregulated (yellow) or downregulated (cyan) in the *Chd1* mutant. Numbers represent transcripts of each overlapping or nonoverlapping class. Note that 146 transcripts are upregulated in both the H1 knockdown and *Chd1* mutant. (B) Transcripts that are demonstrated by microarray analyses to be repressed by HP1 or CHD1 in *Drosophila in vivo* do not exhibit a highly significant overlap. Circles in the Venn diagram show all transcripts spotted on the microarray (white), upregulated in H1 knockdown (pink), upregulated in *Chd1* mutant (yellow), or upregulated in HP1 knockdown (purple). Numbers represent transcripts of each overlapping or nonoverlapping class. (C) A model for coordinate gene regulation by H1 and CHD1 at the level of transcriptional elongation by RNA polymerase 2. (Top) Wild-type chromatin. (Middle) H1-depleted chromatin. (Bottom) *Chd1* mutant chromatin. Blue circle, core histone octamer; red circle, H1; yellow oval, CHD1; green oval; RNA polymerase 2. Straight arrows indicate levels of transcription by RNA polymerase 2; curved arrows indicate transcription-linked nucleosome dis-assembly and CHD1-dependent re-assembly.

We decided to examine a possible correlation between global, genome-wide transcript profiles of *Chd1* null and H1-depleted *Drosophila* tissues. To this end, we cross-referenced transcripts that are upregulated or downregulated at least two-fold in *Chd1/Chd1* mutant whole larvae (Sebald *et al.* 2012) and larval salivary glands strongly depleted of H1 (Lu *et al.* 2013). The observed correlation is striking. Of the 1012 transcripts that are affected by *Chd1*, 291 (28.8%) are also affected by H1 depletion (Table S2), which represents ∼2.5 enrichment compared with the total proportion of H1-affected gene transcripts [2174 of 18,833 arrayed transcripts (11.5%)]. The most significant correlation is between transcripts that are repressed by CHD1 (596) and H1 (1354): of these, 146 appear to be repressed by both CHD1 and H1, which represents ∼3.4-fold enrichment (*P*=2.3⋅10^−41^). For comparison, no significant correlation is observed between CHD1-repressed and H1-activated transcripts [29 of 596 (∼1.1-fold enrichment) *P*=0.32]. Thus, CHD1 and H1 exhibit an extremely strong overlap of their repressive roles in global transcriptional regulation (Figure 3A). This overlap is not limited to a particular class of gene targets but is prevalent genome-wide. Thus, it is highly likely that the observed genetic interactions between *His1* and *Chd1* result from their coordinate regulation of a wide range of fly genes.

The overlap between repression programs of CHD1 and H1 does not necessarily indicate that there are specific functional interactions between the two proteins. Instead, it may reflect a common mode of action for many factors that regulate transcription at the level of chromatin structure. To examine this possibility, we performed similar correlation analyses for transcriptional repression targets of two other chromatin effectors for which microarray data are available: from RNAi-dependent depletion of heterochromatin factor 1, HP1, in *Drosophila* Kc cells (Schwaiger *et al.* 2010) and RNAi knockdown of chromatin remodeler ISWI in *Drosophila* SL2 cells (Bonaldi *et al.* 2008). Both factors are likely to share regulatory roles with H1: HP1 and H1 physically interact and are known to participate in heterochromatin-related gene silencing (Lu *et al.* 2013), whereas ISWI was shown to be required for H1 deposition into chromatin (Corona *et al.* 2007; Siriaco *et al.* 2009). Accordingly, we discovered a strong correlation between their respective transcriptional repression programs. Of 397 transcripts that are de-repressed at least two-fold in the HP1 knockdown, 107 are also de-repressed more than two-fold in the H1 knockdown [27.0% (∼3.7-fold enrichment) *P*=2.6⋅10^−34^] (Table S3). As expected, the transcripts co-regulated by H1 and HP1 are enriched for transposable elements and repetitive/heterochromatic sequences (Lu *et al.* 2013). Similarly, of 271 transcripts upregulated in the ISWI knockdown, 64 are upregulated in the H1 knockdown [23.6% (∼3.3-fold enrichment) *P*=9.7⋅10^−18^] (Table S4). In contrast, a similar very strong correlation is not observed between CHD1-dependent and HP1-dependent or ISWI-dependent repression. Of 596 CHD1-repressed transcripts, only 26 are also HP1-repressed (∼2.1-fold enrichment, *P*=4.1⋅10^−4^), and 16 are also ISWI-repressed (∼1.9-fold enrichment, *P*=0.013) (Table S2). Note that a somewhat elevated correlation between transcriptional programs of CHD1 and HP1 may represent a secondary, indirect effect of a strong link between a regulatory program of H1 with those of both CHD1 and HP1, because 15 out of 26 transcripts (57.7%) that are coordinately repressed by CHD1 and HP1 are also subject to transcriptional repression by H1 (Table S2, Figure 3B). Thus, there is a very strong functional coordination of H1-dependent transcriptional repression program with that of CHD1, which is not shared with other factors of chromatin metabolism, such as HP1 or ISWI.

From both the genetic screen and analyses of transcript regulation in *Drosophila*, we observe a strong functional interaction *in vivo* between two major chromatin effectors, the linker histone H1 and ATP-dependent motor protein CHD1. Previous biochemical analyses failed to predict this association. Rather they suggested independent roles for H1 and CHD1 in regulation of chromatin structure and activity. In fact, it was shown that CHD1, unlike ISWI, is unable to assemble (Lusser *et al.* 2005) or remodel (Maier *et al.* 2008) H1-containing chromatin *in vitro*. Therefore, it is difficult to explain a shared spectrum of transcriptional targets of H1 and CHD1 by a direct biochemical link between the proteins. Rather, it is likely that the relationship arises from a common mechanism of transcriptional regulation of their target genes.

CHD1 was postulated to participate in transcriptional repression at the level of elongation via its ability to re-assemble nucleosomes in the wake of RNA polymerase 2 during active transcription (Konev *et al.* 2007; Vanti *et al.* 2009; Smolle *et al.* 2012; Zentner *et al.* 2013), which necessitates a transient unraveling of chromatin and removal of nucleosomes (Kulaeva *et al.* 2010). A large number of gene targets, including immune response–related genes (Figure 2, Table S2) that are affected by CHD1 and H1 are not constitutively active but instead are strongly regulated in development or in response to external stimuli. It is possible that the principal mechanism of repression for these genes is at the level of elongation. In response to the stimuli under normal conditions (in wild-type), particular features of their promoters, such as *cis*-acting elements, may rapidly recruit factors that stimulate Pol 2 elongation, help to obviate the barrier presented by nucleosomes reassembled by CHD1, and thus de-repress transcription. Importantly, linker histones interspersed on nucleosomes within a transcription unit are expected to create an additional obstacle for the polymerase during transcriptional elongation (Figure 3C). Thus, the elimination of either CHD1 or H1 can spuriously activate these target genes. Alternatively, strong co-regulation of targets by H1 and CHD1 may rely on additional mechanisms. For instance, the common targets may share specific modifications of the epigenetic landscape in their transcription units, which favor the recruitment of both H1 and CHD1. The latter model does not necessitate that co-regulation of target genes by both proteins take place specifically at the level of elongation.

In conclusion, we have previously characterized linker histone H1 as a major determinant of heterochromatin identity. Through physical interactions with HP1 and histone methyltransferase Su(var)3-9, H1 contributes to the establishment of the silenced epigenetic state (Lu *et al.* 2013). In this study, we discover a novel regulatory pathway that H1 utilizes to repress genome-wide genetic activity *in vivo*, and it is not linked to heterochromatin-type silencing or heterochromatin effector proteins. Rather, this regulatory program mediated by H1 is shared with the chromatin remodeling motor protein CHD1.

## 

## Supplementary Material

Supporting Information
