# Supplementary material for: A Genetic Screen and Transcript Profiling Reveal a Shared Regulatory Program for Drosophila Linker Histone H1 and Chromatin Remodeler CHD1
Source: G3 (Bethesda). 2015 Jan 27;5(4):677–87. doi: 10.1534/g3.115.016709 (PMC4390582; doi:10.1534/g3.115.016709)
Supplement: Supporting Information [file supp_5_4_677__index.html]

A Genetic Screen and Transcript Profiling Reveal a Shared Regulatory Program for Drosophila Linker Histone H1 and Chromatin Remodeler CHD1 — Supporting Information 

# A Genetic Screen and Transcript Profiling Reveal a Shared Regulatory Program for *Drosophila* Linker Histone H1 and Chromatin Remodeler CHD1

## Supporting Information for Kavi *et al.*, 2015

**Files in this Data Supplement:**

- Supporting Information - Tables S1-S4 and Supporting Information References (PDF, 368 KB)
- Table S1 - GO term analyses of genetic modifiers of *His1* effect on viability. (PDF, 184 KB)
- Table S2 - Overlap of transcripts regulated by CHD1, H1, HP1 and ISWI. (PDF, 245 KB)
- Table S3 - Overlap of transcripts regulated by HP1, H1 and CHD1. (PDF, 205 KB)
- Table S4 - Overlap of transcripts regulated by ISWI, H1 and CHD1. (PDF, 198 KB)
