# Supplementary material for: A Genetic Screen and Transcript Profiling Reveal a Shared Regulatory Program for Drosophila Linker Histone H1 and Chromatin Remodeler CHD1
Source: G3 (Bethesda). 2015 Jan 27;5(4):677–87. doi: 10.1534/g3.115.016709 (PMC4390582; doi:10.1534/g3.115.016709)
Supplement: Supporting Information [file supp_g3.115.016709_016709SI.pdf]

G3 · Genes | Genomes | Genetics

A Genetic Screen and Transcript Profiling Reveal a Shared Regulatory Program for *Drosophila* Linker Histone H1 and Chromatin Remodeler CHD1

Harsh Kavi, Xingwu Lu, Na Xu, Boris A. Bartholdy, Elena Vershilova, Arthur I. Skoultchi[4], and Dmitry V. Fyodorov[4]

Department of Cell Biology, Albert Einstein College of Medicine, 1300 Morris Park Avenue, Bronx, NY 10461, USA

[4] Corresponding authors: Department of Cell Biology, Albert Einstein College of Medicine, 1300 Morris Park Avenue, Bronx, NY 10461, USA. E-mail: arthur.skoultchi@einstein.yu.edu, dmitry.fyodorov@einstein.yu.edu

DOI: 10.1534/g3.115.016709

Table S1 GO term analyses of genetic modifiers of *His1* effect on viability.

Gene Symbol	Biological evidence for the gene and/or gene product	Reference(s)
**Chromosome organization (biological function), GO:0051276**		
nopo	Fly homolog of human E3 ligase TRIP; interacts with DNA polymerase η, regulates S to M transition during syncytial nuclear divisions.	(Wallace et al. 2014)<br>(Merkle et al. 2009)
AGO1	Component of miRNA-specific silencing machinery; was shown to regulate nuclear organization of PcG target loci.	(Williams and Rubin 2002)<br>(Grimaud et al. 2006)
CG3358	Fly ortholog of TatD-related DNAse TATDN1 that plays an important role in mitotic chromosome segregation.	(Yang et al. 2012)
Sep5	Nuclear GTPase and a component of microtubule-associated complex involved in cytokinesis.	(Hughes et al. 2008)<br>(Goldstein and Gunawardena 2000)
E(Pc)	Contributes to chromatin-dependent silencing; is not present in PRC1 or PRC2 but, rather, is a subunit of histone acetyltranferase complex TIP60 involved in both gene activation and repression; interacts with *ISWI*, which is involved in H1 deposition into chromatin.	(Sinclair et al. 1998)<br>(Stankunas et al. 1998)<br>(Kusch et al. 2004)<br>(Arancio et al. 2010)
Elba2	BEN domain-containing protein; associates with the *Fab-7* boundary/insulator element, promotes chromatin silencing.	(Aoki et al. 2012)
Socs36E	SH2 domain-containing suppressor of cytokine signaling; a negative regulator of the JAK/STAT cascade, which is involved, together with H1, in the establishment of heterochromatin silencing.	(Singh et al. 2010)<br>(Xu et al. 2014)
c(2)M	Component of the synaptonemal complex; required for reciprocal meiotic recombination.	(Heidmann et al. 2004)
Chd1	ATP-dependent nucleosome remodeling motor protein; essential for chromatin assembly in the nascent male pronucleus and for deposition of H3.3 at transcriptionally active loci.	(Konev et al. 2007)
**DNA binding (molecular function), GO:0003677**		
bsh	Brain-specific homeobox protein.	(Hasegawa et al. 2013)
vis	Homeobox-containing TGIF-related transactivator.	(Hyman et al. 2003)
inv	Helix-turn-helix transcription factor.	(Simmonds et al. 1995)
zf30C	Zinc finger transcription factor.	(Jafari et al. 2012)
CG3358	Fly ortholog of TatD-related DNAse TATDN1.	(Yang et al. 2012)
CG12744	Zinc finger transcription factor.	(Jin et al. 2008)
lola	Zinc finger transcription factor.	(Giniger et al. 1994)
Elba2	BEN domain-containing protein, associates with the *Fab-7* element.	(Aoki et al. 2012)
Chd1	ATP-dependent nucleosome remodeling motor protein.	(Konev et al. 2007)
c(2)M	Component of the synaptonemal complex.	(Heidmann et al. 2004)
**Small GTPase-mediated signal transduction (biological function), GO:0007264**		
phyl	Regulator of Notch and Wnt signaling pathways.	(Nagaraj and Banerjee 2009)
CG15611	Exhibits sequence similarity to fly Rho guanyl-nucleotide exchange factor TRIO.	(Bateman et al. 2000)
CG8155	Structurally similar to fly Rab GTPase activator Evi5.	(Laflamme et al. 2012)
GEFmeso	Mesoderm guanine nucleotide exchange, a binding target of Ras-like GTPase Ral.	(Blanke and Jackle 2006)
RtGEF	Homologous to mammalian Rho-type guanyl-nucleotide exchange factor Pix.	(Manser et al. 1998)
Arf51F	GTP-binding protein according to sequence similarity to human ADP-ribosylation factor 2, Arf2.	(Bobak et al. 1989)
Sep5	Belongs to the superfamily of Septin GTPases.	(O'Neill and Clark 2013)
**Gametogenesis (biological function), GO:0048477, GO:0007283**		
gbb	Fly ortholog of human bone morphogenic protein-5 (BMP-5); was shown to be involved in BMP signaling that controls ovarian cell development.	(Kirilly et al. 2005)
vis	Transcription factor; required for regulation of multiple genes involved in sperm manufacture; mutants are male sterile due to a defect in primary	(Ayyar et al. 2003)

	spermatocyte differentiation before the onset of meiotic divisions.	
TBCB	Tubulin-binding cofactor B, a ubiquitin-related domain-containing protein; regulates microtubule organization in the nucleus and controls oocyte polarity.	(Baffet et al. 2012)
AGO1	Plays central roles in female germ-line cell differentiation.	(Yang et al. 2007)
Arf51F	ADP ribosylation factor; is required for cytokinesis in spermatocytes.	(Dyer et al. 2007)
stau	dsRNA- and mRNA 3'-UTR-binding protein; regulates translation and mRNA localization of egg polarity genes *bicoid*, *gurken*, *nanos* and *oskar*.	(Johnstone and Lasko 2001)
Lar	Leukocyte antigen-related-like tyrosine phosphatase receptor protein; required for the establishment of cell polarity during oogenesis.	(Krueger et al. 2003)
Edtp	Egg-derived tyrosine phosphatase; essential for ovarian development, oogenesis and embryogenesis.	(Yamaguchi et al. 2005)
Socs36E	Functions in male germ-line stem cell maintenance.	(Issigonis et al. 2009)<br>(Singh et al. 2010)
pAbp	Polyadenylate-binding protein; essential for spermatid elongation; translationally regulates Grk (*gurken*), which is required for the establishment of the dorsal-ventral axis of a developing egg.	(Pertceva et al. 2010)<br>(Clouse et al. 2008)
Chd1	SNF2-like ATPase; functions in oogenesis and egg fertilization.	(Konev et al. 2007)<br>(McDaniel et al. 2008)
**Nervous system development and neural system processes (biological function), GO:0007399, GO:0050877**		
Dap160	Dynamin-associated protein, contains EF-hand and SH3 domains, a negative regulator of Notch signaling; interacts with the atypical protein kinase C (aPKC) to stimulate neuroblast proliferation; similar to dynamin, regulates synaptic vesicle endocytosis.	(Chabu and Doe 2008)<br>(Koh et al. 2004)
bsh	Transcription factor; regulates neuronal identity specification.	(Hasegawa et al. 2013)
phyl	Zinc-binding protein; essential for the peripheral nervous system development and photoreceptor differentiation.	(Chang et al. 1995)
gbb	Part of the BMP signaling pathway; important for the neuromuscular junction development and synaptic transmission.	(James and Broihier 2011)
CG5742	Predicted ankyrin repeat-containing protein; was found to regulate neurogenesis in an RNAi screen in vivo in *Drosophila*.	(Neumuller et al. 2011)
inv	Transcription factor; regulates neuroblast fate determination.	(Bhat and Schedl 1997)
lea	Axon guidance receptor; regulates neuron migration.	(Simpson et al. 2000)<br>(Kraut and Zinn 2004)
zf30C	Transcription factor; regulates dendrite morphology in the developing brain.	(Parrish et al. 2006)
spict	Magnesium transmembrane transporter orthologous to human NIPA1; negatively regulates BMP signaling to promote synaptic growth at the neuromuscular junction.	(Wang et al. 2007)
Pka-R2	cAMP-dependent protein kinase R2; functions in repulsive axon guidance.	(Terman and Kolodkin 2004)
spi	TGF-α-like ligand that triggers epidermal growth factor receptor (EGFR) activation to promote neuroepithelial proliferation and neuroblast formation in the optic lobe; as a component of EGFR signaling pathway, is important for olfactory learning in flies.	(Morante et al. 2013)<br>(Rahn et al. 2013)
RtGEF	Rho-type guanine nucleotide exchange factor; regulates postsynaptic structure and protein localization at the glutaminergic neuromuscular junction.	(Parnas et al. 2001)
uzip	Cell adhesion protein; functions in axon guidance in the *Drosophila* central nervous system.	(Ding et al. 2011)
Arf51F	GTP-binding protein; has a regulatory function in synaptic vesicle endocytosis.	(Lloyd et al. 2000)
stau	Regulator of translation, a major player in the establishment of long-term memory and neuronal plasticity.	(Dubnau et al. 2003)
lola	Transcription factor; regulates axonogenesis, brain morphogenesis and eye development.	(Giniger et al. 1994)<br>(Yamamoto et al. 2008)<br>(Zheng and Carthew 2008)

Lar	Tyrosine phosphatase receptor; plays a key role in the control of axon guidance.	(Krueger et al. 1996)
E(Pc)	Together with other subunits of the TIP60 HAT complex regulates dendrite targeting of olfactory neurons.	(Tea and Luo 2011)
**Stress response (biological function), GO:0006955, GO:0006952**		
nopo	E3 ligase; likely plays a role in defense response, based on its homology with human TRAF-interacting protein TRIP.	(Lee et al. 1997)
coro	WD40 repeat-containing protein; belongs to a family of proteins involved in defense response to fungus.	(Jin et al. 2008)
Gbp	Growth blocking peptide; a potent cytokine that stimulates the expression of anti-microbial peptides.	(Tsuzuki et al. 2012)
lola	Transcription factor; functions in anti-microbial humoral response.	(Kleino et al. 2005)
CG13551	ATPase inhibitor, glycine-rich protein; has an in vitro activity in suppressing Gramm-positive bacteria.	(Feng et al. 2009)
dnr1	FERM domain- and RING finger-containing protein; an inhibitor of the immune deficiency (IMD) pathway at the level of initiator caspase Dredd.	(Guntermann et al. 2009)
Jhl-21	Leucine transmembrane transporter; belongs to a family of proteins involved in defense response to fungus.	(Jin et al. 2008)
Traf4	TNF receptor-associated factor; functions in the IMD pathway and plays a role in fly defense response to *Vibrio cholera*.	(Berkey et al. 2009)

The known attributes and properties of genetic suppressors and enhancers of *His1* function in vivo that belong to enriched GO term classes are listed.

Table S2 Overlap of transcripts regulated by CHD1, H1, HP1 and ISWI.

SYMBOL/ TRANSCRIPT	FLYBASE ID	FOLD CHANGE			
		Chd1	**H1 KD**	**HP1 KD**	**ISWI KD**
Chd1* effect: *UP					
**detoxification**					
Gclc	FBgn0040319	**2.04**			
GstT4	FBgn0030484	**2.11**	**2.14**	**2.28**	
Cyp12b2	FBgn0034387	**2.22**			
Cyp4ac3	FBgn0031695	**2.25**	**29.44**		
Cyp18a1	FBgn0010383	**2.28**			
Cyp28a5	FBgn0028940	**2.30**	**2.27**		
Cyt-b5-r	FBgn0000406	**2.57**	**10.17**		**0.393**
GstD6	FBgn0010042	**2.68**	**13.24**		
CG4623	FBgn0035587	**3.16**			
Cyp9c1	FBgn0015040	**3.41**		**6.66**	
Cyp6a18	FBgn0039519	**3.81**			
Ugt86Dj	FBgn0040250	**3.92**			
Cyp9h1	FBgn0033775	**4.06**			
GstE6	FBgn0063494	**4.17**	**43.50**	**2.95**	
Cyp313a3	FBgn0038007	**5.03**			
Cyp6a9	FBgn0013771	**5.28**	**2.30**		
Cyp4d8	FBgn0015033	**5.74**	**2.40**		
Cyp6a23	FBgn0033978	**5.98**	**16.25**		**3.07**
GstE7	FBgn0063493	**8.94**		**3.78**	
GstE5	FBgn0063495	**12.3**	**29.07**		
Cyp6a2	FBgn0000473	**30.5**		**2.21**	
Cyp4p2	FBgn0033395	**40.2**	**4.52**		
Cyp4e3	FBgn0015035	**73.0**	**7.07**		
**development**					
Iris	FBgn0031305	**2.00**			
Idgf3	FBgn0020414	**2.00**	**3.07**		
hts	FBgn0263391	**2.04**			
CG13461	FBgn0036468	**2.14**	**5.69**		
Rbp9	FBgn0010263	**2.23**	**2.02**		
rad50	FBgn0034728	**2.27**	**7.96**		
PH4alphaSG2	FBgn0039779	**2.35**			
mbl	FBgn0265487	**2.45**			
CR_TC_RE65113		**2.51**			
CG42390	FBgn0259736	**2.51**	**0.464**		**2.48**
CG15784	FBgn0029766	**2.68**	**3.42**		
Npc1b	FBgn0261675	**2.77**			
bol	FBgn0011206	**2.91**			
Glycogenin	FBgn0265191	**2.95**			
lola	FBgn0005630	**3.12**			
CG14265	FBgn0040393	**3.36**			
chinmo	FBgn0086758	**3.46**			
ine	FBgn0011603	**3.51**	**0.062**		
sens	FBgn0002573	**3.58**			
Atf3	FBgn0028550	**3.81**			
JhI-26	FBgn0028424	**3.84**	**7.00**	**2.68**	
metro	FBgn0050021	**3.86**	**0.084**		

CG5867	FBgn0027586	**3.97**			
Est-Q	FBgn0037090	**4.06**	**5.17**		
Ftz-f1	FBcl0153222	**4.06**			
Dh44-R2	FBgn0033744	**4.23**	**2.33**		
chinmo	FBgn0086758	**4.35**			
CG10918	FBgn0031178	**4.76**			
smp-30	FBgn0038257	**4.76**	**19.82**		
tx	FBgn0263118	**4.86**			
fru	FBgn0004652	**5.13**	**2.85**		
smp-30	FBgn0038257	**5.46**	**19.82**		
CG32694	FBgn0052694	**5.54**			
Msp300	FBgn0261836	**5.74**			
lcs	FBgn0028583	**6.87**			
LP09368	FBcl0192001	**7.94**			
CG32071	FBgn0052071	**8.17**			
CG42368	FBgn0259714	**42.2**	**0.471**		
CG16898	FBgn0034480	**69.1**	**6.66**		
l(2)efl	FBgn0011296	**85.6**			
**immune response**					
kek5	FBgn0031016	**2.01**			
Ect4	FBgn0262579	**2.06**		**2.17**	
SPE	FBgn0039102	**2.19**	**4.83**		
Tsp42Eh	FBgn0033129	**2.20**			
Sr-CI	FBgn0014033	**2.46**	**4.37**		
CG8562	FBgn0035779	**2.57**			
dl	FBgn0260632	**2.57**			
ea	FBgn0000533	**2.60**			
Tsp42Eg	FBgn0033128	**2.62**			
ninaD	FBgn0002939	**2.71**			
PebIII	FBgn0011695	**2.85**			
Phae2	FBgn0263235	**2.85**			
alpha-Est2	FBgn0015570	**2.85**	**2.17**		
TBCB	FBgn0034451	**2.97**			
CG10433	FBgn0034638	**3.05**	**3.25**		**0.487**
CG5597	FBgn0034920	**3.07**			
Ect4	FBgn0262579	**3.12**		**2.17**	
NimB1	FBgn0027929	**3.18**			
CG15255	FBgn0028950	**3.46**			
NimC1	FBgn0259896	**3.51**			
CG3775	FBgn0030425	**3.53**			
CG5791	FBgn0040582	**3.58**			
Ser6	FBgn0011834	**3.78**			
CG13947	FBgn0031277	**4.14**	**2.26**		
CG7248	FBgn0036229	**4.23**			
CG5550	FBgn0034160	**4.38**			
PGRP-SC1b	FBgn0033327	**4.47**			
CG15293	FBgn0028526	**4.50**	**5.16**		
CG8620	FBgn0040837	**4.89**	**160.83**		
mthl8	FBgn0052475	**4.99**	**3.29**		
CG17974	FBgn0034624	**5.46**			
spheroide	FBgn0030774	**5.74**			
CG13324	FBgn0033789	**5.78**			

CG9928	FBgn0032472	**5.86**	**19.55**		
TotC	FBgn0044812	**6.15**	**58.64**		
CG18179	FBgn0036023	**6.32**			
Muc68Ca	FBgn0036181	**7.36**			**2.07**
CG33178	FBgn0053178	**7.36**			
CG5778	FBgn0038930	**7.57**	**27.36**		
Fmo-2	FBgn0033079	**9.00**	**9.77**		
CG14419	FBgn0029639	**12.1**			
CG16775	FBgn0036767	**13.9**			
Prx2540-2	FBgn0033518	**27.1**	**0.483**		
Mdr50	FBgn0010241	**54.6**			
**intracellular processes**					
larp	FBgn0261618	**2.01**			
Sec31	FBgn0033339	**2.01**	**0.483**		
dm	FBgn0262656	**2.03**			
stau	FBgn0003520	**2.06**			
mTTF	FBgn0028530	**2.10**			
qless	FBgn0051005	**2.10**			
Ndae1	FBgn0259111	**2.10**	**0.357**	**4.82**	
Nf1	FBgn0015269	**2.14**			
CG8503	FBgn0033917	**2.23**			
form3	FBgn0053556	**2.28**			
Msp300	FBgn0261836	**2.38**			
CG5599	FBgn0030612	**2.41**			
mbl	FBgn0265487	**2.53**			
CG2681	FBgn0024997	**2.68**			
Irbp	FBgn0011774	**2.71**	**14.23**		
CG3328	FBgn0034985	**2.75**			
GluRIIC	FBgn0046113	**2.75**			
phr	FBgn0003082	**2.79**			
Obp56a	FBgn0034468	**2.79**			
CG5205	FBgn0038344	**2.89**	**2.44**		
CG44085	FBgn0264894	**2.91**			
Diver		**3.03**	**3.62**		
CG30118	FBgn0050118	**3.03**	**2.00**		
CG6503	FBgn0040606	**3.05**	**10.18**		
ng3	FBgn0010295	**3.05**			
zormin	FBgn0052311	**3.34**	**0.456**		
ps	FBgn0261552	**3.34**			
Skeletor	FBgn0262717	**3.48**			
CG14661	FBgn0037288	**3.68**			
CG6910	FBgn0036262	**3.73**	**2.65**		
CG12863	FBgn0033948	**3.78**			
CG30285	FBgn0050285	**4.35**	**4.04**		
CG44013	FBgn0264775	**4.89**			
Ude	FBgn0039226	**5.03**			**3.85**
CG2120	FBgn0030005	**5.28**			
CG44013	FBgn0264775	**5.46**			
tobi	FBgn0261575	**5.94**			
CG6839	FBgn0036831	**6.06**			
CG7299	FBgn0032282	**6.63**			
CG11659	FBgn0038731	**7.21**			

CG5070	FBgn0030824	**8.69**			
CG9757	FBgn0003060	**16.8**			
**metabolism**					
CG30427	FBgn0043792	**2.00**			
CG7149	FBgn0031948	**2.00**			
Evi5	FBgn0262740	**2.01**			
CG3902	FBgn0036824	**2.03**			
CG11453	FBgn0038734	**2.04**			
CG15534	FBgn0039769	**2.11**			
Marf	FBgn0029870	**2.11**			
CG40486	FBgn0263830	**2.13**			
Unc-115b	FBgn0260463	**2.14**			
CG9674	FBgn0036663	**2.16**			
CG3523	FBgn0027571	**2.16**	**2.37**		
CG6733	FBgn0039052	**2.16**			
su(r)	FBgn0086450	**2.17**	**3.20**	**2.45**	
Men	FBgn0002719	**2.20**	**4.11**		
Amy-p	FBgn0000079	**2.20**			
alpha-Est10	FBgn0015569	**2.22**	**3.40**		
CG43340	FBgn0263077	**2.22**		**3.00**	
CG32645	FBgn0052645	**2.22**			
pgant4	FBgn0051956	**2.22**			
CG17322	FBgn0027070	**2.22**			
CG31475	FBgn0051475	**2.23**			
AdSS	FBgn0027493	**2.23**	**2.01**		
GstZ2	FBgn0037697	**2.25**			
nemy	FBgn0261673	**2.27**			
Ugt86Da	FBgn0040259	**2.28**	**8.97**	**2.71**	
CAHbeta	FBgn0037646	**2.30**			
CG7920	FBgn0039737	**2.30**			
Pepck	FBgn0003067	**2.30**		**4.99**	**2.84**
Spat	FBgn0014031	**2.31**	**2.38**		
Mocs1	FBgn0263241	**2.31**			
CG32649	FBgn0052649	**2.31**			
Pkc53E	FBgn0003091	**2.33**	**0.340**		
CG14655	FBgn0037275	**2.33**			
CG13397	FBgn0014417	**2.33**			
CG8129	FBgn0037684	**2.35**	**2.00**		
Ac78C	FBgn0024150	**2.38**			
Kua	FBgn0032850	**2.38**			
Ca-P60A	FBgn0263006	**2.43**			
CG11162	FBgn0030509	**2.43**			
CG32557	FBgn0052557	**2.46**			
Uro	FBgn0003961	**2.48**	**2.13**		
CG3597	FBgn0031417	**2.48**	**2.08**		
CG3301	FBgn0038878	**2.48**	**0.448**		
Aldh	FBgn0012036	**2.48**	**2.56**		
CG5704	FBgn0026570	**2.50**	**2.10**		
CG1315	FBgn0026565	**2.51**			
Ugt35a	FBgn0026315	**2.53**	**3.35**	**2.47**	
drd	FBgn0260006	**2.55**			
ACC	FBgn0033246	**2.57**	**7.19**		

Tdc1	FBgn0259977	**2.57**			
CG1673	FBgn0030482	**2.58**			
AOX1	FBgn0267408	**2.60**			
Gpdh	FBgn0001128	**2.62**	**2.22**		
CG1774	FBgn0039856	**2.64**			
CG10175	FBgn0039084	**2.66**	**0.481**		
Jheh3	FBgn0034406	**2.68**		**6.01**	
CG10827	FBgn0038845	**2.68**			
Gad1	FBgn0004516	**2.73**			
Est-6	FBgn0000592	**2.77**	**7.32**		
CG33093	FBgn0053093	**2.79**	**7.39**		
CG6074	FBgn0039486	**2.79**	**23.77**		
Tie	FBgn0014073	**2.81**	**0.205**		
CG8128	FBgn0030668	**2.83**			
alpha-Est8	FBgn0015576	**2.83**			**0.374**
CG5707	FBgn0026593	**2.87**			
CG13311	FBgn0035929	**2.89**			
Obp56e	FBgn0034471	**2.89**			
l(1)G0196	FBgn0027279	**2.89**			
CG14787	FBgn0027793	**2.91**			**17.33**
CG3841	FBgn0032131	**2.95**			
CG9993	FBgn0034553	**2.97**			
sls	FBgn0086906	**3.10**	**0.081**		
CG30503	FBgn0050503	**3.14**	**0.392**		**2.60**
CG14762	FBgn0033250	**3.16**	**33.48**		
CG10165	FBgn0032801	**3.16**	**3.66**		
Gbs-76A	FBgn0036862	**3.18**			
Ugt86Dh	FBgn0040252	**3.29**			
CG7025	FBgn0031930	**3.29**			
Cyp18a1	FBgn0010383	**3.32**			
Npc2h	FBgn0039801	**3.32**	**5.23**		
Gpo-1	FBgn0022160	**3.32**	**3.37**		**0.493**
RluA-1	FBgn0051719	**3.34**			
Ork1	FBgn0017561	**3.36**	**11.63**		
Mipp1	FBgn0026061	**3.36**	**4.33**		
CG15879	FBgn0035309	**3.39**			
CG42329	FBgn0259229	**3.41**	**3.51**		
CG9466	FBgn0032068	**3.48**			
CG8708	FBgn0033271	**3.58**	**3.21**		
CG3835	FBgn0023507	**3.58**	**3.69**		
sls	FBgn0086906	**3.63**	**0.081**		
fa2h	FBgn0050502	**3.66**			
CG1461	FBgn0030558	**3.66**			
CG8629	FBgn0035742	**3.89**			
CG10592	FBgn0035619	**3.97**			
CG15117	FBgn0034417	**4.06**			
CG32170	FBgn0052170	**4.06**			
Unc-89	FBgn0053519	**4.06**			
St3	FBgn0265052	**4.08**			
Gpdh	FBgn0001128	**4.29**	**2.22**		
Mlc2	FBgn0002773	**4.29**	**2.31**		
arg	FBgn0023535	**4.35**	**5.01**		

CS-2	FBgn0029091	**4.50**			
CG17843	FBgn0038919	**4.50**			
AOX2	FBgn0038348	**4.69**			
glob1	FBgn0027657	**4.72**	**6.42**		
CG30457	FBgn0050457	**4.79**			
CG9743	FBgn0039756	**5.10**	**2.17**		
CG32751	FBgn0052751	**5.13**			
CG9468	FBgn0032069	**5.13**			
CG4753	FBgn0036622	**5.13**	**8.81**		
CG42249	FBgn0259101	**5.28**			
CG12512	FBgn0031703	**5.31**	**11.53**		
Apoltp	FBgn0032136	**5.70**	**2.89**	**2.39**	**2.22**
CG5150	FBgn0035620	**6.28**			
vanin-like	FBgn0040069	**7.57**			
CG8630	FBgn0038130	**8.22**			
CG3106	FBgn0030148	**8.63**			
CG14022	FBgn0031700	**8.69**			
Odc2	FBgn0013308	**9.45**			
CG14205	FBgn0031034	**13.4**			
frac	FBgn0035798	**20.3**			
Jhedup	FBgn0034076	**21.3**			
CG11796	FBgn0036992	**22.9**			
hgo	FBgn0040211	**23.4**			
CG4716	FBgn0033820	**32.0**	**11.77**		
CG4716	FBgn0033820	**61.8**	**11.77**		
CG4757	FBgn0027584	**190**	**5.27**		
**proteolysis**					
CG9505	FBgn0031805	**2.00**	**2.72**		
CG18417	FBgn0035780	**2.01**			
CG31821	FBgn0051821	**2.08**			
Try29F	FBgn0015316	**2.10**	**2.28**		
CG18493	FBgn0038701	**2.14**			
CG4721	FBgn0039024	**2.14**	**2.82**		
CG17739	FBgn0033710	**2.14**			
CG4725	FBgn0039022	**2.20**			
Kaz-m1	FBgn0002578	**2.30**			
l(2)34Fc	FBgn0261534	**2.33**			
scaf	FBgn0033033	**2.33**			
CG6225	FBgn0038072	**2.38**			
CG16712	FBgn0031561	**2.48**	**7.20**	**2.68**	
Bace	FBgn0032049	**2.48**			
iotaTry	FBgn0015001	**2.50**			
stv	FBgn0086708	**2.53**		**4.30**	
CG17477	FBgn0038479	**2.58**			
CG30043	FBgn0050043	**2.66**			
CG6337	FBgn0033873	**2.71**			
CG32483	FBgn0052483	**2.77**			**7.18**
CG6048	FBgn0029827	**2.87**			
CG5246	FBgn0038484	**2.91**			
CG3604	FBgn0031562	**2.91**	**3.70**		
zetaTry	FBgn0011556	**2.99**			
Nep1	FBgn0029843	**3.03**			

CG4053	FBgn0038482	**3.10**			
CG10472	FBgn0035670	**3.16**			
CG11961	FBgn0034436	**3.25**			
CG17475	FBgn0038481	**3.25**			
CG13160	FBgn0033720	**3.41**			
CG17134	FBgn0032304	**3.43**			
CG10051	FBgn0034437	**3.81**			
CG33127	FBgn0053127	**3.84**			
CG33459	FBgn0053459	**3.86**	**10.43**		
CG30371	FBgn0050371	**4.08**	**0.160**		
CG31233	FBgn0051233	**4.14**			
kappaTry	FBgn0043471	**4.38**			
CG4653	FBgn0030776	**4.41**			
CG31343	FBgn0051343	**4.41**	**12.63**		
lambdaTry	FBgn0043470	**4.47**			
Sp212	FBgn0053329	**4.82**			
CG1304	FBgn0031141	**5.35**			
CG3513	FBgn0031559	**5.35**	**11.02**		
CG31198	FBgn0051198	**5.58**			
CG8774	FBgn0038136	**7.73**			
Spn47C	FBgn0033574	**7.73**			
CG14820	FBgn0035718	**8.00**			
CG31265	FBgn0051265	**9.06**			
CG15254	FBgn0028949	**14.7**			
**signaling**					
PKD	FBgn0038603	**2.04**		**4.00**	
moody	FBgn0025631	**2.06**	**2.28**		
Syn	FBgn0004575	**2.07**	**2.44**		
Fas2	FBgn0000635	**2.11**		**0.479**	
mthl6	FBgn0035789	**2.14**			
CrzR	FBgn0036278	**2.16**			
Ag5r	FBgn0015010	**2.19**	**6.17**		
Tk	FBgn0037976	**2.22**			
Galphaf	FBgn0010223	**2.30**	**6.31**		
Amph	FBgn0027356	**2.35**			
Dh31-R	FBgn0052843	**2.50**			
CG12290	FBgn0039419	**2.57**			
CG7054	FBgn0038972	**2.66**	**3.91**		
Pkcdelta	FBgn0259680	**2.71**			
CG15529	FBgn0039748	**2.83**			
Mctp	FBgn0034389	**2.95**	**0.300**	**2.11**	**2.25**
Pde6	FBgn0038237	**2.95**	**5.21**		
CG5402	FBgn0039521	**3.39**			
Ac76E	FBgn0004852	**3.43**			
CG8907	FBgn0038466	**3.48**	**2.81**		
CG9498	FBgn0031801	**4.56**	**3.62**		
RyR	FBgn0011286	**8.88**			
**tissue structure**					
CAP	FBgn0033504	**2.03**	**2.32**		
Ag5r2	FBgn0020508	**2.03**			
Cpr97Eb	FBgn0039481	**2.04**			
LamC	FBgn0010397	**2.08**			**2.95**

CG8927	FBgn0038405	**2.08**			
Idgf5	FBgn0064237	**2.11**	**2.30**		
Lcp65Af	FBgn0020639	**2.14**			
CG14304	FBgn0038629	**2.16**			
CG7298	FBgn0036948	**2.17**			
kst	FBgn0004167	**2.19**			
Acp1	FBgn0014454	**2.25**			
Cht7	FBgn0035398	**2.27**	**0.194**		
verm	FBgn0261341	**2.28**			
Cpr11B	FBgn0030398	**2.30**			
Cda5	FBgn0051973	**2.33**	**0.109**		
obst-I	FBgn0052304	**2.33**			
Cht6	FBgn0263132	**2.38**			
CG4367	FBgn0038783	**2.39**			
obst-H	FBgn0053983	**2.39**	**11.78**		
Cpr67Fa1	FBgn0036108	**2.41**			
Gasp	FBgn0026077	**2.43**			
Cpr67Fa2	FBgn0036109	**2.45**			
CG7252	FBgn0036226	**2.46**			
CG33468	FBgn0053468	**2.46**	**4.91**	**2.57**	
nrm	FBgn0262509	**2.48**			
Lcp65Ag3	FBgn0086611	**2.50**			
Lcp65Ag2	FBgn0020637	**2.51**			
Muc11A	FBgn0052656	**2.68**			
CG32284	FBgn0052284	**2.69**			
obst-F	FBgn0036947	**2.77**			
Mur18B	FBgn0030999	**2.79**			
CG43896	FBgn0264488	**2.85**			
CG43896	FBgn0264488	**2.91**			
Cpr62Bc	FBgn0035281	**2.95**			
CG5883	FBgn0036225	**2.95**			
PH4alphaSG1	FBgn0051014	**3.07**			
Muc96D	FBgn0051439	**3.18**			
PH4alphaPV	FBgn0051015	**3.27**			
Tg	FBgn0031975	**3.41**	**0.241**		
Muc18B	FBgn0031000	**3.63**	**2.48**		
Cpr49Ah	FBgn0033731	**3.86**			
Cpr11A	FBgn0030394	**3.97**			
obst-E	FBgn0031737	**3.97**			
CG10725	FBgn0036362	**4.20**			
TwdlO	FBgn0039438	**4.35**			
Mal-A3	FBgn0002571	**4.47**			
ng2	FBgn0010294	**5.17**			
Cpr49Af	FBgn0033729	**5.21**			
ng1	FBgn0002933	**5.94**			
CG10154	FBgn0036361	**6.68**			
Muc55B	FBgn0034294	**6.96**			
CG15515	FBgn0039719	**7.16**			
CG4835	FBgn0035607	**10.1**			
Cpr47Eb	FBgn0033598	**14.1**	**25.21**		
CG12491	FBgn0034900	**31.8**	**6.23**		
**transport**					

CG16989	FBgn0025621	**2.03**			
Oatp74D	FBgn0036732	**2.04**	**2.37**		
Prestin	FBgn0036770	**2.06**	**2.22**		
CG7458	FBgn0037144	**2.06**			**0.413**
CG11147	FBgn0031734	**2.10**			
CG1718	FBgn0031170	**2.13**	**2.30**		
CG9702	FBgn0039787	**2.13**			
CG11897	FBgn0039644	**2.14**	**5.52**	**2.61**	**2.59**
CG5326	FBgn0038983	**2.16**	**2.44**		
CG7084	FBgn0038938	**2.16**			
MRP	FBgn0032456	**2.16**	**2.08**		
CG6901	FBgn0038414	**2.17**			
CG8028	FBgn0031010	**2.17**			
CG9413	FBgn0030574	**2.20**			
Mdr65	FBgn0004513	**2.27**			
ppk6	FBgn0034489	**2.39**			
p24-2	FBgn0053105	**2.39**	**0.122**		
CG31792	FBgn0051792	**2.43**			
Vha100-4	FBgn0038613	**2.43**			
CG30016	FBgn0050016	**2.50**			
hoe1	FBgn0041150	**2.53**			
Tsf1	FBgn0022355	**2.57**	**7.68**		
CG31743	FBgn0032618	**2.60**			**4.18**
path	FBgn0036007	**2.60**			
AQP	FBgn0033807	**2.64**			
Smvt	FBgn0039873	**2.68**			
Tsf1	FBgn0022355	**2.69**	**7.68**		
CG10505	FBgn0034612	**2.71**			
Oatp58Dc	FBgn0034716	**2.73**			
CG9981	FBgn0030746	**2.77**		**9.00**	
Calx	FBgn0013995	**2.81**			
CG13124	FBgn0032156	**2.91**			
Rab9	FBgn0032782	**2.93**	**2.56**		
CG17636	FBgn0025837	**2.97**			
CG33281	FBgn0053281	**3.01**			
Rh50	FBgn0028699	**3.01**			
CG30272	FBgn0050272	**3.05**			
Oatp33Eb	FBgn0032435	**3.14**			
CG7912	FBgn0039736	**3.16**			
CG8791	FBgn0033234	**3.20**	**13.03**		
salt	FBgn0039872	**3.27**			
CG8051	FBgn0031012	**4.03**			
CG2187	FBgn0017448	**4.06**	**3.10**		
st	FBgn0003515	**4.06**			
CG4830	FBgn0037996	**4.35**			
CG6293	FBgn0037807	**4.99**	**3.59**		
CG9780	FBgn0037230	**5.13**	**17.37**		
CG18327	FBgn0033904	**5.66**			
Nha2	FBgn0263390	**6.11**		**2.32**	
CG4991	FBgn0030817	**6.82**	**3.76**		
CG9825	FBgn0034783	**6.96**			
CG8654	FBgn0034479	**9.85**			

**unknown function**					
CG9682	FBgn0039760	**2.00**			
CG11158	FBgn0030511	**2.01**	**2.73**		
Ilp8	FBgn0036690	**2.01**			
CG17147	FBgn0260393	**2.01**			
CG7992	FBgn0031004	**2.03**			
CG15597	FBgn0037420	**2.03**			
CG13360	FBgn0025620	**2.04**			
CG15236	FBgn0033108	**2.04**			
Piezo	FBgn0264953	**2.04**			
Rcd2	FBgn0037012	**2.04**	**6.47**		
CG32485	FBgn0052485	**2.04**			
CG11168	FBgn0039249	**2.06**			
jp	FBgn0032129	**2.06**	**0.303**		
CG15523	FBgn0039727	**2.06**	**0.328**		
CG5399	FBgn0038353	**2.06**	**28.01**	**2.91**	
CG5819	FBgn0034717	**2.07**			
CG15043	FBgn0030929	**2.07**			
CG8177	FBgn0036043	**2.08**	**0.177**		
CG8087	FBgn0038241	**2.08**	**0.445**		
CG31321	FBgn0051321	**2.10**			
CG12075	FBgn0030065	**2.10**			
CG7920	FBgn0039737	**2.10**			
CG14907	FBgn0038455	**2.10**	**6.93**		
Tyler	FBgn0031038	**2.10**	**2.09**		
CG12826	FBgn0033207	**2.10**	**6.33**		
CG5506	FBgn0036766	**2.10**			
LP09564	FBcl0187291	**2.10**			
Pebp1	FBgn0038973	**2.11**			
CG32714	FBgn0260483	**2.13**			
CG10936	FBgn0034253	**2.14**	**9.27**		
CG13806	FBgn0035325	**2.14**			
Rootletin	FBgn0039152	**2.16**			
CG5084	FBgn0034288	**2.16**			
GM03661	FBcl0137693	**2.16**			
CG14456	FBgn0037176	**2.17**			
atilla	FBgn0032422	**2.17**			
CG2010	FBgn0039667	**2.20**			
Pdxk	FBgn0085484	**2.22**			
CG5767	FBgn0034292	**2.22**			
CG8852	FBgn0031548	**2.23**			
CG31974	FBgn0051974	**2.23**	**4.74**		**2.12**
CG40198	FBgn0058198	**2.23**			
CG12177	FBgn0030510	**2.25**	**6.26**		
CG43693	FBgn0263776	**2.27**			
CG13912	FBgn0035186	**2.27**		**2.23**	
CG13003	FBgn0030798	**2.28**			
Ect4	FBgn0262579	**2.28**		**2.17**	
CG11313	FBgn0039798	**2.30**	**5.46**		
CG14273	FBgn0032024	**2.30**			
CG32603	FBgn0052603	**2.30**			
CG5866	FBgn0038508	**2.31**			

TwdlBeta	FBgn0033658	**2.31**			
CG31769	FBgn0051769	**2.33**	**2.49**		**0.380**
CG4733	FBgn0038744	**2.33**			
CG34120	FBgn0083956	**2.35**			
CG6023	FBgn0030912	**2.35**	**2.88**		
CG43078	FBgn0262508	**2.35**			
CG2157	FBgn0030244	**2.35**			
CG9399	FBgn0037715	**2.36**	**2.74**		
CG30411	FBgn0050411	**2.36**	**0.040**		
CG9568	FBgn0032087	**2.38**			
CG31446	FBgn0051446	**2.38**			
CG18635	FBgn0034279	**2.38**			
GIIIspla2	FBgn0030013	**2.39**			
CG1698	FBgn0033443	**2.39**			
CG42249	FBgn0259101	**2.41**			
CG15199	FBgn0030270	**2.41**	**3.28**		
CG31086	FBgn0051086	**2.41**			
CG9626	FBgn0037565	**2.46**			
CG31259	FBgn0051259	**2.48**			
CG17108	FBgn0032285	**2.48**			
CG14893	FBgn0038451	**2.50**			
CG5535	FBgn0036764	**2.51**	**7.41**		
CG31269	FBgn0051269	**2.57**			
CG33469	FBgn0053469	**2.58**			
CG13323	FBgn0033788	**2.58**			
CG16820	FBgn0032495	**2.60**			
CR14033	FBgn0046776	**2.60**	**41.33**		
CG31464	FBgn0051464	**2.64**			
HDC07637		**2.64**			
ppk13	FBgn0053508	**2.66**			
SP1173	FBgn0035710	**2.71**			
ps	FBgn0261552	**2.71**			
CG7900	FBgn0037548	**2.71**	**2.96**		**0.476**
Lmpt	FBgn0261565	**2.73**			
CG31288	FBgn0051288	**2.75**	**28.68**		
CG17167	FBgn0039941	**2.75**			
CG12105	FBgn0035241	**2.75**			
Skeletor	FBgn0262717	**2.75**			
CG44250	FBgn0265185	**2.75**			
CG13492	FBgn0034662	**2.77**			
l(1)G0196	FBgn0027279	**2.79**			
HDC03517		**2.79**			
CG43897	FBgn0264489	**2.79**			
CT22789		**2.81**			
mtg	FBgn0260386	**2.83**			**2.11**
CR31781	FBgn0051781	**2.85**	**0.354**		
CG15412	FBgn0031528	**2.85**			
CG11160	FBgn0030257	**2.87**			
CG10597	FBgn0030832	**2.87**			
CG15741	FBgn0030338	**2.91**	**76.88**		
CG10732	FBgn0036365	**2.93**			
CG7631	FBgn0028945	**2.99**			

CG32198	FBgn0052198	**3.12**			
CG15213	FBgn0040843	**3.12**			
CG7300	FBgn0032286	**3.16**			
CG43427	FBgn0263346	**3.20**			
CG14852	FBgn0038242	**3.20**	**0.084**		
CG34278	FBgn0085307	**3.25**	**4.76**		
CG13102	FBgn0032088	**3.32**			
Cpr47Ec	FBgn0033600	**3.32**			
Obp83g	FBgn0046875	**3.32**			
CR31781	FBgn0051781	**3.34**	**0.354**		
GM10545	FBcl0137569	**3.34**			
HDC03535		**3.36**			
CG15530	FBgn0039752	**3.41**			
CG9396	FBgn0037714	**3.53**	**5.56**		
CT37020		**3.56**			
GstD7	FBgn0010043	**3.61**	**3.98**		**12.65**
CG13705	FBgn0035582	**3.78**			
CG13946	FBgn0040725	**3.92**	**30.28**		
CG4962	FBgn0036597	**4.03**			
CG14292	FBgn0038658	**4.11**			
CG42565	FBgn0260767	**4.47**			
CG32241	FBgn0052241	**4.72**			
CG5810	FBgn0038866	**4.79**			
CG13460	FBgn0036471	**4.79**			
CG18649	FBgn0036469	**5.06**			
CG11350	FBgn0035552	**5.28**			
CG8773	FBgn0038135	**5.35**			
CG43078	FBgn0262508	**5.58**			
CG2064	FBgn0033205	**5.86**	**2.24**		
CG14120	FBgn0036321	**6.02**			
CG14879	FBgn0038419	**6.15**			
CG32073	FBgn0052073	**6.19**	**19.44**		
CG10953	FBgn0034204	**6.23**			
CG9555	FBgn0032085	**8.00**			
Cyp4p3	FBgn0033397	**8.75**	**9.73**	**2.75**	
CG12506	FBgn0031276	**9.92**	**18.91**		
CG32564	FBgn0052564	**14.93**	**3.08**		
CG8534	FBgn0037761	**3.10**			
CG13640	FBgn0039237	**6.23**			
Chd1* effect: *DOWN					
**detoxification**					
Cyp312a1	FBgn0036778	**0.497**			
Cyp6d5	FBgn0038194	**0.490**		**3.00**	**13.46**
Cyp309a1	FBgn0031432	**0.444**	**2.62**		
GstE3	FBgn0063497	**0.432**		**5.89**	
GstD10	FBgn0042206	**0.409**			
Hsp22	FBgn0001223	**0.342**	**2.94**		
GstT3	FBgn0031117	**0.337**	**2.41**		
CG6762	FBgn0030876	**0.316**			
Hsp26	FBgn0001225	**0.304**			
GstE1	FBgn0034335	**0.304**	**2.21**		
MtnD	FBgn0053192	**0.287**	**12.01**		

CG17323	FBgn0032713	**0.272**			
MtnC	FBgn0038790	**0.248**			
GstD2	FBgn0010038	**0.235**	**9.61**	**2.74**	
GstD4	FBgn0010040	**0.216**			
Hsp67Bc	FBgn0001229	**0.200**	**2.74**	**2.38**	
NLaz	FBgn0053126	**0.171**			
CG7227	FBgn0031970	**0.143**	**0.310**		
MtnB	FBgn0002869	**0.092**			
**development**					
yellow-d2	FBgn0034856	**0.497**			
sad	FBgn0003312	**0.493**			
rdo	FBgn0243486	**0.473**	**0.207**		
ImpE1	FBgn0001253	**0.435**			
in	FBgn0001259	**0.432**			
neo	FBgn0039704	**0.409**			
CG15153	FBgn0032663	**0.361**			
CG18607	FBgn0034429	**0.344**			
nvd	FBgn0259697	**0.312**			
CG9005	FBgn0033638	**0.272**	**0.260**		
ImpE1	FBgn0001253	**0.264**			
CG2016	FBgn0250839	**0.188**			
Eig71Ed	FBgn0004591	**0.184**	**0.127**		
Yp3	FBgn0004047	**0.180**		**16.14**	
Eip74EF	FBgn0000567	**0.176**	**0.341**		
ImpE3	FBgn0001255	**0.171**			**2.10**
Eip78C	FBgn0004865	**0.144**	**0.136**	**2.12**	
ImpE2	FBgn0001254	**0.132**			
Eig71Ea	FBgn0004588	**0.126**	**0.037**		
Eig71Ec	FBgn0004590	**0.115**			
slbo	FBgn0005638	**0.115**	**5.57**		
Eig71Eg	FBgn0004594	**0.103**	**0.027**		
Eig71Eb	FBgn0004589	**0.023**	**0.403**		
**immune response**					
sn	FBgn0003447	**0.476**			
Hsp27	FBgn0001226	**0.457**			**2.41**
Drs	FBgn0010381	**0.451**	**4.28**	**2.53**	**3.78**
Mtk	FBgn0014865	**0.438**		**4.93**	**6.09**
CG8157	FBgn0034010	**0.374**	**3.95**		
CG4091	FBgn0034894	**0.354**			
LysX	FBgn0004431	**0.344**	**33.21**	**2.92**	
AttA	FBgn0012042	**0.337**		**7.68**	
E(spl)malpha-BFM	FBgn0002732	**0.334**	**3.57**		
spz3	FBgn0031959	**0.332**			
Tsp42Ep	FBgn0033137	**0.332**	**0.395**		
Atg7	FBgn0034366	**0.306**			
dsb	FBgn0035290	**0.301**	**0.423**		
E(spl)m2-BFM	FBgn0002592	**0.291**			
CG9616	FBgn0038214	**0.289**			
NimC2	FBgn0028939	**0.287**	**0.140**		
spirit	FBgn0030051	**0.266**			
Mmp1	FBgn0035049	**0.264**	**15.55**	**2.88**	**3.34**
wbl	FBgn0004003	**0.261**	**0.495**		

Npc2e	FBgn0051410	**0.233**	**0.028**		
PGRP-SC2	FBgn0043575	**0.222**	**2.66**	**2.37**	
santa-maria	FBgn0025697	**0.218**	**0.036**		
IM2	FBgn0025583	**0.218**			
CG11425	FBgn0037167	**0.183**			
CG2065	FBgn0033204	**0.163**	**9.00**	**2.72**	
CG3397	FBgn0037975	**0.162**		**3.78**	
Spn43Aa	FBgn0024294	**0.160**	**0.336**		
CG6429	FBgn0046999	**0.147**			
IM3	FBgn0040736	**0.139**	**11.65**		
PGRP-SB1	FBgn0043578	**0.108**		**3.12**	
IM1	FBgn0034329	**0.104**	**33.02**		
Drsl5	FBgn0035434	**0.092**			
Spn100A	FBgn0039795	**0.067**			
CG13606	FBgn0039161	**0.042**			
PGRP-SB2	FBgn0043577	**0.023**	**12.29**		
Drsl2	FBgn0052279	**0.020**			
**intracellular processes**					
CG5745	FBgn0038855	**0.497**			
CG17754	FBgn0030114	**0.490**	**2.66**		**2.07**
insc	FBgn0011674	**0.490**			
CG8420	FBgn0037664	**0.490**			
GV1	FBgn0027790	**0.486**			
RpL22-like	FBgn0034837	**0.483**			
RpS19b	FBgn0039129	**0.483**			
His2B:CG17949	FBgn0061209	**0.476**			
CG4496	FBgn0031894	**0.476**	**0.293**		
lbm	FBgn0016032	**0.476**			
nsr	FBgn0034740	**0.476**			
Eip71CD	FBgn0000565	**0.473**			
Drep-3	FBgn0028407	**0.470**			
CG32971	FBgn0052971	**0.470**			
tbrd-1	FBgn0039124	**0.470**			
Scr	FBgn0003339	**0.467**			
sc	FBgn0004170	**0.460**			
MED9	FBgn0260401	**0.457**			
CG30431	FBgn0050431	**0.457**			
en	FBgn0000577	**0.454**			
CG17450	FBgn0040028	**0.454**			
CG3726	FBgn0029824	**0.448**			
thoc6	FBgn0036263	**0.448**			
RpL37b	FBgn0034822	**0.444**			
Lmpt	FBgn0261565	**0.444**			
CG31642	FBgn0051642	**0.435**		**79.80**	
Hsp67Ba	FBgn0001227	**0.429**	**3.25**		
Dark	FBgn0263864	**0.418**			**2.02**
CG31274	FBgn0051274	**0.418**	**0.342**		**5.24**
betaNACtes4	FBgn0030566	**0.415**			
CG6891	FBgn0030955	**0.412**			
CG12493	FBgn0035571	**0.406**	**117.75**		
GV1	FBgn0027790	**0.398**			
CG18446	FBgn0033458	**0.395**	**2.05**	**2.27**	

CG8335	FBgn0033069	**0.392**			
His1:CG31617	FBgn0051617	**0.387**			
Eip93F	FBgn0264490	**0.384**			
CR11386	FBgn0260447	**0.382**			
toy	FBgn0019650	**0.379**	**24.16**		
E(spl)m7-HLH	FBgn0002633	**0.376**			
CG4080	FBgn0035983	**0.369**	**0.405**		
Taf12L	FBgn0031623	**0.369**	**191.54**		
betaNACtes3	FBgn0052601	**0.369**			
CG12477	FBgn0036809	**0.356**			
CG8679	FBgn0032934	**0.344**			
E(spl)m8-HLH	FBgn0000591	**0.337**			
toy	FBgn0019650	**0.332**	**24.16**		
His2A:CG31618	FBgn0051618	**0.330**			
blanks	FBgn0035608	**0.321**	**98.58**		**0.453**
CG4021	FBgn0034659	**0.281**			
CG7804	FBgn0036496	**0.270**			
CG9989	FBgn0039593	**0.261**		**8.99**	
CG34434	FBgn0250904	**0.200**			
CG17386	FBgn0033936	**0.087**			
Chd1	FBgn0250786	**0.013**			
**metabolism**					
CAH1	FBgn0027844	**0.493**	**2.25**	**2.30**	**2.11**
Ho	FBgn0037933	**0.493**			
CG1637	FBgn0030245	**0.486**			
CG1941	FBgn0033214	**0.486**			
CG7742	FBgn0031690	**0.486**			
CG3376	FBgn0034997	**0.486**	**0.330**		
CLS	FBgn0039360	**0.476**			
wat	FBgn0039620	**0.470**			
CG5554	FBgn0034914	**0.463**	**0.379**		
Sep5	FBgn0026361	**0.454**	**2.36**		
Scp2	FBgn0020907	**0.451**			
Dyrk2	FBgn0016930	**0.448**		**2.13**	
ade3	FBgn0000053	**0.444**	**0.423**		
CG5854	FBgn0039130	**0.441**	**0.477**		
CG1942	FBgn0033215	**0.426**			
Oscillin	FBgn0031717	**0.426**	**0.302**		
CG15343	FBgn0030029	**0.423**	**0.124**		**3.07**
CG18528	FBgn0039189	**0.423**			
CG4546	FBgn0038373	**0.418**			
CG9509	FBgn0030594	**0.415**	**0.147**		
CG8565	FBgn0030697	**0.406**			
CG13833	FBgn0039040	**0.398**			
CG8112	FBgn0037612	**0.395**	**0.205**		
CG3264	FBgn0034712	**0.392**			
Dhpr	FBgn0035964	**0.387**			
CG6287	FBgn0032350	**0.382**		**2.03**	
CG11170	FBgn0034705	**0.379**	**0.365**		
slow	FBgn0035539	**0.376**	**2.34**		
CG4586	FBgn0029924	**0.376**	**0.432**		
CG17191	FBgn0039473	**0.376**			

CG12539	FBgn0030586	**0.374**			
MESR6	FBgn0036846	**0.361**	**0.364**		
CG33096	FBgn0053096	**0.361**			
yellow-f	FBgn0041710	**0.358**	**0.330**	**2.09**	
Pu	FBgn0003162	**0.356**			
CG3940	FBgn0037788	**0.356**		**8.33**	
CG7320	FBgn0036782	**0.349**			
Est-P	FBgn0000594	**0.342**			
CG8100	FBgn0036410	**0.339**			
Ady43A	FBgn0026602	**0.339**			
Nmdmc	FBgn0010222	**0.330**			
CG14946	FBgn0032405	**0.310**			
Asph	FBgn0034075	**0.308**	**0.427**		**2.25**
Oat	FBgn0022774	**0.306**	**0.077**		**3.00**
AANATL2	FBgn0031791	**0.295**	**0.305**		
CG4842	FBgn0036620	**0.289**			
spok	FBgn0086917	**0.289**			
Ddc	FBgn0000422	**0.259**			
CG5618	FBgn0036975	**0.252**			
Acp63F	FBgn0015585	**0.237**			
CG10178	FBgn0032684	**0.178**			
CG5171	FBgn0031907	**0.149**		**20.62**	
yellow-c	FBgn0041713	**0.146**			**4.92**
CG4382	FBgn0032132	**0.131**			
CG8093	FBgn0033999	**0.113**			
CG6277	FBgn0039475	**0.107**			
w	FBgn0003996	**0.079**	**4.39**		
CG9452	FBgn0036877	**0.073**			
CG18606	FBgn0034428	**0.069**			
CG14406	FBgn0030595	**0.061**	**0.314**		
CG2070	FBgn0033203	**0.047**			
St2	FBgn0037665	**0.045**			
e	FBgn0000527	**0.042**	**2.93**		**5.46**
**proteolysis**					
hh	FBgn0004644	**0.497**			
CG31778	FBgn0051778	**0.483**			
S-Lap3	FBgn0045770	**0.457**			
Rpt6R	FBgn0039788	**0.451**	**36.11**		
Spn88Eb	FBgn0038299	**0.451**	**6.72**		
S-Lap2	FBgn0052351	**0.451**			
CG8586	FBgn0033320	**0.438**		**6.89**	
Sb	FBgn0003319	**0.432**			
Spn42Dd	FBgn0028988	**0.426**	**0.341**		
CG11023	FBgn0031208	**0.420**			
Sp7	FBgn0037515	**0.401**			**0.466**
CG10764	FBgn0034221	**0.390**			**2.61**
Jon99Ci	FBgn0003358	**0.376**	**2.55**		
CG4408	FBgn0039073	**0.374**	**0.260**		
CG8550	FBgn0033742	**0.361**			
CG30289	FBgn0050289	**0.354**	**0.151**		
CG9850	FBgn0034903	**0.334**			
CG31777	FBgn0051777	**0.323**	**2.75**		

CG4914	FBgn0036436	**0.323**			
CG30288	FBgn0050288	**0.312**	**0.407**		
CG9850	FBgn0034903	**0.304**			
Nep2	FBgn0027570	**0.283**		**4.58**	
NnaD	FBgn0265726	**0.266**			
CG11529	FBgn0036264	**0.255**			
CG3355	FBgn0031619	**0.252**			
CG30098	FBgn0050098	**0.250**			
SP1029	FBgn0263236	**0.200**			
CG5470	FBgn0038384	**0.199**			
CG30091	FBgn0050091	**0.187**			**11.62**
CG4650	FBgn0032549	**0.173**	**3.36**		
CG1773	FBgn0033439	**0.168**	**0.148**		
CG3700	FBgn0034796	**0.157**			
CG10073	FBgn0034440	**0.154**			
CG3502	FBgn0046253	**0.153**			
CG3097	FBgn0029804	**0.149**			
CG7906	FBgn0036417	**0.139**			
CG13748	FBgn0033355	**0.133**			
CG7924	FBgn0036416	**0.076**			
CG32762	FBgn0052762	**0.067**			
CG11459	FBgn0037396	**0.062**			
CG12951	FBgn0037677	**0.062**			
**signaling**					
bib	FBgn0000180	**0.493**			
SoxN	FBgn0029123	**0.483**			
mthl5	FBgn0037960	**0.476**			**2.17**
E(spl)m4-BFM	FBgn0002629	**0.470**			
CG17760	FBgn0033756	**0.412**	**3.33**		
Takl1	FBgn0046689	**0.366**			
CG6908	FBgn0037936	**0.328**			
Wnt2	FBgn0004360	**0.304**			
Buffy	FBgn0040491	**0.297**	**0.258**		
CG9259	FBgn0032913	**0.252**			
CG32447	FBgn0052447	**0.228**			
CG31104	FBgn0051104	**0.133**			
Obp83ef	FBgn0046876	**0.063**	**0.106**		
CG11893	FBgn0039316	**0.045**	**40.01**		
**tissue structure**					
Cpr51A	FBgn0033942	**0.486**	**0.121**		
Cpr12A	FBgn0030494	**0.486**			
Sema-2b	FBgn0264273	**0.476**			
Pcp	FBgn0003046	**0.463**	**0.304**		
CG12009	FBgn0035430	**0.438**			
a	FBgn0000008	**0.420**			
CG11905	FBgn0036678	**0.374**			
Ama	FBgn0000071	**0.371**	**2.27**	**2.31**	**3.01**
TwdlE	FBgn0031957	**0.325**			
CG14957	FBgn0035412	**0.270**			
b	FBgn0000153	**0.250**			
Lcp65Ae	FBgn0020640	**0.245**			
mey	FBgn0039851	**0.235**			

Lcp65Ad	FBgn0020641	**0.230**			
Cpr65Ec	FBgn0035737	**0.230**	**2.41**		
Acp65Aa	FBgn0020765	**0.224**			
CG5756	FBgn0034301	**0.177**			
Cpr66Cb	FBgn0035875	**0.138**			
CG14687	FBgn0037835	**0.136**			
Muc68E	FBgn0053265	**0.080**			
**transport**					
Porin2	FBgn0069354	**0.493**			
CG7442	FBgn0037140	**0.490**	**0.403**		
CG5805	FBgn0039223	**0.486**			
CG31787	FBgn0051787	**0.483**			
Syx13	FBgn0036341	**0.480**			
Atet	FBgn0020762	**0.470**			**2.06**
CG8925	FBgn0038404	**0.438**			
Orct	FBgn0019952	**0.426**	**0.287**		
Tsp42Ek	FBgn0033133	**0.418**			
CG14855	FBgn0038260	**0.418**	**4.89**		
CG7777	FBgn0033635	**0.415**	**0.096**		
yin	FBgn0265575	**0.398**		**2.05**	
Glut1	FBgn0264574	**0.395**		**2.11**	
E23	FBgn0020445	**0.384**	**0.156**		
CG17036	FBgn0032449	**0.382**			
CG13426	FBgn0034510	**0.356**			
ZnT35C	FBgn0028516	**0.264**			
twz	FBgn0034636	**0.232**			**4.25**
CG3823	FBgn0029863	**0.212**			
CG31636	FBgn0051636	**0.210**			
CG2663	FBgn0037323	**0.209**			
Oatp26F	FBgn0051634	**0.193**			
CG1732	FBgn0039915	**0.189**	**2.49**		
bw	FBgn0000241	**0.163**			
sut2	FBgn0028562	**0.159**	**2.00**		
CG3649	FBgn0034785	**0.147**			
CG42269	FBgn0259164	**0.111**			
**unknown function**					
CG5780	FBgn0032446	**0.500**			
CG15525	FBgn0039732	**0.500**			
CG2837	FBgn0031646	**0.497**			
CG6685	FBgn0036062	**0.497**			
CG18249	FBgn0037553	**0.497**			
CG12674	FBgn0031388	**0.493**			
CG1124	FBgn0037290	**0.493**			**5.25**
CR6900	FBgn0030958	**0.493**	**0.489**		
CG11882	FBgn0039642	**0.490**			
CG5968	FBgn0032588	**0.490**			
CG13962	FBgn0032824	**0.490**	**2.09**		
CG15210	FBgn0040850	**0.490**	**0.350**		
CG9686	FBgn0030158	**0.486**			
HDC06936		**0.483**	**0.432**		
CG31600	FBgn0051600	**0.483**			
CG12880	FBgn0046258	**0.483**			

Atg18b	FBgn0032935	**0.480**			
l(1)G0469	FBgn0040153	**0.480**			
CG6675	FBgn0032973	**0.480**			
CG8031	FBgn0038110	**0.480**	**0.390**		
CG3831	FBgn0034804	**0.480**			**2.22**
IM18	FBgn0067903	**0.480**			
CG10933	FBgn0034264	**0.476**			
CG13905	FBgn0035176	**0.473**			
MESK4	FBgn0043069	**0.473**	**0.342**		**6.97**
Mur29B	FBgn0051901	**0.470**			
CT36057		**0.470**			
CG13618	FBgn0039203	**0.467**			
HDC06631		**0.467**	**2.80**		
CT34146		**0.463**			
CG32023	FBgn0052023	**0.460**			
LD44795	FBcl0167607	**0.457**			
TwdlT	FBgn0029170	**0.457**			
orb2	FBgn0264307	**0.457**			
CG42269	FBgn0259164	**0.457**			
CG1999	FBgn0029947	**0.457**			
CG31323	FBgn0051323	**0.457**	**0.254**		
CG13116	FBgn0032139	**0.454**		**3.50**	
CG31324	FBgn0051324	**0.454**			
CG5194	FBgn0035955	**0.454**			
CG10912	FBgn0034296	**0.448**			
CG33096	FBgn0053096	**0.444**			
CG11275	FBgn0034706	**0.444**	**0.279**		
CG18343	FBgn0033683	**0.444**			
spz6	FBgn0035056	**0.444**			
CG31538	FBgn0051538	**0.444**			
CG11474	FBgn0034688	**0.444**	**0.238**		
CG14567	FBgn0037126	**0.441**	**0.369**		
CG9837	FBgn0037635	**0.441**			**0.320**
CG34424	FBgn0085453	**0.441**			
CG13255	FBgn0040636	**0.438**	**0.270**		
CG3223	FBgn0037538	**0.438**			
CG32259	FBgn0052259	**0.438**			
CG31861	FBgn0051861	**0.432**			
CG13186	FBgn0033680	**0.432**			
CG14109	FBgn0036364	**0.432**	**8.40**		
CIAPIN1	FBgn0001977	**0.432**			
CG1273	FBgn0035522	**0.429**			
CG33252	FBgn0053252	**0.429**			
Gbp	FBgn0034199	**0.429**		**8.40**	**2.72**
CG18190	FBgn0034403	**0.429**	**8.35**		
CG13488	FBgn0034670	**0.426**			
CG8160	FBgn0034011	**0.423**	**12.11**		
CG9801	FBgn0037623	**0.423**	**0.360**		**4.13**
CG4438	FBgn0032115	**0.423**			
CG14275	FBgn0032022	**0.415**	**35.52**		
CG12655	FBgn0031080	**0.406**	**7.38**		
CG15544	FBgn0039804	**0.406**			**2.40**

LP03188	FBcl0187891	**0.406**			
CG15905	FBgn0034462	**0.403**			
NnaD	FBgn0265726	**0.401**			
HDC12400		**0.398**			
CG40294	FBgn0058294	**0.398**			
CG7841	FBgn0036502	**0.398**			
CG11737	FBgn0037592	**0.395**			
CG31525	FBgn0051525	**0.392**			
CG9040	FBgn0036394	**0.392**	**4.54**		
CR_TC_GH03576		**0.390**			
CG12470	FBgn0040371	**0.387**			
Dtg	FBgn0038071	**0.387**			
CG2444	FBgn0030326	**0.384**			
nyo	FBgn0039852	**0.374**			
CG15818	FBgn0031910	**0.374**			
CG30417	FBgn0050417	**0.371**			
HDC14735		**0.371**			
Ste	FBgn0003523	**0.366**			
CG15068	FBgn0040733	**0.363**	**14.12**		
RE54004	FBcl0204065	**0.361**			
DM.2L.4959		**0.361**			
CG7778	FBgn0032025	**0.356**			
CG2201	FBgn0032955	**0.349**			
CG13856	FBgn0038959	**0.342**			
CG32115	FBgn0052115	**0.332**	**0.362**		
CG12481	FBgn0030542	**0.325**			
CG13024	FBgn0036665	**0.325**			
CG13082	FBgn0032803	**0.321**			
CG1172	FBgn0264712	**0.316**			
CG2082	FBgn0027608	**0.314**	**0.284**		
CG15208	FBgn0030247	**0.308**			
CG33143	FBgn0053143	**0.304**			
CG15756	FBgn0030493	**0.301**			
CG10516	FBgn0036549	**0.293**			**2.10**
CG18622	FBgn0038460	**0.291**			
CG2082	FBgn0027608	**0.289**	**0.284**		
CG2277	FBgn0035204	**0.272**			
CG5391	FBgn0038943	**0.268**			
CG8483	FBgn0038126	**0.268**			
CG13722	FBgn0035553	**0.238**			
CG13314	FBgn0035949	**0.207**			
CG16836	FBgn0040735	**0.207**	**20.27**		
CG16886	FBgn0028938	**0.204**			
CG34296	FBgn0085325	**0.132**			
CG4151	FBgn0029770	**0.117**	**0.366**		
CR43242	FBgn0262887	**0.097**		**0.252**	
pncr015:3L	FBgn0063083	**0.086**			
CG5697	FBgn0038846	**0.045**			
CG9822	FBgn0034623	**0.036**			
CG42717	FBgn0261634	**0.032**			

Transcripts that are up- or down-regulated by homozygous null mutation of *Chd1* in L3 larvae or RNAi-mediated depletion of H1 in L3 salivary glands, HP1 in Kc cells and ISWI in SL2 cells. Fold change is calculated from Affymetrix microarray data relative to wild-type control.

Table S3 Overlap of transcripts regulated by HP1, H1 and CHD1.

SYMBOL/ TRANSCRIPT	FLYBASE ID	FOLD CHANGE		
		**HP1 KD**	**H1 KD**	***Chd1***
**HP1 depletion effect: *UP***				
CR31451	FBgn0051451	**181.09**	**8.26**	
CG31140	FBgn0051140	**79.80**	**6.31**	
G6	FBte0000958	**67.66**		
CG31642	FBgn0051642	**51.14**		**0.435**
AT07338	FBcl0481740	**50.00**		
TART		**35.15**		
CG3635	FBgn0032981	**34.71**	**6.77**	
CG18605	FBgn0034411	**24.98**		
Def	FBgn0010385	**22.33**		
CG10126	FBgn0038088	**21.46**		
springer	FBte0000333	**20.62**		
CG17124	FBgn0032297	**20.00**	**2.46**	
gypsy6	FBte0001175	**19.16**		
CG11714	FBgn0036170	**19.04**	**3.88**	
CG40295	FBgn0058295	**18.09**	**11.97**	
HDC20537		**16.14**	**2.13**	
CG5773	FBgn0034290	**15.22**		
CG13795	FBgn0031937	**13.84**		
CG5171	FBgn0031907	**12.85**		**0.149**
accord	FBte0000956	**11.76**		
CG5399	FBgn0038353	**11.64**	**28.01**	**2.06**
CG32091	FBgn0052091	**10.80**		
edin	FBgn0052185	**10.48**		
CG32032	FBgn0043806	**10.10**		
gypsy2	FBte0001040	**9.84**		
tok	FBgn0004885	**9.72**	**2.11**	
CG9691	FBgn0030160	**9.48**		
Yp3	FBgn0004047	**9.00**		**0.180**
invader3	FBte0000619	**8.99**		
CG13315	FBgn0040827	**8.90**		
CG3961	FBgn0036821	**8.86**		
CAH1	FBgn0027844	**8.65**	**2.25**	**0.493**
AttC	FBgn0041579	**8.50**		
gypsy	FBte0000021	**8.45**		
Cyp6a13	FBgn0033304	**8.40**	**5.64**	
Drs	FBgn0010381	**8.33**	**4.28**	**0.451**
Idgf1	FBgn0020416	**8.29**		
Cyp12e1	FBgn0037817	**8.15**		
HMS-Beagle	FBte0000726	**7.86**		
CG33775	FBgn0053775	**7.68**		
KCNQ	FBgn0033494	**7.66**		
Gpb5	FBgn0063368	**7.47**		
Gr94a	FBgn0041225	**7.33**	**5.19**	
CG33468	FBgn0053468	**7.30**	**4.91**	**2.46**
CG4752	FBgn0034733	**7.25**		
CG9981	FBgn0030746	**7.06**		**2.77**
aret	FBgn0000114	**6.89**		

CG45057	FBgn0266417	**6.74**	**2.20**	
Cyp6d4	FBgn0039006	**6.66**		
CG40115	FBgn0058115	**6.47**	**10.26**	
HDC20531		**6.34**		
Sid	FBgn0039593	**6.31**		**0.261**
CT39784		**6.11**	**14.84**	
HDC20523		**6.01**	**2.81**	
CG11672	FBgn0037563	**5.98**		
Tsf3	FBgn0034094	**5.89**		
Rh4	FBgn0003250	**5.82**		
CG10877	FBgn0038804	**5.73**	**0.157**	
CG9717	FBgn0039789	**5.71**		
CG18473	FBgn0037683	**5.69**	**3.14**	
bru-3	FBgn0264001	**5.67**		
rau	FBgn0031745	**5.63**		
CG15661	FBgn0034605	**5.55**	**12.72**	
Gbp	FBgn0034199	**5.52**		**0.429**
CG3940	FBgn0037788	**5.43**		**0.356**
CG10799	FBgn0033821	**5.41**	**3.26**	
PGRP-SD	FBgn0035806	**5.40**		
PGRP-SC2	FBgn0043575	**5.39**	**2.66**	**0.222**
Cyp12e1	FBgn0037817	**5.34**		
CG14164	FBgn0036057	**5.31**		
AttA	FBgn0012042	**5.30**		**0.337**
CG4928	FBgn0027556	**5.30**	**0.315**	
CG14695	FBgn0037850	**5.29**		
Zasp52	FBgn0265991	**5.26**	**0.492**	
CG5367	FBgn0032228	**5.24**		
CG18446	FBgn0033458	**5.20**	**2.05**	**0.395**
Mct1	FBgn0023549	**5.19**	**0.395**	
invader4	FBte0000292	**5.17**		
Pka-C3	FBgn0000489	**5.16**		
CG17124	FBgn0032297	**5.14**	**4.46**	
CG9919	FBgn0030742	**5.13**		
SPH93	FBgn0032638	**5.12**	**11.67**	
Corp	FBgn0030028	**5.08**	**4.57**	
Apoltp	FBgn0032136	**5.06**	**2.89**	**5.70**
spir	FBgn0003475	**5.06**		
CG8586	FBgn0033320	**5.03**		**0.438**
CG11052	FBgn0040524	**5.03**		
yin	FBgn0265575	**5.00**	**0.449**	**0.398**
CG43110	FBgn0262570	**4.99**	**0.432**	
Cyp9c1	FBgn0015040	**4.93**		**3.41**
CG5381	FBgn0032218	**4.85**		
CG32687	FBgn0052687	**4.82**	**2.09**	
Cad96Ca	FBgn0022800	**4.81**		
Tsp2A	FBgn0024361	**4.77**		
Cyp28d1	FBgn0031689	**4.76**		
Mctp	FBgn0034389	**4.72**	**0.300**	**2.95**
Ama	FBgn0000071	**4.72**	**2.27**	**0.371**
Jheh3	FBgn0034406	**4.69**		**2.68**
alphaTub67C	FBgn0087040	**4.63**		

CG5731	FBgn0032192	**4.60**	**2.72**	
GstE3	FBgn0063497	**4.59**		**0.432**
CG40124	FBgn0058124	**4.58**	**11.10**	
CG5853	FBgn0032167	**4.57**		
CT39116		**4.56**	**103.1**	
CG14606	FBgn0037485	**4.54**		
shf	FBgn0003390	**4.52**	**2.97**	
CG1294	FBgn0033030	**4.49**	**4.55**	
ItgalphaPS4	FBgn0034005	**4.49**	**3.91**	
PGRP-SA	FBgn0030310	**4.46**	**2.20**	
Cyp4p3	FBgn0033397	**4.43**	**9.73**	**8.75**
CG6709	FBgn0036056	**4.43**	**2.66**	
HDC20116		**4.41**		
Cyp12a5	FBgn0038680	**4.30**		
CG12698	FBgn0030721	**4.26**		
CG9170	FBgn0030716	**4.26**	**3.83**	
qbert		**4.26**		
RpS5b	FBgn0038277	**4.25**		
AttB	FBgn0041581	**4.22**		
Hf	FBgn0014000	**4.22**		
l(3)neo38	FBgn0265276	**4.17**		
HDC20280		**4.17**		
CG6330	FBgn0039464	**4.15**		
beat-IIIc	FBgn0032629	**4.13**	**2.39**	
alphaTub85E	FBgn0003886	**4.12**		
Swim	FBgn0034709	**4.11**	**9.06**	
Mal-A5	FBgn0050359	**4.11**		
GstE11	FBgn0034354	**4.06**	**0.341**	
CG2930	FBgn0028491	**4.06**		
Eip78C	FBgn0004865	**4.04**	**0.136**	**0.144**
Epac	FBgn0085421	**4.01**	**9.43**	
CG3246	FBgn0031538	**4.00**		
TpnC4	FBgn0033027	**4.00**	**3.70**	
TART		**3.97**		
Myo28B1	FBgn0040299	**3.95**	**2.21**	
CG14451	FBgn0037183	**3.94**		
kek1	FBgn0015399	**3.90**	**5.43**	
Esyt2	FBgn0266758	**3.88**		
CG13117	FBgn0032140	**3.85**	**2.71**	
CG40274	FBgn0058274	**3.85**	**5.22**	
micropia	FBte0000042	**3.83**		
CG5322	FBgn0032253	**3.80**		
CG11400	FBgn0034198	**3.78**	**19.24**	
CG16947	FBgn0031816	**3.78**	**0.285**	
GATE	FBte0000359	**3.78**		
NT5E-2	FBgn0050104	**3.76**	**0.196**	
CG9150	FBgn0031775	**3.72**		
spir	FBgn0003475	**3.67**		
Ir41a	FBgn0040849	**3.67**		
Tsp42Ed	FBgn0029507	**3.66**		
l(3)neo38	FBgn0265276	**3.65**		
l(3)neo38	FBgn0265276	**3.65**		

CG12868	FBgn0033945	**3.64**	**2.74**	
CG17032	FBgn0036547	**3.63**		
CG31272	FBgn0051272	**3.62**		
Hsp68	FBgn0001230	**3.60**		
loh	FBgn0032252	**3.59**	**2.15**	
Tl	FBgn0262473	**3.58**	**2.30**	
sdk	FBgn0021764	**3.58**		
CG4301	FBgn0030747	**3.58**		
aret	FBgn0000114	**3.58**		
rdgB	FBgn0003218	**3.58**		
bgm	FBgn0027348	**3.58**	**4.62**	
vir-1	FBgn0043841	**3.56**	**8.76**	
CG43658	FBgn0263706	**3.53**		
LysX	FBgn0004431	**3.51**	**33.21**	**0.344**
eIF4E-6	FBgn0039622	**3.50**		
fend	FBgn0030090	**3.47**	**3.52**	
CG3679	FBgn0027521	**3.43**	**4.21**	
wrapper	FBgn0025878	**3.42**		
Glut1	FBgn0264574	**3.36**	**0.312**	**0.395**
CG34232	FBgn0085261	**3.35**		
CG12926	FBgn0033437	**3.34**	**0.415**	
HDC20112		**3.32**		
HMS-Beagle	FBte0000726	**3.32**		
Pepck	FBgn0003067	**3.29**		**2.30**
Mtk	FBgn0014865	**3.29**		**0.438**
Listericin	FBgn0033593	**3.28**		
Ugt35a	FBgn0026315	**3.27**	**3.35**	**2.53**
Ndae1	FBgn0259111	**3.26**		**2.10**
rdgB	FBgn0003218	**3.26**		
CG4269	FBgn0034741	**3.25**		
sda	FBgn0015541	**3.24**		
CG17207	FBgn0038051	**3.24**		
mthl14	FBgn0052476	**3.23**	**15.70**	
Spn31A	FBgn0032178	**3.22**		
CG5096	FBgn0032235	**3.19**	**3.96**	
nerfin-2	FBgn0041105	**3.18**		
CG10405	FBgn0038431	**3.16**		
hiw	FBgn0030600	**3.15**		
Arc1	FBgn0033926	**3.15**		
CG6357	FBgn0033875	**3.14**	**10.96**	
Nep2	FBgn0027570	**3.13**		**0.283**
d	FBgn0262029	**3.13**		
AttD	FBgn0038530	**3.13**	**72.76**	
Fie	FBgn0026592	**3.12**	**2.46**	
CG40040	FBgn0058040	**3.10**		
l(3)neo38	FBgn0265276	**3.10**		
CG32625	FBgn0052625	**3.09**	**39.17**	
CG9360	FBgn0030332	**3.06**	**10.35**	
CG3568	FBgn0029710	**3.03**		
CG31626	FBgn0051626	**3.00**	**19.15**	
CG32313	FBgn0052313	**3.00**		
spir	FBgn0003475	**3.00**	**2.15**	

tyn	FBgn0029128	**3.00**		
hdm	FBgn0029977	**2.98**		
CG42822	FBgn0262004	**2.98**		
CG32944	FBgn0052944	**2.97**		
CG33346	FBgn0053346	**2.97**		
alpha-Est3	FBgn0015571	**2.97**		
CG5191	FBgn0038803	**2.97**	**0.403**	
CG4297	FBgn0031258	**2.97**	**0.429**	
dys	FBgn0039411	**2.96**		
Ef1alpha100E	FBgn0000557	**2.95**		
QC	FBgn0052412	**2.95**		
stv	FBgn0086708	**2.94**		**2.53**
ZnT77C	FBgn0037000	**2.92**		
CG8738	FBgn0033321	**2.91**		
CG3251	FBgn0031622	**2.90**		
d	FBgn0262029	**2.90**		
Idgf2	FBgn0020415	**2.90**	**2.56**	
CG33494	FBgn0053494	**2.89**		
CG16749	FBgn0037678	**2.88**		
HDC20377		**2.86**		
CG33144	FBgn0053144	**2.86**		
Mdr49	FBgn0004512	**2.86**	**3.38**	
Cyp6a8	FBgn0013772	**2.83**		
Cyp6a2	FBgn0000473	**2.80**		**30.5**
RapGAP1	FBgn0264895	**2.80**	**0.339**	
CG6495	FBgn0027550	**2.80**		
CG32982	FBgn0052982	**2.79**	**2.33**	
Cyp4p1	FBgn0015037	**2.79**		
GstD5	FBgn0010041	**2.78**	**20.38**	
Het-A		**2.75**		
Kaz1-ORFB	FBgn0063923	**2.75**	**4.27**	
CG30456	FBgn0050456	**2.74**		
wisp	FBgn0260780	**2.74**		
CG8349	FBgn0032003	**2.73**		
CHKov2	FBgn0039328	**2.72**		
CG8046	FBgn0033388	**2.71**		
R1		**2.71**		
Rbf	FBgn0015799	**2.71**		
CG4741	FBgn0035040	**2.70**		
CG2217	FBgn0027544	**2.69**	**0.431**	
Ugt86Da	FBgn0040259	**2.68**	**8.97**	**2.28**
btn	FBgn0014949	**2.68**		
Jhl-26	FBgn0028424	**2.68**	**7.00**	**3.84**
AOX1	FBgn0267408	**2.67**	**14.75**	**2.60**
stai	FBgn0266521	**2.67**		
Ir41a	FBgn0040849	**2.66**		
Ugt86Dd	FBgn0040256	**2.65**	**4.15**	
PKD	FBgn0038603	**2.65**		**2.04**
Thor	FBgn0261560	**2.63**		
CG4398	FBgn0034126	**2.62**	**0.052**	
CG14692	FBgn0037836	**2.61**		
CG16712	FBgn0031561	**2.60**	**7.20**	**2.48**

Arc1	FBgn0033926	**2.59**	**5.93**	
Rhp	FBgn0026374	**2.57**	**0.328**	
COX7AL	FBgn0037579	**2.57**		
CG8066	FBgn0038243	**2.56**	**5.94**	
Muc14A	FBgn0052580	**2.56**		
CG15611	FBgn0034194	**2.56**		
Gs2	FBgn0001145	**2.55**	**3.09**	
CG33784	FBgn0053784	**2.55**		
KFase	FBgn0031821	**2.53**		
Gnmt	FBgn0038074	**2.53**	**8.13**	
CG17549	FBgn0032774	**2.52**		
pirk	FBgn0034647	**2.51**		
Doc2	FBgn0035956	**2.50**		
Hsp67Bc	FBgn0001229	**2.49**	**2.74**	**0.200**
egr	FBgn0033483	**2.49**		
CG7191	FBgn0031945	**2.48**		
CG3397	FBgn0037975	**2.48**		**0.162**
GstE7	FBgn0063493	**2.48**		**8.94**
GstE10	FBgn0063499	**2.48**		
CG11897	FBgn0039644	**2.48**	**5.52**	**2.14**
ome	FBgn0259175	**2.47**		
CG30022	FBgn0050022	**2.47**		
CG8008	FBgn0033387	**2.46**		
CG4822	FBgn0031220	**2.45**	**0.410**	
CG5783	FBgn0032670	**2.45**	**0.388**	
spri	FBgn0085443	**2.45**		
Traf4	FBgn0026319	**2.44**		
CG4829	FBgn0030796	**2.43**		
be	FBgn0052594	**2.42**		
CG8870	FBgn0038144	**2.41**		
Ptth	FBgn0013323	**2.39**		
KP78b	FBgn0026063	**2.38**	**6.25**	
CG14692	FBgn0037836	**2.38**		
Ror	FBgn0010407	**2.38**		
CG14642	FBgn0037222	**2.37**	**3.23**	
pdm3	FBgn0261588	**2.37**		
CG17029	FBgn0036551	**2.37**		
CG1358	FBgn0033196	**2.36**		
CG13813	FBgn0036956	**2.36**		
egr	FBgn0033483	**2.36**		
Inx3	FBgn0265274	**2.35**		
HDC20468		**2.35**	**8.59**	
CG6287	FBgn0032350	**2.35**		**0.382**
CG43064	FBgn0262366	**2.34**		
Pask	FBgn0034950	**2.32**		
DptB	FBgn0034407	**2.32**		
CG32521	FBgn0052521	**2.32**		
mtt	FBgn0050361	**2.32**		
Mef2	FBgn0011656	**2.32**		
CG30022	FBgn0050022	**2.31**		
CG18547	FBgn0037973	**2.30**	**4.73**	
Mmp1	FBgn0035049	**2.30**	**15.55**	**0.264**

GstD2	FBgn0010038	**2.30**	**9.61**	**0.235**
Nha2	FBgn0263390	**2.29**	**2.28**	**6.11**
IP3K2	FBgn0266375	**2.29**		
CG13116	FBgn0032139	**2.29**		**0.454**
Sip1	FBgn0010620	**2.29**	**0.396**	
CG4502	FBgn0031896	**2.28**		
Ect4	FBgn0262579	**2.28**		**2.06**
yellow-f	FBgn0041710	**2.27**	**0.330**	**0.358**
CG31743	FBgn0032618	**2.27**		**2.60**
tilB	FBgn0014395	**2.27**		
CG13893	FBgn0035146	**2.27**		
l(2)03659	FBgn0010549	**2.26**	**3.15**	
CR32207	FBgn0052207	**2.26**	**12.38**	
GstE2	FBgn0063498	**2.25**		
CG7255	FBgn0036493	**2.24**	**0.448**	
CG31253	FBgn0051253	**2.24**		
eater	FBgn0243514	**2.24**	**8.00**	
HDC20224		**2.23**	**5.63**	
CG7203	FBgn0031942	**2.23**		
CG13912	FBgn0035186	**2.23**		**2.27**
CG42240	FBgn0250869	**2.23**	**0.403**	
CG13698	FBgn0036773	**2.23**		
CG11686	FBgn0040551	**2.22**		
CG44325	FBgn0265413	**2.21**		
rst	FBgn0003285	**2.21**	**13.17**	
GstT4	FBgn0030484	**2.21**	**2.14**	**2.11**
CG7627	FBgn0032026	**2.21**		
IP3K2	FBgn0266375	**2.21**		
CG30485	FBgn0050485	**2.20**		
CG31038	FBgn0051038	**2.20**		
regucalcin	FBgn0030362	**2.20**		
CG14857	FBgn0038262	**2.20**		
GlyP	FBgn0004507	**2.19**		
Lsd-1	FBgn0039114	**2.19**		
CG3168	FBgn0029896	**2.19**		
Dyrk2	FBgn0016930	**2.18**		**0.448**
Tsp42Ea	FBgn0029508	**2.17**		
CG18063	FBgn0028856	**2.17**		
dnr1	FBgn0260866	**2.17**		
Rab23	FBgn0037364	**2.16**	**3.31**	
CG4404	FBgn0030432	**2.16**	**2.28**	
CG14291	FBgn0038660	**2.16**		
bbg	FBgn0087007	**2.15**	**0.392**	
Hsp23	FBgn0001224	**2.15**		
clos	FBgn0261016	**2.15**		
CG32368	FBgn0052368	**2.13**		
CG43164	FBgn0262720	**2.12**		
CG13654	FBgn0039290	**2.12**	**2.49**	
CG12428	FBgn0039543	**2.12**		
sick	FBgn0263873	**2.11**		
CG31436	FBgn0051436	**2.11**		
CG44251	FBgn0265186	**2.11**	**2.21**	

Inx2	FBgn0027108	**2.11**		
CG2065	FBgn0033204	**2.11**	**9.00**	**0.163**
CDase	FBgn0039774	**2.10**	**2.07**	
PGRP-SB1	FBgn0043578	**2.10**		**0.108**
CG12825	FBgn0033221	**2.10**	**2.64**	
brp	FBgn0259246	**2.09**		
CG13650	FBgn0039277	**2.08**		
MESK2	FBgn0043070	**2.08**		
CG31274	FBgn0051274	**2.08**	**0.326**	**0.418**
CG10283	FBgn0032681	**2.08**		
ebd2	FBgn0037076	**2.08**		
Ela	FBgn0013949	**2.08**		
pyd	FBgn0262614	**2.06**		
CG6231	FBgn0038720	**2.06**		
CecB	FBgn0000278	**2.06**		
CG14636	FBgn0037217	**2.05**	**0.355**	
veil	FBgn0034225	**2.05**		
CG43340	FBgn0263077	**2.05**		**2.22**
CG30460	FBgn0050460	**2.05**		
Cyp6d5	FBgn0038194	**2.05**		**0.490**
CG31705	FBgn0028490	**2.05**		
PGRP-LA	FBgn0035975	**2.05**		
CG6701	FBgn0033889	**2.05**		
GstD9	FBgn0038020	**2.04**		
pdgy	FBgn0027601	**2.03**		
spri	FBgn0085443	**2.03**		
mlt	FBgn0265512	**2.03**	**4.24**	
GstE6	FBgn0063494	**2.03**	**43.50**	**4.17**
CG32436	FBgn0052436	**2.03**		
Cyp4e2	FBgn0014469	**2.03**		
Gel	FBgn0010225	**2.02**		
Sans	FBgn0033785	**2.02**		
Tep4	FBgn0041180	**2.02**	**3.86**	
fat-spondin	FBgn0026721	**2.01**		
HDC03722		**2.00**		
HP1D3csd	FBgn0030994	**2.00**		
su(r)	FBgn0086450	**2.00**	**3.20**	**2.17**
Ih	FBgn0263397	**2.00**	**2.34**	
**HP1 depletion effect: *DOWN***				
CG32850	FBgn0052850	**0.498**	**4.43**	
CG41128	FBgn0069923	**0.490**	**0.338**	
CG3262	FBgn0032986	**0.486**		
Dbp80	FBgn0024804	**0.480**		
CG32350	FBgn0052350	**0.480**		
Acf1	FBgn0027620	**0.480**		
Fas2	FBgn0000635	**0.479**		**2.11**
ND-AGGG	FBgn0058002	**0.477**		
PlexB	FBgn0025740	**0.474**		
COX4L	FBgn0033020	**0.464**		
PlexB	FBgn0025740	**0.455**		
CG34330	FBgn0085359	**0.452**	**4.33**	
CG6329	FBgn0033872	**0.435**		

Nipped-A	FBgn0053554	**0.430**		
CG2709	FBgn0024977	**0.425**		
l(2)41Ab	FBgn0262123	**0.417**	**4.24**	
dob	FBgn0030607	**0.402**	**3.62**	
TpnC41C	FBgn0013348	**0.398**		
CG3829	FBgn0035091	**0.394**		
HDC20114		**0.370**		
Ser	FBgn0004197	**0.366**	**0.385**	
Transpac	FBte0001377	**0.363**		
CG41099	FBgn0039955	**0.362**		
CG3788	FBgn0034800	**0.353**		
CG17698	FBgn0040056	**0.347**		
br	FBgn0000210	**0.327**		
slgA	FBgn0003423	**0.316**	**0.345**	
mos	FBgn0033773	**0.309**		
Caps	FBgn0053653	**0.284**		
CR43242	FBgn0262887	**0.252**		**0.097**
CG40160	FBgn0058160	**0.239**	**0.441**	
CR43242	FBgn0262887	**0.239**		**0.097**
CG40006	FBgn0058006	**0.230**	**28.55**	
CG40294	FBgn0058294	**0.157**		**0.398**
CG30440	FBgn0050440	**0.132**		
Maf1	FBgn0267861	**0.129**		
Caps	FBgn0053653	**0.116**		
CR45923	FBgn0267585	**0.093**	**6.95**	
CG17698	FBgn0040056	**0.093**		
CG32017	FBgn0052017	**0.058**	**5.13**	
Cht3	FBgn0250907	**0.029**		
Su(var)205	FBgn0003607	**0.025**		

Transcripts that are up- or down-regulated by RNAi-mediated depletion of HP1 in Kc cells and H1 in L3 salivary glands or a homozygous null mutation of *Chd1* in L3 larvae. Fold change is calculated from Affymetrix microarray data relative to wild-type control. Transposons, repetitive and presumed heterochromatic sequences (Chr Xh, Chr 2h, Chr 3h and Chr4) are shown in red type.

Table S4 Overlap of transcripts regulated by ISWI, H1 and CHD1.

SYMBOL/ TRANSCRIPT	FLYBASE ID	FOLD CHANGE		
		ISWI KD	H1 KD	*Chd1*
**ISWI depletion effect:** ***UP***				
CG42675	FBgn0261561	**48.78**	**0.376**	
Ance-5	FBgn0035076	**37.27**	**2.73**	
CG33225	FBgn0053225	**27.57**	**31.76**	
Acer	FBgn0016122	**24.68**	**3.27**	
CG14787	FBgn0027793	**17.33**		**2.91**
CG33465	FBgn0053465	**16.53**	**2.80**	
CG42807	FBgn0261989	**16.34**		
CG9008	FBgn0028540	**15.47**		
Cyp6d5	FBgn0038194	**13.46**		**0.490**
CG45057	FBgn0266417	**13.26**	**2.20**	
GstD7	FBgn0010043	**12.65**	**3.98**	**3.61**
CG8399	FBgn0034067	**12.34**	**5.09**	
CG30091	FBgn0050091	**11.62**		**0.187**
yellow-f2	FBgn0038105	**11.48**	**5.78**	
nvy	FBgn0005636	**10.70**		
Obp44a	FBgn0033268	**10.65**	**32.99**	
zye	FBgn0036985	**10.55**		
ClC-a	FBgn0051116	**10.16**		
CG6357	FBgn0033875	**9.69**	**10.96**	
pgant2	FBgn0031530	**9.31**		
CG12910	FBgn0033502	**9.24**		
CG15202	FBgn0030271	**9.00**	**0.412**	
CG5397	FBgn0031327	**8.87**	**3.33**	
CG13077	FBgn0032810	**8.67**		
Cht2	FBgn0022702	**8.62**		
NimB3	FBgn0054003	**8.52**		
CG31219	FBgn0051219	**8.14**		
CG17321	FBgn0032719	**7.96**		
CG5731	FBgn0032192	**7.79**	**2.72**	
CG33120	FBgn0053120	**7.62**		
wdp	FBgn0034718	**7.45**		
Ggamma30A	FBgn0267252	**7.22**	**3.07**	
CG32483	FBgn0052483	**7.18**		**2.77**
Sodh-2	FBgn0022359	**7.12**		
CG17681	FBgn0032668	**7.00**		
CG10126	FBgn0038088	**6.97**		
MESK4	FBgn0043069	**6.97**	**0.342**	**0.473**
Obp49a	FBgn0050052	**6.88**		
CG10311	FBgn0038420	**6.87**		
fng	FBgn0011591	**6.74**		
Gli	FBgn0001987	**6.60**		
CG16710	FBgn0039101	**6.33**		
CG14990	FBgn0035496	**6.14**		
Mtk	FBgn0014865	**6.09**		**0.438**
mthl14	FBgn0052476	**6.02**	**15.70**	
CG11638	FBgn0040351	**5.97**		

CG30345	FBgn0050345	**5.74**		
CG5001	FBgn0031322	**5.55**		
e	FBgn0000527	**5.46**	**2.93**	**0.042**
CG42369	FBgn0259715	**5.45**	**7.66**	
Mdr49	FBgn0004512	**5.34**	**3.38**	
RpS30	FBgn0038834	**5.33**		
CG1124	FBgn0037290	**5.25**		**0.493**
CG31274	FBgn0051274	**5.24**	**0.326**	**0.418**
Kal1	FBgn0039155	**5.17**	**0.168**	
dmGlut	FBgn0010497	**5.17**	**0.167**	
CG17029	FBgn0036551	**5.17**		
Fuca	FBgn0036169	**5.14**	**3.88**	
Tsp42Ea	FBgn0029508	**5.03**		
qsm	FBgn0028622	**4.98**		
lea	FBgn0002543	**4.97**	**3.29**	
yellow-c	FBgn0041713	**4.92**		**0.146**
cad	FBgn0000251	**4.91**	**8.04**	
CG6830	FBgn0037934	**4.83**		
dpr17	FBgn0051361	**4.81**		
CG13377	FBgn0261446	**4.65**		
CG11378	FBgn0040364	**4.62**	**0.331**	
CG9691	FBgn0030160	**4.55**		
ChLD3	FBgn0032598	**4.44**		
CG17751	FBgn0038717	**4.43**		
CG13921	FBgn0035267	**4.27**	**4.49**	
twz	FBgn0034636	**4.25**		**0.232**
CG30148	FBgn0050148	**4.22**		
CG7966	FBgn0038115	**4.19**		
CG31743	FBgn0032618	**4.18**		**2.60**
Snap25	FBgn0011288	**4.17**	**33.90**	
CG9801	FBgn0037623	**4.13**	**0.360**	**0.423**
CG14516	FBgn0039640	**4.05**		
Gs1	FBgn0001142	**3.89**		
CG10359	FBgn0035452	**3.89**		
Ude	FBgn0039226	**3.85**		**5.03**
Drs	FBgn0010381	**3.78**	**4.28**	**0.451**
CG7607	FBgn0036145	**3.71**	**3.73**	
CG15772	FBgn0029799	**3.70**		
sPLA2	FBgn0033170	**3.67**		
CG32313	FBgn0052313	**3.63**		
twit	FBgn0032895	**3.59**		
PCB	FBgn0027580	**3.58**		
Gyc-89Db	FBgn0038436	**3.56**		
Cyp6d2	FBgn0034756	**3.53**		
CG16789	FBgn0037712	**3.47**		
Gk	FBgn0035266	**3.39**		
Cby	FBgn0067317	**3.38**		
CG7860	FBgn0030653	**3.36**		
Mmp1	FBgn0035049	**3.34**	**11.63**	**0.264**
yellow-b	FBgn0032601	**3.34**		
Pka-C3	FBgn0000489	**3.28**		
CG7781	FBgn0032021	**3.26**		

Cyp6a17	FBgn0015714	**3.22**	**3.26**	
CG45263	FBgn0266801	**3.22**		
GlcAT-S	FBgn0032135	**3.20**	**0.331**	
CG10877	FBgn0038804	**3.18**	**0.157**	
btn	FBgn0014949	**3.16**		
PlexB	FBgn0025740	**3.14**		
CG3505	FBgn0038250	**3.10**		
CG30380	FBgn0050380	**3.09**		
SkpB	FBgn0026176	**3.08**		
GLaz	FBgn0033799	**3.07**	**4.64**	
Cyp6a23	FBgn0033978	**3.07**	**16.25**	**5.98**
Dhc36C	FBgn0013810	**3.07**		
CG15343	FBgn0030029	**3.07**	**0.124**	**0.423**
ft	FBgn0001075	**3.06**		
MESK2	FBgn0043070	**3.04**		
CG6218	FBgn0038321	**3.01**		
Ama	FBgn0000071	**3.01**	**2.27**	**0.371**
Oat	FBgn0022774	**3.00**	**0.077**	**0.306**
fax	FBgn0014163	**2.98**		
CG34288	FBgn0085317	**2.96**		
baz	FBgn0000163	**2.96**	**0.369**	
CG4630	FBgn0033809	**2.95**	**0.301**	
CG5783	FBgn0032670	**2.95**	**0.388**	
LamC	FBgn0010397	**2.95**		**2.08**
CG3625	FBgn0031245	**2.92**		
Cyp6a22	FBgn0013773	**2.92**	**7.50**	
PPO1	FBgn0261362	**2.92**	**6.89**	
CG12262	FBgn0035811	**2.91**		
miple2	FBgn0029002	**2.87**		
LpR1	FBgn0066101	**2.87**		
Jheh1	FBgn0010053	**2.85**		
slgA	FBgn0003423	**2.85**	**0.345**	
Pepck	FBgn0003067	**2.84**		**2.30**
Cyp4d20	FBgn0035344	**2.81**		
CG5577	FBgn0036759	**2.81**	**0.359**	
CG34409	FBgn0085438	**2.80**		
brv3	FBgn0040333	**2.80**		
AdamTS-A	FBgn0038341	**2.79**	**0.207**	
rgn	FBgn0261258	**2.78**	**11.07**	
Dat	FBgn0019643	**2.74**		
CG8547	FBgn0033919	**2.72**		
Gbp	FBgn0034199	**2.72**		**0.429**
CG10232	FBgn0039108	**2.68**	**4.13**	
CG14036	FBgn0031677	**2.68**		
HDC15448		**2.66**		
corolla	FBgn0267967	**2.66**		
Ror	FBgn0010407	**2.66**		
pdgy	FBgn0027601	**2.66**		
fra	FBgn0011592	**2.65**		
CG1969	FBgn0039690	**2.65**	**0.218**	
S.C3R003414		**2.64**		
wus	FBgn0030805	**2.62**		

CG8353	FBgn0032002	**2.62**		
CG10764	FBgn0034221	**2.61**		**0.390**
CG30503	FBgn0050503	**2.60**		**3.14**
CG11983	FBgn0037654	**2.60**		
CG11897	FBgn0039644	**2.59**	**5.52**	**2.14**
Mmp1	FBgn0035049	**2.57**	**9.53**	**0.264**
Cyp310a1	FBgn0032693	**2.57**	**6.31**	
Cyp4d2	FBgn0011576	**2.57**		
whd	FBgn0261862	**2.56**		
CG8066	FBgn0038243	**2.55**	**5.94**	
betaTub60D	FBgn0003888	**2.55**	**3.13**	
CG32436	FBgn0052436	**2.53**		
Cyp6a20	FBgn0033980	**2.52**		
Cyp12a4	FBgn0038681	**2.52**	**3.42**	
Src64B	FBgn0262733	**2.50**		
c(2)M	FBgn0028525	**2.50**	**2.93**	
Npc2b	FBgn0038198	**2.49**		
CG42390	FBgn0259736	**2.48**	**0.464**	**2.51**
mbc	FBgn0015513	**2.47**		
CG31751	FBgn0086909	**2.47**	**0.375**	
Adk3	FBgn0042094	**2.47**		
Spn27A	FBgn0028990	**2.47**		
dpp	FBgn0000490	**2.46**		
Mmp1	FBgn0035049	**2.46**	**15.55**	**0.264**
CG17549	FBgn0032774	**2.44**		
CG34331	FBgn0085360	**2.44**		
Hydr1	FBgn0033382	**2.43**		
CG32280	FBgn0052280	**2.43**		
Tequila	FBgn0023479	**2.43**		
Atpalpha	FBgn0002921	**2.42**		
nrv3	FBgn0032946	**2.42**	**8.16**	
Hsp27	FBgn0001226	**2.41**		**0.457**
corn	FBgn0259173	**2.41**	**12.44**	
CG17350	FBgn0032772	**2.40**		
CG15544	FBgn0039804	**2.40**		**0.406**
CG8501	FBgn0033724	**2.40**		
CG12206	FBgn0029662	**2.40**		
GlyP	FBgn0004507	**2.39**		
Tsp42Ed	FBgn0029507	**2.39**		
NtR	FBgn0029147	**2.39**	**2.51**	
CG7142	FBgn0038595	**2.38**	**5.56**	
CG15093	FBgn0034390	**2.37**	**2.02**	
CDase	FBgn0039774	**2.36**	**2.07**	
sano	FBgn0034408	**2.35**	**4.83**	
EF-G2	FBgn0051159	**2.35**		
Dmtn	FBgn0037443	**2.34**		
CG1969	FBgn0039690	**2.34**	**0.189**	
CG13907	FBgn0035173	**2.33**		
sdk	FBgn0021764	**2.33**		
CG40115	FBgn0058115	**2.33**	**10.26**	
CG10824	FBgn0038865	**2.30**	**2.24**	
CG14984	FBgn0035480	**2.30**	**2.07**	

Jabba	FBgn0259682	**2.30**		
Cyp6a13	FBgn0033304	**2.30**	**5.64**	
CG17919	FBgn0037433	**2.29**	**9.35**	
Ance	FBgn0012037	**2.28**		
CG18508	FBgn0028746	**2.28**		
CG33111	FBgn0053111	**2.28**		
Itgbetanu	FBgn0010395	**2.27**		
CG43658	FBgn0263706	**2.27**		
CG16947	FBgn0031816	**2.27**	**0.285**	
Cyp12d1-d	FBgn0053503	**2.26**		
CG33181	FBgn0053181	**2.26**	**7.63**	
CG13252	FBgn0037016	**2.25**	**0.440**	
Asph	FBgn0034075	**2.25**	**0.427**	**0.308**
dnd	FBgn0038916	**2.25**		
Mctp	FBgn0034389	**2.25**	**0.300**	**2.95**
CG15914	FBgn0030700	**2.24**		
dlp	FBgn0041604	**2.24**		
bgcn	FBgn0004581	**2.23**		
Apoltp	FBgn0032136	**2.22**	**2.89**	**5.70**
CG40115	FBgn0058115	**2.22**	**7.20**	
CG3831	FBgn0034804	**2.22**		**0.480**
CG5853	FBgn0032167	**2.20**		
CG12746	FBgn0037341	**2.20**		
springer	FBte0000333	**2.19**		
SD02481	FBcl0277517	**2.18**		
fat-spondin	FBgn0026721	**2.18**		
Fem-1	FBgn0034542	**2.17**		
Oatp30B	FBgn0032123	**2.17**		
mthl5	FBgn0037960	**2.17**		**0.476**
SAK	FBgn0026371	**2.16**		
Vha68-1	FBgn0265262	**2.15**	**2.42**	
norpA	FBgn0262738	**2.15**		
Ect3	FBgn0260746	**2.15**	**2.21**	
CG3038	FBgn0040373	**2.14**		
CG32032	FBgn0043806	**2.14**		
CG9547	FBgn0031824	**2.13**		
CG12643	FBgn0040942	**2.12**		
CG31974	FBgn0051974	**2.12**	**4.74**	**2.23**
CAH1	FBgn0027844	**2.11**	**2.25**	**0.493**
mtg	FBgn0260386	**2.11**	**2.25**	**2.83**
CG30441	FBgn0050441	**2.11**		
TM4SF	FBgn0020372	**2.11**		
Cpr49Ag	FBgn0033730	**2.10**		
ImpE3	FBgn0001255	**2.10**		**0.171**
CG10516	FBgn0036549	**2.10**		**0.293**
zuc	FBgn0261266	**2.09**		
Tret1-1	FBgn0050035	**2.07**		
Muc68Ca	FBgn0036181	**2.07**		**7.36**
CG17754	FBgn0030114	**2.07**		**0.490**
CG45186	FBgn0266696	**2.06**	**0.433**	
Atet	FBgn0020762	**2.06**		**0.470**
CG5080	FBgn0031313	**2.06**		

cyc	FBgn0023094	**2.05**		
CG33947	FBgn0083068	**2.05**		
SLC5A11	FBgn0031998	**2.05**		
CG14523	FBgn0039612	**2.04**		
CG42684	FBgn0261570	**2.04**		
CG17265	FBgn0031488	**2.03**		
CG42327	FBgn0259227	**2.03**		
CG5096	FBgn0032235	**2.03**	**3.96**	
CG9914	FBgn0030737	**2.03**	**4.84**	
Dip3	FBgn0040465	**2.03**		
mthl10	FBgn0035132	**2.03**		
Dark	FBgn0263864	**2.02**	**0.249**	**0.418**
Apf	FBgn0051713	**2.02**		
CG6330	FBgn0039464	**2.01**		
ItgalphaPS4	FBgn0034005	**2.01**	**3.91**	
FER	FBgn0000723	**2.01**		
CG41128	FBgn0069923	**2.00**	**0.338**	
**ISWI depletion effect: *DOWN***				
CG40191	FBgn0058191	**0.498**		
laccase2	FBgn0259247	**0.498**		
CG7322	FBgn0030968	**0.495**		
ppk29	FBgn0034965	**0.495**		
CG31619	FBgn0051619	**0.495**	**2.82**	
CG12391	FBgn0033581	**0.494**		
Nap1	FBgn0015268	**0.494**		
Gpo-1	FBgn0022160	**0.493**	**3.37**	**3.32**
Doc3	FBgn0035954	**0.490**		
HDC14725		**0.488**		
CG10433	FBgn0034638	**0.487**	**3.25**	**3.05**
CG8765	FBgn0036900	**0.487**		
HDC16707		**0.485**		
cactin	FBgn0031114	**0.484**	**2.77**	
Marcal1	FBgn0031655	**0.484**		
lute	FBgn0262871	**0.484**	**0.286**	
CG32625	FBgn0052625	**0.482**	**39.17**	
ham	FBgn0045852	**0.480**		
CG7900	FBgn0037548	**0.476**	**2.96**	**2.71**
CG7120	FBgn0035888	**0.475**		
Gs2	FBgn0001145	**0.474**	**3.09**	
Cdep	FBgn0265082	**0.472**	**0.481**	
mgl	FBgn0261260	**0.471**		
Arpc3B	FBgn0065032	**0.470**	**30.56**	
Sp7	FBgn0037515	**0.466**		**0.401**
CG31997	FBgn0051997	**0.465**	**3.22**	
Aats-val	FBgn0027079	**0.465**		
Eip55E	FBgn0000566	**0.463**		
Aats-asn	FBgn0086443	**0.456**		
CG40131	FBgn0058131	**0.454**		
CG34228	FBgn0085257	**0.454**		
CG18747	FBgn0042104	**0.453**		
blanks	FBgn0035608	**0.453**	**98.58**	**0.321**
CG44251	FBgn0265186	**0.450**	**2.21**	

PGRP-SA	FBgn0030310	**0.449**	**2.20**	
squ	FBgn0267347	**0.448**		
CG12288	FBgn0032620	**0.447**	**5.29**	
CG42724	FBgn0261641	**0.443**		
Taf2	FBgn0011836	**0.443**		
Nop60B	FBgn0259937	**0.443**	**3.70**	
gce	FBgn0261703	**0.443**		
CG1503	FBgn0031157	**0.443**		
CG10089	FBgn0036369	**0.436**	**13.85**	
Ssk	FBgn0036945	**0.431**		
Ac3	FBgn0023416	**0.430**		
r-l	FBgn0003257	**0.430**		
CG32088	FBgn0052088	**0.430**		
CG14798	FBgn0029588	**0.428**	**3.41**	
sgll	FBgn0051472	**0.424**		
CG32795	FBgn0040384	**0.422**	**0.468**	
png	FBgn0000826	**0.420**		
CG1550	FBgn0033225	**0.419**		
CG30339	FBgn0050339	**0.417**		
CG18643	FBgn0037898	**0.415**		
CG7458	FBgn0037144	**0.413**		**2.06**
CG2709	FBgn0024977	**0.411**		
Aats-trp	FBgn0010803	**0.411**	**0.171**	
CG9902	FBgn0030757	**0.411**		
CG6912	FBgn0038290	**0.408**		
Gip	FBgn0011770	**0.407**		
CG30340	FBgn0050340	**0.406**		
sage	FBgn0037672	**0.404**	**0.183**	
HDC17231		**0.399**		
CG32017	FBgn0052017	**0.396**	**5.13**	
Cyt-b5-r	FBgn0000406	**0.393**	**10.17**	**2.57**
Mal-B2	FBgn0032382	**0.391**		
CG8613	FBgn0033924	**0.390**		
f-cup	FBgn0028487	**0.385**		
CG31769	FBgn0051769	**0.380**	**2.49**	**2.33**
trk	FBgn0003751	**0.380**	**4.61**	
CG32196	FBgn0052196	**0.378**		
CG1674	FBgn0039897	**0.378**		
GstO1	FBgn0035907	**0.377**	**6.65**	
CG4793	FBgn0028514	**0.374**	**15.37**	
alpha-Est8	FBgn0015576	**0.374**		**2.83**
Bx	FBgn0265598	**0.373**	**2.05**	
CG14545	FBgn0040602	**0.372**	**157.17**	
CG14615	FBgn0031184	**0.370**	**2.16**	
CG4587	FBgn0028863	**0.370**		
CG15100	FBgn0034401	**0.362**		
nonA-l	FBgn0015520	**0.353**		
CG30010	FBgn0050010	**0.352**		
CG18586	FBgn0035642	**0.347**		
CG1677	FBgn0029941	**0.346**		
CT32987		**0.346**		
CG9305	FBgn0032512	**0.342**		

CG10063	FBgn0035727	**0.335**		
CG5568	FBgn0035641	**0.334**		
fend	FBgn0030090	**0.324**	**3.52**	
CG15820	FBgn0035312	**0.322**		
Vago	FBgn0030262	**0.322**	**2.46**	
Sox102F	FBgn0039938	**0.321**		
CG9837	FBgn0037635	**0.320**		**0.441**
aub	FBgn0000146	**0.319**		
ham	FBgn0045852	**0.301**		
CG16888	FBgn0032533	**0.301**		
blot	FBgn0027660	**0.298**	**0.273**	
TfIIFalpha	FBgn0010282	**0.292**		
CG31619	FBgn0051619	**0.291**		
CG5285	FBgn0038490	**0.291**	**2.33**	
betaTub97EF	FBgn0003890	**0.284**		
CG17928	FBgn0032603	**0.280**	**2.79**	
stg1	FBgn0064123	**0.270**		
CG34330	FBgn0085359	**0.260**	**4.33**	
CG32582	FBgn0052582	**0.252**		
AGO3	FBgn0250816	**0.250**	**11.47**	
GstE13	FBgn0033381	**0.246**		
CG8336	FBgn0036020	**0.244**		
CG43103	FBgn0262563	**0.239**	**2.59**	
veil	FBgn0034225	**0.232**		
CG5144	FBgn0035957	**0.223**		
CG3457	FBgn0024984	**0.220**		
piwi	FBgn0004872	**0.209**		
CG10581	FBgn0037046	**0.206**	**5.98**	
CG4872	FBgn0030799	**0.183**	**10.25**	
Nxf3	FBgn0263232	**0.183**		
Ugt36Bc	FBgn0040260	**0.181**		
CG4210	FBgn0038302	**0.169**	**2.30**	
Nrg	FBgn0264975	**0.144**		
CG13602	FBgn0264740	**0.130**		
Iswi	FBgn0011604	**0.126**		
Nrg	FBgn0264975	**0.113**		
eIF4E-6	FBgn0039622	**0.112**		
fu12	FBgn0026718	**0.101**	**3.56**	
CG5367	FBgn0032228	**0.053**		

Transcripts that are up- or down-regulated by RNAi-mediated depletion of ISWI in SL2 cells and H1 in L3 salivary glands or a homozygous null mutation of *Chd1* in L3 larvae. Fold change is calculated from Affymetrix microarray data relative to wild-type control.

SUPPORTING REFERENCES

Aoki, T., Sarkeshik, A., Yates, J., and P. Schedl, 2012 Elba, a novel developmentally regulated chromatin boundary factor is a hetero-tripartite DNA binding complex. *eLife* **1**: e00171.

Arancio, W., Onorati, M.C., Burgio, G., Collesano, M., A.M. Ingrassia *et al*., 2010 The nucleosome remodeling factor ISWI functionally interacts with an evolutionarily conserved network of cellular factors. *Genetics* **185**: 129-140.

Ayyar, S., Jiang, J., Collu, A., White-Cooper, H., and R.A. White, 2003 *Drosophila* TGIF is essential for developmentally regulated transcription in spermatogenesis. *Development* **130**: 2841-2852.

Baffet, A.D., Benoit, B., Januschke, J., Audo, J., V. Gourhand *et al*., 2012 *Drosophila* tubulin-binding cofactor B is required for microtubule network formation and for cell polarity. *Mol Biol Cell* **23**: 3591-3601.

Bateman, J., Shu, H., and D. Van Vactor, 2000. The guanine nucleotide exchange factor trio mediates axonal development in the *Drosophila* embryo. *Neuron* **26**: 93-106.

Berkey, C.D., Blow, N., and P.I. Watnick 2009 Genetic analysis of *Drosophila melanogaster* susceptibility to intestinal *Vibrio cholerae* infection. *Cell Microbiol* **11**: 461-474.

Bhat, K.M., and P. Schedl, 1997 Requirement for *engrailed* and *invected* genes reveals novel regulatory interactions between *engrailed*/*invected*, *patched*, *gooseberry* and *wingless* during *Drosophila* neurogenesis. *Development* **124**: 1675-1688.

Blanke, S., and H. Jackle, 2006 Novel guanine nucleotide exchange factor GEFmeso of *Drosophila melanogaster* interacts with Ral and Rho GTPase Cdc42. *FASEB J* **20**: 683-691.

Bobak, D.A., Nightingale, M.S., Murtagh, J.J., Price, S.R., J. Moss *et al*., 1989 Molecular cloning, characterization, and expression of human ADP-ribosylation factors: two guanine nucleotide-dependent activators of cholera toxin. *Proc Natl Acad Sci U S A* **86**: 6101-6105.

Chabu, C., and C.Q. Doe, 2008 Dap160/intersectin binds and activates aPKC to regulate cell polarity and cell cycle progression. *Development* **135**: 2739-2746.

Chang, H.C., Solomon, N.M., Wassarman, D.A., Karim, F.D., M. Therrien *et al*., 1995 *phyllopod* functions in the fate determination of a subset of photoreceptors in *Drosophila*. *Cell* **80**: 463-472.

Clouse, K.N., Ferguson, S.B., and T. Schupbach, 2008 Squid, Cup, and PABP55B function together to regulate gurken translation in *Drosophila*. *Dev Biol* **313**: 713-724.

Ding, Z.Y., Wang, Y.H., Luo, Z.K., Lee, H.F., J. Hwang *et al*., 2011 Glial cell adhesive molecule unzipped mediates axon guidance in *Drosophila*. *Dev Dyn* **240**: 122-134.

Dubnau, J., Chiang, A.S., Grady, L., Barditch, J., S. Gossweiler *et al*., 2003 The *staufen/pumilio* pathway is involved in *Drosophila* long-term memory. *Curr Biol* **13**: 286-296.

Dyer, N., Rebollo, E., Dominguez, P., Elkhatib, N., P. Chavrier P, *et al*., 2007 Spermatocyte cytokinesis requires rapid membrane addition mediated by ARF6 on central spindle recycling endosomes. *Development* **134**: 4437-4447.

Feng, Z., Liu, H., Lang, J., Li, Y., M. Shu *et al*., 2009 SK66-his, a novel glycine-rich peptide derived from *Drosophila* with antibacterial activity. *Biosci Biotech Biochem* **73**: 769-771.

Giniger, E., Tietje, K., Jan, L.Y., and Y.N. Jan, 1994 *lola* encodes a putative transcription factor required for axon growth and guidance in *Drosophila*. *Development* **120**: 1385-1398.

Goldstein, L.S., and S. Gunawardena, 2000 Flying through the *Drosophila* cytoskeletal genome. *J Cell Biol* **150**: F63-68.

Grimaud, C., Bantignies, F., Pal-Bhadra, M., Ghana, P., U. Bhadra *et al*., 2006 RNAi components are required for nuclear clustering of Polycomb group response elements. *Cell* **124**: 957-971.

Guntermann, S., Primrose, D.A., and E. Foley, 2009 Dnr1-dependent regulation of the *Drosophila* immune deficiency signaling pathway. *Dev Comp Immunol* **33**: 127-134.

Hasegawa, E., Kaido, M., Takayama, R., and M. Sato, 2013 Brain-specific-homeobox is required for the specification of neuronal types in the *Drosophila* optic lobe. *Dev Biol* **377**: 90-99.

Heidmann, D., Horn, S., Heidmann, S., Schleiffer, A., K. Nasmyth *et al*., 2004 The *Drosophila* meiotic kleisin C(2)M functions before the meiotic divisions. *Chromosoma* **113**: 177-187.

Hughes, J.R., Meireles, A.M., Fisher, K.H., Garcia, A., P.R. Antrobus *et al*., 2008 A microtubule interactome: complexes with roles in cell cycle and mitosis. *PLoS Biol* **6**: e98.

Hyman, C.A., Bartholin, L., Newfeld, S.J., and D. Wotton, 2003 *Drosophila* TGIF proteins are transcriptional activators. *Mol Cell Biol* **23**: 9262-9274.

Issigonis, M., Tulina, N., de Cuevas, M., Brawley, C., L. Sandler *et al*., 2009 JAK-STAT signal inhibition regulates competition in the *Drosophila* testis stem cell niche. *Science* **326**: 153-156.

Jafari, S., Alkhori, L., Schleiffer, A., Brochtrup, A., T. Hummel *et al*., 2012 Combinatorial activation and repression by seven transcription factors specify *Drosophila* odorant receptor expression. *PLoS Biol* **10**: e1001280.

James, R.E., and H.T. Broihier, 2011 Crimpy inhibits the BMP homolog Gbb in motoneurons to enable proper growth control at the *Drosophila* neuromuscular junction. *Development* **138**: 3273-3286.

Jin, L.H., Shim, J., Yoon, J.S., Kim, B., J. Kim *et al*., 2008 Identification and functional analysis of antifungal immune response genes in *Drosophila*. *PLoS Pathogens* **4**: e1000168.

Johnstone, O., and P. Lasko, 2001 Translational regulation and RNA localization in *Drosophila* oocytes and embryos. *Ann Rev Genet* **35**: 365-406.
Kirilly, D., Spana, E.P., Perrimon, N., Padgett, R.W., and T. Xie, 2005 BMP signaling is required for controlling somatic stem cell self-renewal in the *Drosophila* ovary. *Dev Cell* **9**: 651-662.
Kleino, A., Valanne, S., Ulvila, J., Kallio, J., H. Myllymaki *et al*., 2005 Inhibitor of apoptosis 2 and TAK1-binding protein are components of the *Drosophila* Imd pathway. *EMBO J* **24**: 3423-3434.
Koh, T.W., Verstreken, P., and H.J. Bellen, 2004 Dap160/intersectin acts as a stabilizing scaffold required for synaptic development and vesicle endocytosis. *Neuron* **43**: 193-205.
Konev, A.Y., Tribus, M., Park, S.Y., Podhraski, V., C.Y. Lim *et al*., 2007 CHD1 motor protein is required for deposition of histone variant H3.3 into chromatin in vivo. *Science* **317**: 1087-1090.
Kraut, R., and K. Zinn, 2004 Roundabout 2 regulates migration of sensory neurons by signaling in trans. *Curr Biol* **14**: 1319-1329.
Krueger, N.X., Reddy, R.S., Johnson, K., Bateman, J., N. Kaufmann *et al*., 2003 Functions of the ectodomain and cytoplasmic tyrosine phosphatase domains of receptor protein tyrosine phosphatase Dlar in vivo. *Mol Cell Biol* **23**: 6909-6921.
Krueger, N.X., Van Vactor, D., Wan, H.I., Gelbart, W.M., G.S. Goodman *et al*., 1996 The transmembrane tyrosine phosphatase DLAR controls motor axon guidance in *Drosophila*. *Cell* **84**: 611-622.
Kusch, T., Florens, L., Macdonald, W.H., Swanson, S.K., R.L. Glaser *et al*., 2004 Acetylation by Tip60 is required for selective histone variant exchange at DNA lesions. *Science* **306**: 2084-2087.
Laflamme, C., Assaker, G., Ramel, D., Dorn, J.F., D. She *et al*., 2012 Evi5 promotes collective cell migration through its Rab-GAP activity. *J Cell Biol* **198**: 57-67.
Lee, S.Y., Lee, S.Y., and Y. Choi, 1997 TRAF-interacting protein (TRIP): a novel component of the tumor necrosis factor receptor (TNFR)- and CD30-TRAF signaling complexes that inhibits TRAF2-mediated NF-kappaB activation. *J Exp Med* **185**: 1275-1285.
Lloyd, T.E., Verstreken, P., Ostrin, E.J., Phillippi, A., O. Lichtarge *et al*., 2000 A genome-wide search for synaptic vesicle cycle proteins in *Drosophila*. *Neuron* **26**: 45-50.
Manser, E., Loo, T.H., Koh, C.G., Zhao, Z.S., X.Q. Chen *et al*., 1998 PAK kinases are directly coupled to the PIX family of nucleotide exchange factors. *Mol Cell* **1**: 183-192.
McDaniel, I.E., Lee, J.M., Berger, M.S., Hanagami, C.K., and J.A. Armstrong, 2008 Investigations of CHD1 function in transcription and development of *Drosophila melanogaster*. *Genetics* **178**: 583-587.
Merkle, J.A., Rickmyre, J.L., Garg, A., Loggins, E.B., J.N. Jodoin *et al*., 2009 *no poles* encodes a predicted E3 ubiquitin ligase required for early embryonic development of *Drosophila*. *Development* **136**: 449-459.
Morante, J., Vallejo, D.M., Desplan, C., and M. Dominguez, 2013 Conserved miR-8/miR-200 defines a glial niche that controls neuroepithelial expansion and neuroblast transition. *Dev Cell* **27**: 174-187.
Nagaraj, R., and U. Banerjee, 2009 Regulation of Notch and Wingless signalling by *phyllopod*, a transcriptional target of the EGFR pathway. *EMBO J* **28**: 337-346.
Neumuller, R.A., Richter, C., Fischer, A., Novatchkova, M., K.G. Neumuller *et al*., 2011 Genome-wide analysis of self-renewal in *Drosophila* neural stem cells by transgenic RNAi. *Cell Stem Cell* **8**: 580-593.
O'Neill, R.S., and D.V. Clark, 2013 The *Drosophila* melanogaster septin gene Sep2 has a redundant function with the retrogene Sep5 in imaginal cell proliferation but is essential for oogenesis. *Genome* **56**: 753-758.
Parnas, D., Haghighi, A.P., Fetter, R.D., Kim, S.W., and C.S. Goodman, 2001 Regulation of postsynaptic structure and protein localization by the Rho-type guanine nucleotide exchange factor dPix. *Neuron* **32**: 415-424.
Parrish, J.Z., Kim, M.D., Jan, L.Y., and Y.N. Jan, 2006 Genome-wide analyses identify transcription factors required for proper morphogenesis of *Drosophila* sensory neuron dendrites. *Genes Dev* **20**: 820-835.
Pertceva, J.A., Dorogova, N.V., Bolobolova, E.U., Nerusheva, O.O., S.A. Fedorova *et al*., 2010 The role of *Drosophila* hyperplastic discs gene in spermatogenesis. *Cell Biol Int* **34**: 991-996.
Rahn, T., Leippe, M., Roeder, T., and H. Fedders, 2013 EGFR signaling in the brain is necessary for olfactory learning in *Drosophila* larvae. *Learm Mem* **20**: 194-200.
Simmonds, A.J., Brook, W.J., Cohen, S.M., and J.B. Bell, 1995 Distinguishable functions for *engrailed* and *invected* in anterior-posterior patterning in the *Drosophila* wing. *Nature* **376**: 424-427.
Simpson, J.H., Kidd, T., Bland, K.S., and C.S. Goodman, 2000 Short-range and long-range guidance by slit and its Robo receptors. Robo and Robo2 play distinct roles in midline guidance. *Neuron* **28**: 753-766.
Sinclair, D.A., Clegg, N.J., Antonchuk, J., Milne, T.A., K. Stankunas *et al*., 1998 *Enhancer of Polycomb* is a suppressor of position-effect variegation in *Drosophila melanogaster*. *Genetics* **148**: 211-220.
Singh, S.R., Zheng, Z., Wang, H., Oh, S.W., X. Chen *et al*., 2010 Competitiveness for the niche and mutual dependence of the germline and somatic stem cells in the *Drosophila* testis are regulated by the JAK/STAT signaling. *J Cell Physiol* **223**: 500-510.
Stankunas, K., Berger, J., Ruse, C., Sinclair, D.A., F. Randazzo *et al*., 1998 The *Enhancer of Polycomb* gene of *Drosophila* encodes a chromatin protein conserved in yeast and mammals. *Development* **125**: 4055-4066.

Tea, J.S., and L. Luo, 2011 The chromatin remodeling factor Bap55 functions through the TIP60 complex to regulate olfactory projection neuron dendrite targeting. *Neur Dev* **6**: 5.
Terman, J.R., and A.L. Kolodkin, 2004 Nervy links protein kinase a to plexin-mediated semaphorin repulsion. *Science* **303**: 1204-1207.
Tsuzuki, S., Ochiai, M., Matsumoto, H., Kurata, S., A. Ohnishi *et al*., 2012 *Drosophila* growth-blocking peptide-like factor mediates acute immune reactions during infectious and non-infectious stress. *Sci Rep* **2**: 210.
Wallace, H.A., Merkle, J.A., Yu, M.C., Berg, T.G., E. Lee *et al*., 2014 TRIP/NOPO E3 ubiquitin ligase promotes ubiquitylation of DNA polymerase eta. *Development* **141**: 1332-1341.
Wang, X., Shaw, W.R., Tsang, H.T., Reid, E., and C.J. O'Kane, 2007 *Drosophila* spichthyin inhibits BMP signaling and regulates synaptic growth and axonal microtubules. *Nat Neurosci* **10**: 177-185.
Williams, R.W., and G.M. Rubin, 2002 ARGONAUTE1 is required for efficient RNA interference in *Drosophila* embryos. *Proc Natl Acad Sci U S A* **99**: 6889-6894.
Xu, N., Emelyanov, A.V., Fyodorov, D.V., and A.I. Skoultchi, 2014 *Drosophila* linker histone H1 coordinates STAT-dependent organization of heterochromatin and suppresses tumorigenesis caused by hyperactive JAK-STAT signaling. *Epigenetics Chromatin* **7**: 16.
Yamaguchi, S., Katagiri, S., Sekimizu, K., Natori, S., and K.J. Homma, 2005 Involvement of EDTP, an egg-derived tyrosine phosphatase, in the early development of *Drosophila melanogaster*. *J Biochem* **138**: 721-728.
Yamamoto, A., Zwarts, L., Callaerts, P., Norga, K., T.F. Mackay *et al*., 2008 Neurogenetic networks for startle-induced locomotion in *Drosophila melanogaster*. *Proc Natl Acad Sci U S A* **105**: 12393-12398.
Yang, H., Liu, C., Jamsen, J., Wu, Z., Y. Wang *et al*., 2012 The DNase domain-containing protein TATDN1 plays an important role in chromosomal segregation and cell cycle progression during zebrafish eye development. *Cell Cycle* **11**: 4626-4632.
Yang, L., Chen, D., Duan, R., Xia, L., J. Wang *et al*., 2007 Argonaute 1 regulates the fate of germline stem cells in *Drosophila*. *Development* **134**: 4265-4272.
Zheng, L., and R.W. Carthew, 2008 Lola regulates cell fate by antagonizing Notch induction in the *Drosophila* eye. *Mech Dev* **125**: 18-29.
