# Supplementary material for: A Genetic Screen and Transcript Profiling Reveal a Shared Regulatory Program for Drosophila Linker Histone H1 and Chromatin Remodeler CHD1
Source: G3 (Bethesda). 2015 Jan 27;5(4):677–87. doi: 10.1534/g3.115.016709 (PMC4390582; doi:10.1534/g3.115.016709)
Supplement: Supporting Information [file supp_g3.115.016709_TableS1.pdf]

**Table S1 GO term analyses of genetic modifiers of *His1* effect on viability.**

| Gene Symbol | Biological evidence for the gene and/or gene product | Reference(s) |
|---|---|---|
| **Chromosome organization (biological function), GO:0051276** | | |
| *nopo* | Fly homolog of human E3 ligase TRIP; interacts with DNA polymerase η, regulates S to M transition during syncytial nuclear divisions. | (Wallace et al. 2014)<br>(Merkle et al. 2009) |
| *AGO1* | Component of miRNA-specific silencing machinery; was shown to regulate nuclear organization of PcG target loci. | (Williams and Rubin 2002)<br>(Grimaud et al. 2006) |
| *CG3358* | Fly ortholog of TatD-related DNAse TATDN1 that plays an important role in mitotic chromosome segregation. | (Yang et al. 2012) |
| *Sep5* | Nuclear GTPase and a component of microtubule-associated complex involved in cytokinesis. | (Hughes et al. 2008)<br>(Goldstein and Gunawardena 2000) |
| *E(Pc)* | Contributes to chromatin-dependent silencing; is not present in PRC1 or PRC2 but, rather, is a subunit of histone acetyltranferase complex TIP60 involved in both gene activation and repression; interacts with *ISWI*, which is involved in H1 deposition into chromatin. | (Sinclair et al. 1998)<br>(Stankunas et al. 1998)<br>(Kusch et al. 2004)<br>(Arancio et al. 2010) |
| *Elba2* | BEN domain-containing protein; associates with the *Fab-7* boundary/insulator element, promotes chromatin silencing. | (Aoki et al. 2012) |
| *Socs36E* | SH2 domain-containing suppressor of cytokine signaling; a negative regulator of the JAK/STAT cascade, which is involved, together with H1, in the establishment of heterochromatin silencing. | (Singh et al. 2010)<br>(Xu et al. 2014) |
| *c(2)M* | Component of the synaptonemal complex; required for reciprocal meiotic recombination. | (Heidmann et al. 2004) |
| *Chd1* | ATP-dependent nucleosome remodeling motor protein; essential for chromatin assembly in the nascent male pronucleus and for deposition of H3.3 at transcriptionally active loci. | (Konev et al. 2007) |
| **DNA binding (molecular function), GO:0003677** | | |
| *bsh* | Brain-specific homeobox protein. | (Hasegawa et al. 2013) |
| *vis* | Homeobox-containing TGIF-related transactivator. | (Hyman et al. 2003) |
| *inv* | Helix-turn-helix transcription factor. | (Simmonds et al. 1995) |
| *zf30C* | Zinc finger transcription factor. | (Jafari et al. 2012) |
| *CG3358* | Fly ortholog of TatD-related DNAse TATDN1. | (Yang et al. 2012) |
| *CG12744* | Zinc finger transcription factor. | (Jin et al. 2008) |
| *lola* | Zinc finger transcription factor. | (Giniger et al. 1994) |
| *Elba2* | BEN domain-containing protein, associates with the *Fab-7* element. | (Aoki et al. 2012) |
| *Chd1* | ATP-dependent nucleosome remodeling motor protein. | (Konev et al. 2007) |
| *c(2)M* | Component of the synaptonemal complex. | (Heidmann et al. 2004) |
| **Small GTPase-mediated signal transduction (biological function), GO:0007264** | | |
| *phyl* | Regulator of Notch and Wnt signaling pathways. | (Nagaraj and Banerjee 2009) |
| *CG15611* | Exhibits sequence similarity to fly Rho guanyl-nucleotide exchange factor TRIO. | (Bateman et al. 2000) |
| *CG8155* | Structurally similar to fly Rab GTPase activator Evi5. | (Laflamme et al. 2012) |
| *GEFmeso* | Mesoderm guanine nucleotide exchange, a binding target of Ras-like GTPase Ral. | (Blanke and Jackle 2006) |
| *RtGEF* | Homologous to mammalian Rho-type guanyl-nucleotide exchange factor Pix. | (Manser et al. 1998) |
| *Arf51F* | GTP-binding protein according to sequence similarity to human ADP-ribosylation factor 2, Arf2. | (Bobak et al. 1989) |
| *Sep5* | Belongs to the superfamily of Septin GTPases. | (O'Neill and Clark 2013) |
| **Gametogenesis (biological function), GO:0048477, GO:0007283** | | |
| *gbb* | Fly ortholog of human bone morphogenic protein-5 (BMP-5); was shown to be involved in BMP signaling that controls ovarian cell development. | (Kirilly et al. 2005) |
| *vis* | Transcription factor; required for regulation of multiple genes involved in sperm manufacture; mutants are male sterile due to a defect in primary | (Ayyar et al. 2003) |

| | | |
|---|---|---|
| | spermatocyte differentiation before the onset of meiotic divisions. | |
| *TBCB* | Tubulin-binding cofactor B, a ubiquitin-related domain-containing protein; regulates microtubule organization in the nucleus and controls oocyte polarity. | (Baffet et al. 2012) |
| *AGO1* | Plays central roles in female germ-line cell differentiation. | (Yang et al. 2007) |
| *Arf51F* | ADP ribosylation factor; is required for cytokinesis in spermatocytes. | (Dyer et al. 2007) |
| *stau* | dsRNA- and mRNA 3'-UTR-binding protein; regulates translation and mRNA localization of egg polarity genes *bicoid*, *gurken*, *nanos* and *oskar*. | (Johnstone and Lasko 2001) |
| *Lar* | Leukocyte antigen-related-like tyrosine phosphatase receptor protein; required for the establishment of cell polarity during oogenesis. | (Krueger et al. 2003) |
| *Edtp* | Egg-derived tyrosine phosphatase; essential for ovarian development, oogenesis and embryogenesis. | (Yamaguchi et al. 2005) |
| *Socs36E* | Functions in male germ-line stem cell maintenance. | (Issigonis et al. 2009)<br>(Singh et al. 2010) |
| *pAbp* | Polyadenylate-binding protein; essential for spermatid elongation; translationally regulates Grk (*gurken*), which is required for the establishment of the dorsal-ventral axis of a developing egg. | (Pertceva et al. 2010)<br>(Clouse et al. 2008) |
| *Chd1* | SNF2-like ATPase; functions in oogenesis and egg fertilization. | (Konev et al. 2007)<br>(McDaniel et al. 2008) |
| **Nervous system development and neural system processes (biological function), GO:0007399, GO:0050877** | | |
| *Dap160* | Dynamin-associated protein, contains EF-hand and SH3 domains, a negative regulator of Notch signaling; interacts with the atypical protein kinase C (aPKC) to stimulate neuroblast proliferation; similar to dynamin, regulates synaptic vesicle endocytosis. | (Chabu and Doe 2008)<br>(Koh et al. 2004) |
| *bsh* | Transcription factor; regulates neuronal identity specification. | (Hasegawa et al. 2013) |
| *phyl* | Zinc-binding protein; essential for the peripheral nervous system development and photoreceptor differentiation. | (Chang et al. 1995) |
| *gbb* | Part of the BMP signaling pathway; important for the neuromuscular junction development and synaptic transmission. | (James and Broihier 2011) |
| *CG5742* | Predicted ankyrin repeat-containing protein; was found to regulate neurogenesis in an RNAi screen in vivo in *Drosophila*. | (Neumuller et al. 2011) |
| *inv* | Transcription factor; regulates neuroblast fate determination. | (Bhat and Schedl 1997) |
| *lea* | Axon guidance receptor; regulates neuron migration. | (Simpson et al. 2000)<br>(Kraut and Zinn 2004) |
| *zf30C* | Transcription factor; regulates dendrite morphology in the developing brain. | (Parrish et al. 2006) |
| *spict* | Magnesium transmembrane transporter orthologous to human NIPA1; negatively regulates BMP signaling to promote synaptic growth at the neuromuscular junction. | (Wang et al. 2007) |
| *Pka-R2* | cAMP-dependent protein kinase R2; functions in repulsive axon guidance. | (Terman and Kolodkin 2004) |
| *spi* | TGF-$\alpha$-like ligand that triggers epidermal growth factor receptor (EGFR) activation to promote neuroepithelial proliferation and neuroblast formation in the optic lobe; as a component of EGFR signaling pathway, is important for olfactory learning in flies. | (Morante et al. 2013)<br>(Rahn et al. 2013) |
| *RtGEF* | Rho-type guanine nucleotide exchange factor; regulates postsynaptic structure and protein localization at the glutaminergic neuromuscular junction. | (Parnas et al. 2001) |
| *uzip* | Cell adhesion protein; functions in axon guidance in the *Drosophila* central nervous system. | (Ding et al. 2011) |
| *Arf51F* | GTP-binding protein; has a regulatory function in synaptic vesicle endocytosis. | (Lloyd et al. 2000) |
| *stau* | Regulator of translation, a major player in the establishment of long-term memory and neuronal plasticity. | (Dubnau et al. 2003) |
| *lola* | Transcription factor; regulates axonogenesis, brain morphogenesis and eye development. | (Giniger et al. 1994)<br>(Yamamoto et al. 2008)<br>(Zheng and Carthew 2008) |

| | | |
|---|---|---|
| *Lar* | Tyrosine phosphatase receptor; plays a key role in the control of axon guidance. | (Krueger et al. 1996) |
| *E(Pc)* | Together with other subunits of the TIP60 HAT complex regulates dendrite targeting of olfactory neurons. | (Tea and Luo 2011) |
| **Stress response (biological function), GO:0006955, GO:0006952** | | |
| *nopo* | E3 ligase; likely plays a role in defense response, based on its homology with human TRAF-interacting protein TRIP. | (Lee et al. 1997) |
| *coro* | WD40 repeat-containing protein; belongs to a family of proteins involved in defense response to fungus. | (Jin et al. 2008) |
| *Gbp* | Growth blocking peptide; a potent cytokine that stimulates the expression of anti-microbial peptides. | (Tsuzuki et al. 2012) |
| *lola* | Transcription factor; functions in anti-microbial humoral response. | (Kleino et al. 2005) |
| *CG13551* | ATPase inhibitor, glycine-rich protein; has an in vitro activity in suppressing Gramm-positive bacteria. | (Feng et al. 2009) |
| *dnr1* | FERM domain- and RING finger-containing protein; an inhibitor of the immune deficiency (IMD) pathway at the level of initiator caspase Dredd. | (Guntermann et al. 2009) |
| *Jhl-21* | Leucine transmembrane transporter; belongs to a family of proteins involved in defense response to fungus. | (Jin et al. 2008) |
| *Traf4* | TNF receptor-associated factor; functions in the IMD pathway and plays a role in fly defense response to *Vibrio cholera*. | (Berkey et al. 2009) |

The known attributes and properties of genetic suppressors and enhancers of *His1* function in vivo that belong to enriched GO term classes are listed.
