# Supplementary material for: A Genetic Screen and Transcript Profiling Reveal a Shared Regulatory Program for Drosophila Linker Histone H1 and Chromatin Remodeler CHD1
Source: G3 (Bethesda). 2015 Jan 27;5(4):677–87. doi: 10.1534/g3.115.016709 (PMC4390582; doi:10.1534/g3.115.016709)
Supplement: Supporting Information [file supp_g3.115.016709_TableS2.pdf]

Table S2 Overlap of transcripts regulated by CHD1, H1, HP1 and ISWI.

SYMBOL/ TRANSCRIPT	FLYBASE ID	FOLD CHANGE			
		Chd1	**H1 KD**	**HP1 KD**	**ISWI KD**
Chd1* effect: *UP					
**detoxification**					
Gclc	FBgn0040319	**2.04**			
GstT4	FBgn0030484	**2.11**	**2.14**	**2.28**	
Cyp12b2	FBgn0034387	**2.22**			
Cyp4ac3	FBgn0031695	**2.25**	**29.44**		
Cyp18a1	FBgn0010383	**2.28**			
Cyp28a5	FBgn0028940	**2.30**	**2.27**		
Cyt-b5-r	FBgn0000406	**2.57**	**10.17**		**0.393**
GstD6	FBgn0010042	**2.68**	**13.24**		
CG4623	FBgn0035587	**3.16**			
Cyp9c1	FBgn0015040	**3.41**		**6.66**	
Cyp6a18	FBgn0039519	**3.81**			
Ugt86Dj	FBgn0040250	**3.92**			
Cyp9h1	FBgn0033775	**4.06**			
GstE6	FBgn0063494	**4.17**	**43.50**	**2.95**	
Cyp313a3	FBgn0038007	**5.03**			
Cyp6a9	FBgn0013771	**5.28**	**2.30**		
Cyp4d8	FBgn0015033	**5.74**	**2.40**		
Cyp6a23	FBgn0033978	**5.98**	**16.25**		**3.07**
GstE7	FBgn0063493	**8.94**		**3.78**	
GstE5	FBgn0063495	**12.3**	**29.07**		
Cyp6a2	FBgn0000473	**30.5**		**2.21**	
Cyp4p2	FBgn0033395	**40.2**	**4.52**		
Cyp4e3	FBgn0015035	**73.0**	**7.07**		
**development**					
Iris	FBgn0031305	**2.00**			
Idgf3	FBgn0020414	**2.00**	**3.07**		
hts	FBgn0263391	**2.04**			
CG13461	FBgn0036468	**2.14**	**5.69**		
Rbp9	FBgn0010263	**2.23**	**2.02**		
rad50	FBgn0034728	**2.27**	**7.96**		
PH4alphaSG2	FBgn0039779	**2.35**			
mbl	FBgn0265487	**2.45**			
CR_TC_RE65113		**2.51**			
CG42390	FBgn0259736	**2.51**	**0.464**		**2.48**
CG15784	FBgn0029766	**2.68**	**3.42**		
Npc1b	FBgn0261675	**2.77**			
bol	FBgn0011206	**2.91**			
Glycogenin	FBgn0265191	**2.95**			
lola	FBgn0005630	**3.12**			
CG14265	FBgn0040393	**3.36**			
chinmo	FBgn0086758	**3.46**			
ine	FBgn0011603	**3.51**	**0.062**		
sens	FBgn0002573	**3.58**			
Atf3	FBgn0028550	**3.81**			
JhI-26	FBgn0028424	**3.84**	**7.00**	**2.68**	
metro	FBgn0050021	**3.86**	**0.084**		

CG5867	FBgn0027586	**3.97**			
Est-Q	FBgn0037090	**4.06**	**5.17**		
Ftz-f1	FBcl0153222	**4.06**			
Dh44-R2	FBgn0033744	**4.23**	**2.33**		
chinmo	FBgn0086758	**4.35**			
CG10918	FBgn0031178	**4.76**			
smp-30	FBgn0038257	**4.76**	**19.82**		
tx	FBgn0263118	**4.86**			
fru	FBgn0004652	**5.13**	**2.85**		
smp-30	FBgn0038257	**5.46**	**19.82**		
CG32694	FBgn0052694	**5.54**			
Msp300	FBgn0261836	**5.74**			
lcs	FBgn0028583	**6.87**			
LP09368	FBcl0192001	**7.94**			
CG32071	FBgn0052071	**8.17**			
CG42368	FBgn0259714	**42.2**	**0.471**		
CG16898	FBgn0034480	**69.1**	**6.66**		
l(2)efl	FBgn0011296	**85.6**			
**immune response**					
kek5	FBgn0031016	**2.01**			
Ect4	FBgn0262579	**2.06**		**2.17**	
SPE	FBgn0039102	**2.19**	**4.83**		
Tsp42Eh	FBgn0033129	**2.20**			
Sr-CI	FBgn0014033	**2.46**	**4.37**		
CG8562	FBgn0035779	**2.57**			
dl	FBgn0260632	**2.57**			
ea	FBgn0000533	**2.60**			
Tsp42Eg	FBgn0033128	**2.62**			
ninaD	FBgn0002939	**2.71**			
PebIII	FBgn0011695	**2.85**			
Phae2	FBgn0263235	**2.85**			
alpha-Est2	FBgn0015570	**2.85**	**2.17**		
TBCB	FBgn0034451	**2.97**			
CG10433	FBgn0034638	**3.05**	**3.25**		**0.487**
CG5597	FBgn0034920	**3.07**			
Ect4	FBgn0262579	**3.12**		**2.17**	
NimB1	FBgn0027929	**3.18**			
CG15255	FBgn0028950	**3.46**			
NimC1	FBgn0259896	**3.51**			
CG3775	FBgn0030425	**3.53**			
CG5791	FBgn0040582	**3.58**			
Ser6	FBgn0011834	**3.78**			
CG13947	FBgn0031277	**4.14**	**2.26**		
CG7248	FBgn0036229	**4.23**			
CG5550	FBgn0034160	**4.38**			
PGRP-SC1b	FBgn0033327	**4.47**			
CG15293	FBgn0028526	**4.50**	**5.16**		
CG8620	FBgn0040837	**4.89**	**160.83**		
mthl8	FBgn0052475	**4.99**	**3.29**		
CG17974	FBgn0034624	**5.46**			
spheroide	FBgn0030774	**5.74**			
CG13324	FBgn0033789	**5.78**			

CG9928	FBgn0032472	**5.86**	**19.55**			
TotC	FBgn0044812	**6.15**	**58.64**			
CG18179	FBgn0036023	**6.32**				
Muc68Ca	FBgn0036181	**7.36**			**2.07**	
CG33178	FBgn0053178	**7.36**				
CG5778	FBgn0038930	**7.57**	**27.36**			
Fmo-2	FBgn0033079	**9.00**	**9.77**			
CG14419	FBgn0029639	**12.1**				
CG16775	FBgn0036767	**13.9**				
Prx2540-2	FBgn0033518	**27.1**	**0.483**			
Mdr50	FBgn0010241	**54.6**				
**intracellular processes**						
larp	FBgn0261618	**2.01**				
Sec31	FBgn0033339	**2.01**	**0.483**			
dm	FBgn0262656	**2.03**				
stau	FBgn0003520	**2.06**				
mTTF	FBgn0028530	**2.10**				
qless	FBgn0051005	**2.10**				
Ndae1	FBgn0259111	**2.10**	**0.357**	**4.82**		
Nf1	FBgn0015269	**2.14**				
CG8503	FBgn0033917	**2.23**				
form3	FBgn0053556	**2.28**				
Msp300	FBgn0261836	**2.38**				
CG5599	FBgn0030612	**2.41**				
mbl	FBgn0265487	**2.53**				
CG2681	FBgn0024997	**2.68**				
Irbp	FBgn0011774	**2.71**	**14.23**			
CG3328	FBgn0034985	**2.75**				
GluRIIC	FBgn0046113	**2.75**				
phr	FBgn0003082	**2.79**				
Obp56a	FBgn0034468	**2.79**				
CG5205	FBgn0038344	**2.89**	**2.44**			
CG44085	FBgn0264894	**2.91**				
Diver		**3.03**	**3.62**			
CG30118	FBgn0050118	**3.03**	**2.00**			
CG6503	FBgn0040606	**3.05**	**10.18**			
ng3	FBgn0010295	**3.05**				
zormin	FBgn0052311	**3.34**	**0.456**			
ps	FBgn0261552	**3.34**				
Skeletor	FBgn0262717	**3.48**				
CG14661	FBgn0037288	**3.68**				
CG6910	FBgn0036262	**3.73**	**2.65**			
CG12863	FBgn0033948	**3.78**				
CG30285	FBgn0050285	**4.35**	**4.04**			
CG44013	FBgn0264775	**4.89**				
Ude	FBgn0039226	**5.03**			**3.85**	
CG2120	FBgn0030005	**5.28**				
CG44013	FBgn0264775	**5.46**				
tobi	FBgn0261575	**5.94**				
CG6839	FBgn0036831	**6.06**				
CG7299	FBgn0032282	**6.63**				
CG11659	FBgn0038731	**7.21**				

CG5070	FBgn0030824	**8.69**			
CG9757	FBgn0003060	**16.8**			
**metabolism**					
CG30427	FBgn0043792	**2.00**			
CG7149	FBgn0031948	**2.00**			
Evi5	FBgn0262740	**2.01**			
CG3902	FBgn0036824	**2.03**			
CG11453	FBgn0038734	**2.04**			
CG15534	FBgn0039769	**2.11**			
Marf	FBgn0029870	**2.11**			
CG40486	FBgn0263830	**2.13**			
Unc-115b	FBgn0260463	**2.14**			
CG9674	FBgn0036663	**2.16**			
CG3523	FBgn0027571	**2.16**	**2.37**		
CG6733	FBgn0039052	**2.16**			
su(r)	FBgn0086450	**2.17**	**3.20**	**2.45**	
Men	FBgn0002719	**2.20**	**4.11**		
Amy-p	FBgn0000079	**2.20**			
alpha-Est10	FBgn0015569	**2.22**	**3.40**		
CG43340	FBgn0263077	**2.22**		**3.00**	
CG32645	FBgn0052645	**2.22**			
pgant4	FBgn0051956	**2.22**			
CG17322	FBgn0027070	**2.22**			
CG31475	FBgn0051475	**2.23**			
AdSS	FBgn0027493	**2.23**	**2.01**		
GstZ2	FBgn0037697	**2.25**			
nemy	FBgn0261673	**2.27**			
Ugt86Da	FBgn0040259	**2.28**	**8.97**	**2.71**	
CAHbeta	FBgn0037646	**2.30**			
CG7920	FBgn0039737	**2.30**			
Pepck	FBgn0003067	**2.30**		**4.99**	**2.84**
Spat	FBgn0014031	**2.31**	**2.38**		
Mocs1	FBgn0263241	**2.31**			
CG32649	FBgn0052649	**2.31**			
Pkc53E	FBgn0003091	**2.33**	**0.340**		
CG14655	FBgn0037275	**2.33**			
CG13397	FBgn0014417	**2.33**			
CG8129	FBgn0037684	**2.35**	**2.00**		
Ac78C	FBgn0024150	**2.38**			
Kua	FBgn0032850	**2.38**			
Ca-P60A	FBgn0263006	**2.43**			
CG11162	FBgn0030509	**2.43**			
CG32557	FBgn0052557	**2.46**			
Uro	FBgn0003961	**2.48**	**2.13**		
CG3597	FBgn0031417	**2.48**	**2.08**		
CG3301	FBgn0038878	**2.48**	**0.448**		
Aldh	FBgn0012036	**2.48**	**2.56**		
CG5704	FBgn0026570	**2.50**	**2.10**		
CG1315	FBgn0026565	**2.51**			
Ugt35a	FBgn0026315	**2.53**	**3.35**	**2.47**	
drd	FBgn0260006	**2.55**			
ACC	FBgn0033246	**2.57**	**7.19**		

Tdc1	FBgn0259977	**2.57**			
CG1673	FBgn0030482	**2.58**			
AOX1	FBgn0267408	**2.60**			
Gpdh	FBgn0001128	**2.62**	**2.22**		
CG1774	FBgn0039856	**2.64**			
CG10175	FBgn0039084	**2.66**	**0.481**		
Jheh3	FBgn0034406	**2.68**		**6.01**	
CG10827	FBgn0038845	**2.68**			
Gad1	FBgn0004516	**2.73**			
Est-6	FBgn0000592	**2.77**	**7.32**		
CG33093	FBgn0053093	**2.79**	**7.39**		
CG6074	FBgn0039486	**2.79**	**23.77**		
Tie	FBgn0014073	**2.81**	**0.205**		
CG8128	FBgn0030668	**2.83**			
alpha-Est8	FBgn0015576	**2.83**			**0.374**
CG5707	FBgn0026593	**2.87**			
CG13311	FBgn0035929	**2.89**			
Obp56e	FBgn0034471	**2.89**			
l(1)G0196	FBgn0027279	**2.89**			
CG14787	FBgn0027793	**2.91**			**17.33**
CG3841	FBgn0032131	**2.95**			
CG9993	FBgn0034553	**2.97**			
sls	FBgn0086906	**3.10**	**0.081**		
CG30503	FBgn0050503	**3.14**	**0.392**		**2.60**
CG14762	FBgn0033250	**3.16**	**33.48**		
CG10165	FBgn0032801	**3.16**	**3.66**		
Gbs-76A	FBgn0036862	**3.18**			
Ugt86Dh	FBgn0040252	**3.29**			
CG7025	FBgn0031930	**3.29**			
Cyp18a1	FBgn0010383	**3.32**			
Npc2h	FBgn0039801	**3.32**	**5.23**		
Gpo-1	FBgn0022160	**3.32**	**3.37**		**0.493**
RluA-1	FBgn0051719	**3.34**			
Ork1	FBgn0017561	**3.36**	**11.63**		
Mipp1	FBgn0026061	**3.36**	**4.33**		
CG15879	FBgn0035309	**3.39**			
CG42329	FBgn0259229	**3.41**	**3.51**		
CG9466	FBgn0032068	**3.48**			
CG8708	FBgn0033271	**3.58**	**3.21**		
CG3835	FBgn0023507	**3.58**	**3.69**		
sls	FBgn0086906	**3.63**	**0.081**		
fa2h	FBgn0050502	**3.66**			
CG1461	FBgn0030558	**3.66**			
CG8629	FBgn0035742	**3.89**			
CG10592	FBgn0035619	**3.97**			
CG15117	FBgn0034417	**4.06**			
CG32170	FBgn0052170	**4.06**			
Unc-89	FBgn0053519	**4.06**			
St3	FBgn0265052	**4.08**			
Gpdh	FBgn0001128	**4.29**	**2.22**		
Mlc2	FBgn0002773	**4.29**	**2.31**		
arg	FBgn0023535	**4.35**	**5.01**		

CS-2	FBgn0029091	**4.50**			
CG17843	FBgn0038919	**4.50**			
AOX2	FBgn0038348	**4.69**			
glob1	FBgn0027657	**4.72**	**6.42**		
CG30457	FBgn0050457	**4.79**			
CG9743	FBgn0039756	**5.10**	**2.17**		
CG32751	FBgn0052751	**5.13**			
CG9468	FBgn0032069	**5.13**			
CG4753	FBgn0036622	**5.13**	**8.81**		
CG42249	FBgn0259101	**5.28**			
CG12512	FBgn0031703	**5.31**	**11.53**		
Apoltp	FBgn0032136	**5.70**	**2.89**	**2.39**	**2.22**
CG5150	FBgn0035620	**6.28**			
vanin-like	FBgn0040069	**7.57**			
CG8630	FBgn0038130	**8.22**			
CG3106	FBgn0030148	**8.63**			
CG14022	FBgn0031700	**8.69**			
Odc2	FBgn0013308	**9.45**			
CG14205	FBgn0031034	**13.4**			
frac	FBgn0035798	**20.3**			
Jhedup	FBgn0034076	**21.3**			
CG11796	FBgn0036992	**22.9**			
hgo	FBgn0040211	**23.4**			
CG4716	FBgn0033820	**32.0**	**11.77**		
CG4716	FBgn0033820	**61.8**	**11.77**		
CG4757	FBgn0027584	**190**	**5.27**		
**proteolysis**					
CG9505	FBgn0031805	**2.00**	**2.72**		
CG18417	FBgn0035780	**2.01**			
CG31821	FBgn0051821	**2.08**			
Try29F	FBgn0015316	**2.10**	**2.28**		
CG18493	FBgn0038701	**2.14**			
CG4721	FBgn0039024	**2.14**	**2.82**		
CG17739	FBgn0033710	**2.14**			
CG4725	FBgn0039022	**2.20**			
Kaz-m1	FBgn0002578	**2.30**			
l(2)34Fc	FBgn0261534	**2.33**			
scaf	FBgn0033033	**2.33**			
CG6225	FBgn0038072	**2.38**			
CG16712	FBgn0031561	**2.48**	**7.20**	**2.68**	
Bace	FBgn0032049	**2.48**			
iotaTry	FBgn0015001	**2.50**			
stv	FBgn0086708	**2.53**		**4.30**	
CG17477	FBgn0038479	**2.58**			
CG30043	FBgn0050043	**2.66**			
CG6337	FBgn0033873	**2.71**			
CG32483	FBgn0052483	**2.77**			**7.18**
CG6048	FBgn0029827	**2.87**			
CG5246	FBgn0038484	**2.91**			
CG3604	FBgn0031562	**2.91**	**3.70**		
zetaTry	FBgn0011556	**2.99**			
Nep1	FBgn0029843	**3.03**			

CG4053	FBgn0038482	**3.10**			
CG10472	FBgn0035670	**3.16**			
CG11961	FBgn0034436	**3.25**			
CG17475	FBgn0038481	**3.25**			
CG13160	FBgn0033720	**3.41**			
CG17134	FBgn0032304	**3.43**			
CG10051	FBgn0034437	**3.81**			
CG33127	FBgn0053127	**3.84**			
CG33459	FBgn0053459	**3.86**	**10.43**		
CG30371	FBgn0050371	**4.08**	**0.160**		
CG31233	FBgn0051233	**4.14**			
kappaTry	FBgn0043471	**4.38**			
CG4653	FBgn0030776	**4.41**			
CG31343	FBgn0051343	**4.41**	**12.63**		
lambdaTry	FBgn0043470	**4.47**			
Sp212	FBgn0053329	**4.82**			
CG1304	FBgn0031141	**5.35**			
CG3513	FBgn0031559	**5.35**	**11.02**		
CG31198	FBgn0051198	**5.58**			
CG8774	FBgn0038136	**7.73**			
Spn47C	FBgn0033574	**7.73**			
CG14820	FBgn0035718	**8.00**			
CG31265	FBgn0051265	**9.06**			
CG15254	FBgn0028949	**14.7**			
**signaling**					
PKD	FBgn0038603	**2.04**		**4.00**	
moody	FBgn0025631	**2.06**	**2.28**		
Syn	FBgn0004575	**2.07**	**2.44**		
Fas2	FBgn0000635	**2.11**		**0.479**	
mthl6	FBgn0035789	**2.14**			
CrzR	FBgn0036278	**2.16**			
Ag5r	FBgn0015010	**2.19**	**6.17**		
Tk	FBgn0037976	**2.22**			
Galphaf	FBgn0010223	**2.30**	**6.31**		
Amph	FBgn0027356	**2.35**			
Dh31-R	FBgn0052843	**2.50**			
CG12290	FBgn0039419	**2.57**			
CG7054	FBgn0038972	**2.66**	**3.91**		
Pkcdelta	FBgn0259680	**2.71**			
CG15529	FBgn0039748	**2.83**			
Mctp	FBgn0034389	**2.95**	**0.300**	**2.11**	**2.25**
Pde6	FBgn0038237	**2.95**	**5.21**		
CG5402	FBgn0039521	**3.39**			
Ac76E	FBgn0004852	**3.43**			
CG8907	FBgn0038466	**3.48**	**2.81**		
CG9498	FBgn0031801	**4.56**	**3.62**		
RyR	FBgn0011286	**8.88**			
**tissue structure**					
CAP	FBgn0033504	**2.03**	**2.32**		
Ag5r2	FBgn0020508	**2.03**			
Cpr97Eb	FBgn0039481	**2.04**			
LamC	FBgn0010397	**2.08**			**2.95**

CG8927	FBgn0038405	**2.08**			
Idgf5	FBgn0064237	**2.11**	**2.30**		
Lcp65Af	FBgn0020639	**2.14**			
CG14304	FBgn0038629	**2.16**			
CG7298	FBgn0036948	**2.17**			
kst	FBgn0004167	**2.19**			
Acp1	FBgn0014454	**2.25**			
Cht7	FBgn0035398	**2.27**	**0.194**		
verm	FBgn0261341	**2.28**			
Cpr11B	FBgn0030398	**2.30**			
Cda5	FBgn0051973	**2.33**	**0.109**		
obst-I	FBgn0052304	**2.33**			
Cht6	FBgn0263132	**2.38**			
CG4367	FBgn0038783	**2.39**			
obst-H	FBgn0053983	**2.39**	**11.78**		
Cpr67Fa1	FBgn0036108	**2.41**			
Gasp	FBgn0026077	**2.43**			
Cpr67Fa2	FBgn0036109	**2.45**			
CG7252	FBgn0036226	**2.46**			
CG33468	FBgn0053468	**2.46**	**4.91**	**2.57**	
nrm	FBgn0262509	**2.48**			
Lcp65Ag3	FBgn0086611	**2.50**			
Lcp65Ag2	FBgn0020637	**2.51**			
Muc11A	FBgn0052656	**2.68**			
CG32284	FBgn0052284	**2.69**			
obst-F	FBgn0036947	**2.77**			
Mur18B	FBgn0030999	**2.79**			
CG43896	FBgn0264488	**2.85**			
CG43896	FBgn0264488	**2.91**			
Cpr62Bc	FBgn0035281	**2.95**			
CG5883	FBgn0036225	**2.95**			
PH4alphaSG1	FBgn0051014	**3.07**			
Muc96D	FBgn0051439	**3.18**			
PH4alphaPV	FBgn0051015	**3.27**			
Tg	FBgn0031975	**3.41**	**0.241**		
Muc18B	FBgn0031000	**3.63**	**2.48**		
Cpr49Ah	FBgn0033731	**3.86**			
Cpr11A	FBgn0030394	**3.97**			
obst-E	FBgn0031737	**3.97**			
CG10725	FBgn0036362	**4.20**			
TwdlO	FBgn0039438	**4.35**			
Mal-A3	FBgn0002571	**4.47**			
ng2	FBgn0010294	**5.17**			
Cpr49Af	FBgn0033729	**5.21**			
ng1	FBgn0002933	**5.94**			
CG10154	FBgn0036361	**6.68**			
Muc55B	FBgn0034294	**6.96**			
CG15515	FBgn0039719	**7.16**			
CG4835	FBgn0035607	**10.1**			
Cpr47Eb	FBgn0033598	**14.1**	**25.21**		
CG12491	FBgn0034900	**31.8**	**6.23**		
**transport**					

CG16989	FBgn0025621	**2.03**			
Oatp74D	FBgn0036732	**2.04**	**2.37**		
Prestin	FBgn0036770	**2.06**	**2.22**		
CG7458	FBgn0037144	**2.06**			**0.413**
CG11147	FBgn0031734	**2.10**			
CG1718	FBgn0031170	**2.13**	**2.30**		
CG9702	FBgn0039787	**2.13**			
CG11897	FBgn0039644	**2.14**	**5.52**	**2.61**	**2.59**
CG5326	FBgn0038983	**2.16**	**2.44**		
CG7084	FBgn0038938	**2.16**			
MRP	FBgn0032456	**2.16**	**2.08**		
CG6901	FBgn0038414	**2.17**			
CG8028	FBgn0031010	**2.17**			
CG9413	FBgn0030574	**2.20**			
Mdr65	FBgn0004513	**2.27**			
ppk6	FBgn0034489	**2.39**			
p24-2	FBgn0053105	**2.39**	**0.122**		
CG31792	FBgn0051792	**2.43**			
Vha100-4	FBgn0038613	**2.43**			
CG30016	FBgn0050016	**2.50**			
hoe1	FBgn0041150	**2.53**			
Tsf1	FBgn0022355	**2.57**	**7.68**		
CG31743	FBgn0032618	**2.60**			**4.18**
path	FBgn0036007	**2.60**			
AQP	FBgn0033807	**2.64**			
Smvt	FBgn0039873	**2.68**			
Tsf1	FBgn0022355	**2.69**	**7.68**		
CG10505	FBgn0034612	**2.71**			
Oatp58Dc	FBgn0034716	**2.73**			
CG9981	FBgn0030746	**2.77**		**9.00**	
Calx	FBgn0013995	**2.81**			
CG13124	FBgn0032156	**2.91**			
Rab9	FBgn0032782	**2.93**	**2.56**		
CG17636	FBgn0025837	**2.97**			
CG33281	FBgn0053281	**3.01**			
Rh50	FBgn0028699	**3.01**			
CG30272	FBgn0050272	**3.05**			
Oatp33Eb	FBgn0032435	**3.14**			
CG7912	FBgn0039736	**3.16**			
CG8791	FBgn0033234	**3.20**	**13.03**		
salt	FBgn0039872	**3.27**			
CG8051	FBgn0031012	**4.03**			
CG2187	FBgn0017448	**4.06**	**3.10**		
st	FBgn0003515	**4.06**			
CG4830	FBgn0037996	**4.35**			
CG6293	FBgn0037807	**4.99**	**3.59**		
CG9780	FBgn0037230	**5.13**	**17.37**		
CG18327	FBgn0033904	**5.66**			
Nha2	FBgn0263390	**6.11**		**2.32**	
CG4991	FBgn0030817	**6.82**	**3.76**		
CG9825	FBgn0034783	**6.96**			
CG8654	FBgn0034479	**9.85**			

**unknown function**					
CG9682	FBgn0039760	**2.00**			
CG11158	FBgn0030511	**2.01**	**2.73**		
Ilp8	FBgn0036690	**2.01**			
CG17147	FBgn0260393	**2.01**			
CG7992	FBgn0031004	**2.03**			
CG15597	FBgn0037420	**2.03**			
CG13360	FBgn0025620	**2.04**			
CG15236	FBgn0033108	**2.04**			
Piezo	FBgn0264953	**2.04**			
Rcd2	FBgn0037012	**2.04**	**6.47**		
CG32485	FBgn0052485	**2.04**			
CG11168	FBgn0039249	**2.06**			
jp	FBgn0032129	**2.06**	**0.303**		
CG15523	FBgn0039727	**2.06**	**0.328**		
CG5399	FBgn0038353	**2.06**	**28.01**	**2.91**	
CG5819	FBgn0034717	**2.07**			
CG15043	FBgn0030929	**2.07**			
CG8177	FBgn0036043	**2.08**	**0.177**		
CG8087	FBgn0038241	**2.08**	**0.445**		
CG31321	FBgn0051321	**2.10**			
CG12075	FBgn0030065	**2.10**			
CG7920	FBgn0039737	**2.10**			
CG14907	FBgn0038455	**2.10**	**6.93**		
Tyler	FBgn0031038	**2.10**	**2.09**		
CG12826	FBgn0033207	**2.10**	**6.33**		
CG5506	FBgn0036766	**2.10**			
LP09564	FBcl0187291	**2.10**			
Pebp1	FBgn0038973	**2.11**			
CG32714	FBgn0260483	**2.13**			
CG10936	FBgn0034253	**2.14**	**9.27**		
CG13806	FBgn0035325	**2.14**			
Rootletin	FBgn0039152	**2.16**			
CG5084	FBgn0034288	**2.16**			
GM03661	FBcl0137693	**2.16**			
CG14456	FBgn0037176	**2.17**			
atilla	FBgn0032422	**2.17**			
CG2010	FBgn0039667	**2.20**			
Pdxk	FBgn0085484	**2.22**			
CG5767	FBgn0034292	**2.22**			
CG8852	FBgn0031548	**2.23**			
CG31974	FBgn0051974	**2.23**	**4.74**		**2.12**
CG40198	FBgn0058198	**2.23**			
CG12177	FBgn0030510	**2.25**	**6.26**		
CG43693	FBgn0263776	**2.27**			
CG13912	FBgn0035186	**2.27**		**2.23**	
CG13003	FBgn0030798	**2.28**			
Ect4	FBgn0262579	**2.28**		**2.17**	
CG11313	FBgn0039798	**2.30**	**5.46**		
CG14273	FBgn0032024	**2.30**			
CG32603	FBgn0052603	**2.30**			
CG5866	FBgn0038508	**2.31**			

TwdlBeta	FBgn0033658	**2.31**			
CG31769	FBgn0051769	**2.33**	**2.49**		**0.380**
CG4733	FBgn0038744	**2.33**			
CG34120	FBgn0083956	**2.35**			
CG6023	FBgn0030912	**2.35**	**2.88**		
CG43078	FBgn0262508	**2.35**			
CG2157	FBgn0030244	**2.35**			
CG9399	FBgn0037715	**2.36**	**2.74**		
CG30411	FBgn0050411	**2.36**	**0.040**		
CG9568	FBgn0032087	**2.38**			
CG31446	FBgn0051446	**2.38**			
CG18635	FBgn0034279	**2.38**			
GIIIspla2	FBgn0030013	**2.39**			
CG1698	FBgn0033443	**2.39**			
CG42249	FBgn0259101	**2.41**			
CG15199	FBgn0030270	**2.41**	**3.28**		
CG31086	FBgn0051086	**2.41**			
CG9626	FBgn0037565	**2.46**			
CG31259	FBgn0051259	**2.48**			
CG17108	FBgn0032285	**2.48**			
CG14893	FBgn0038451	**2.50**			
CG5535	FBgn0036764	**2.51**	**7.41**		
CG31269	FBgn0051269	**2.57**			
CG33469	FBgn0053469	**2.58**			
CG13323	FBgn0033788	**2.58**			
CG16820	FBgn0032495	**2.60**			
CR14033	FBgn0046776	**2.60**	**41.33**		
CG31464	FBgn0051464	**2.64**			
HDC07637		**2.64**			
ppk13	FBgn0053508	**2.66**			
SP1173	FBgn0035710	**2.71**			
ps	FBgn0261552	**2.71**			
CG7900	FBgn0037548	**2.71**	**2.96**		**0.476**
Lmpt	FBgn0261565	**2.73**			
CG31288	FBgn0051288	**2.75**	**28.68**		
CG17167	FBgn0039941	**2.75**			
CG12105	FBgn0035241	**2.75**			
Skeletor	FBgn0262717	**2.75**			
CG44250	FBgn0265185	**2.75**			
CG13492	FBgn0034662	**2.77**			
l(1)G0196	FBgn0027279	**2.79**			
HDC03517		**2.79**			
CG43897	FBgn0264489	**2.79**			
CT22789		**2.81**			
mtg	FBgn0260386	**2.83**			**2.11**
CR31781	FBgn0051781	**2.85**	**0.354**		
CG15412	FBgn0031528	**2.85**			
CG11160	FBgn0030257	**2.87**			
CG10597	FBgn0030832	**2.87**			
CG15741	FBgn0030338	**2.91**	**76.88**		
CG10732	FBgn0036365	**2.93**			
CG7631	FBgn0028945	**2.99**			

CG32198	FBgn0052198	**3.12**			
CG15213	FBgn0040843	**3.12**			
CG7300	FBgn0032286	**3.16**			
CG43427	FBgn0263346	**3.20**			
CG14852	FBgn0038242	**3.20**	**0.084**		
CG34278	FBgn0085307	**3.25**	**4.76**		
CG13102	FBgn0032088	**3.32**			
Cpr47Ec	FBgn0033600	**3.32**			
Obp83g	FBgn0046875	**3.32**			
CR31781	FBgn0051781	**3.34**	**0.354**		
GM10545	FBcl0137569	**3.34**			
HDC03535		**3.36**			
CG15530	FBgn0039752	**3.41**			
CG9396	FBgn0037714	**3.53**	**5.56**		
CT37020		**3.56**			
GstD7	FBgn0010043	**3.61**	**3.98**		**12.65**
CG13705	FBgn0035582	**3.78**			
CG13946	FBgn0040725	**3.92**	**30.28**		
CG4962	FBgn0036597	**4.03**			
CG14292	FBgn0038658	**4.11**			
CG42565	FBgn0260767	**4.47**			
CG32241	FBgn0052241	**4.72**			
CG5810	FBgn0038866	**4.79**			
CG13460	FBgn0036471	**4.79**			
CG18649	FBgn0036469	**5.06**			
CG11350	FBgn0035552	**5.28**			
CG8773	FBgn0038135	**5.35**			
CG43078	FBgn0262508	**5.58**			
CG2064	FBgn0033205	**5.86**	**2.24**		
CG14120	FBgn0036321	**6.02**			
CG14879	FBgn0038419	**6.15**			
CG32073	FBgn0052073	**6.19**	**19.44**		
CG10953	FBgn0034204	**6.23**			
CG9555	FBgn0032085	**8.00**			
Cyp4p3	FBgn0033397	**8.75**	**9.73**	**2.75**	
CG12506	FBgn0031276	**9.92**	**18.91**		
CG32564	FBgn0052564	**14.93**	**3.08**		
CG8534	FBgn0037761	**3.10**			
CG13640	FBgn0039237	**6.23**			
Chd1* effect: *DOWN					
**detoxification**					
Cyp312a1	FBgn0036778	**0.497**			
Cyp6d5	FBgn0038194	**0.490**		**3.00**	**13.46**
Cyp309a1	FBgn0031432	**0.444**	**2.62**		
GstE3	FBgn0063497	**0.432**		**5.89**	
GstD10	FBgn0042206	**0.409**			
Hsp22	FBgn0001223	**0.342**	**2.94**		
GstT3	FBgn0031117	**0.337**	**2.41**		
CG6762	FBgn0030876	**0.316**			
Hsp26	FBgn0001225	**0.304**			
GstE1	FBgn0034335	**0.304**	**2.21**		
MtnD	FBgn0053192	**0.287**	**12.01**		

CG17323	FBgn0032713	**0.272**			
MtnC	FBgn0038790	**0.248**			
GstD2	FBgn0010038	**0.235**	**9.61**	**2.74**	
GstD4	FBgn0010040	**0.216**			
Hsp67Bc	FBgn0001229	**0.200**	**2.74**	**2.38**	
NLaz	FBgn0053126	**0.171**			
CG7227	FBgn0031970	**0.143**	**0.310**		
MtnB	FBgn0002869	**0.092**			
**development**					
yellow-d2	FBgn0034856	**0.497**			
sad	FBgn0003312	**0.493**			
rdo	FBgn0243486	**0.473**	**0.207**		
ImpE1	FBgn0001253	**0.435**			
in	FBgn0001259	**0.432**			
neo	FBgn0039704	**0.409**			
CG15153	FBgn0032663	**0.361**			
CG18607	FBgn0034429	**0.344**			
nvd	FBgn0259697	**0.312**			
CG9005	FBgn0033638	**0.272**	**0.260**		
ImpE1	FBgn0001253	**0.264**			
CG2016	FBgn0250839	**0.188**			
Eig71Ed	FBgn0004591	**0.184**	**0.127**		
Yp3	FBgn0004047	**0.180**		**16.14**	
Eip74EF	FBgn0000567	**0.176**	**0.341**		
ImpE3	FBgn0001255	**0.171**			**2.10**
Eip78C	FBgn0004865	**0.144**	**0.136**	**2.12**	
ImpE2	FBgn0001254	**0.132**			
Eig71Ea	FBgn0004588	**0.126**	**0.037**		
Eig71Ec	FBgn0004590	**0.115**			
slbo	FBgn0005638	**0.115**	**5.57**		
Eig71Eg	FBgn0004594	**0.103**	**0.027**		
Eig71Eb	FBgn0004589	**0.023**	**0.403**		
**immune response**					
sn	FBgn0003447	**0.476**			
Hsp27	FBgn0001226	**0.457**			**2.41**
Drs	FBgn0010381	**0.451**	**4.28**	**2.53**	**3.78**
Mtk	FBgn0014865	**0.438**		**4.93**	**6.09**
CG8157	FBgn0034010	**0.374**	**3.95**		
CG4091	FBgn0034894	**0.354**			
LysX	FBgn0004431	**0.344**	**33.21**	**2.92**	
AttA	FBgn0012042	**0.337**		**7.68**	
E(spl)malpha-BFM	FBgn0002732	**0.334**	**3.57**		
spz3	FBgn0031959	**0.332**			
Tsp42Ep	FBgn0033137	**0.332**	**0.395**		
Atg7	FBgn0034366	**0.306**			
dsb	FBgn0035290	**0.301**	**0.423**		
E(spl)m2-BFM	FBgn0002592	**0.291**			
CG9616	FBgn0038214	**0.289**			
NimC2	FBgn0028939	**0.287**	**0.140**		
spirit	FBgn0030051	**0.266**			
Mmp1	FBgn0035049	**0.264**	**15.55**	**2.88**	**3.34**
wbl	FBgn0004003	**0.261**	**0.495**		

Npc2e	FBgn0051410	**0.233**	**0.028**		
PGRP-SC2	FBgn0043575	**0.222**	**2.66**	**2.37**	
santa-maria	FBgn0025697	**0.218**	**0.036**		
IM2	FBgn0025583	**0.218**			
CG11425	FBgn0037167	**0.183**			
CG2065	FBgn0033204	**0.163**	**9.00**	**2.72**	
CG3397	FBgn0037975	**0.162**		**3.78**	
Spn43Aa	FBgn0024294	**0.160**	**0.336**		
CG6429	FBgn0046999	**0.147**			
IM3	FBgn0040736	**0.139**	**11.65**		
PGRP-SB1	FBgn0043578	**0.108**		**3.12**	
IM1	FBgn0034329	**0.104**	**33.02**		
Drsl5	FBgn0035434	**0.092**			
Spn100A	FBgn0039795	**0.067**			
CG13606	FBgn0039161	**0.042**			
PGRP-SB2	FBgn0043577	**0.023**	**12.29**		
Drsl2	FBgn0052279	**0.020**			
**intracellular processes**					
CG5745	FBgn0038855	**0.497**			
CG17754	FBgn0030114	**0.490**	**2.66**		**2.07**
insc	FBgn0011674	**0.490**			
CG8420	FBgn0037664	**0.490**			
GV1	FBgn0027790	**0.486**			
RpL22-like	FBgn0034837	**0.483**			
RpS19b	FBgn0039129	**0.483**			
His2B:CG17949	FBgn0061209	**0.476**			
CG4496	FBgn0031894	**0.476**	**0.293**		
lbm	FBgn0016032	**0.476**			
nsr	FBgn0034740	**0.476**			
Eip71CD	FBgn0000565	**0.473**			
Drep-3	FBgn0028407	**0.470**			
CG32971	FBgn0052971	**0.470**			
tbrd-1	FBgn0039124	**0.470**			
Scr	FBgn0003339	**0.467**			
sc	FBgn0004170	**0.460**			
MED9	FBgn0260401	**0.457**			
CG30431	FBgn0050431	**0.457**			
en	FBgn0000577	**0.454**			
CG17450	FBgn0040028	**0.454**			
CG3726	FBgn0029824	**0.448**			
thoc6	FBgn0036263	**0.448**			
RpL37b	FBgn0034822	**0.444**			
Lmpt	FBgn0261565	**0.444**			
CG31642	FBgn0051642	**0.435**		**79.80**	
Hsp67Ba	FBgn0001227	**0.429**	**3.25**		
Dark	FBgn0263864	**0.418**			**2.02**
CG31274	FBgn0051274	**0.418**	**0.342**		**5.24**
betaNACtes4	FBgn0030566	**0.415**			
CG6891	FBgn0030955	**0.412**			
CG12493	FBgn0035571	**0.406**	**117.75**		
GV1	FBgn0027790	**0.398**			
CG18446	FBgn0033458	**0.395**	**2.05**	**2.27**	

CG8335	FBgn0033069	**0.392**			
His1:CG31617	FBgn0051617	**0.387**			
Eip93F	FBgn0264490	**0.384**			
CR11386	FBgn0260447	**0.382**			
toy	FBgn0019650	**0.379**	**24.16**		
E(spl)m7-HLH	FBgn0002633	**0.376**			
CG4080	FBgn0035983	**0.369**	**0.405**		
Taf12L	FBgn0031623	**0.369**	**191.54**		
betaNACtes3	FBgn0052601	**0.369**			
CG12477	FBgn0036809	**0.356**			
CG8679	FBgn0032934	**0.344**			
E(spl)m8-HLH	FBgn0000591	**0.337**			
toy	FBgn0019650	**0.332**	**24.16**		
His2A:CG31618	FBgn0051618	**0.330**			
blanks	FBgn0035608	**0.321**	**98.58**		**0.453**
CG4021	FBgn0034659	**0.281**			
CG7804	FBgn0036496	**0.270**			
CG9989	FBgn0039593	**0.261**		**8.99**	
CG34434	FBgn0250904	**0.200**			
CG17386	FBgn0033936	**0.087**			
Chd1	FBgn0250786	**0.013**			
**metabolism**					
CAH1	FBgn0027844	**0.493**	**2.25**	**2.30**	**2.11**
Ho	FBgn0037933	**0.493**			
CG1637	FBgn0030245	**0.486**			
CG1941	FBgn0033214	**0.486**			
CG7742	FBgn0031690	**0.486**			
CG3376	FBgn0034997	**0.486**	**0.330**		
CLS	FBgn0039360	**0.476**			
wat	FBgn0039620	**0.470**			
CG5554	FBgn0034914	**0.463**	**0.379**		
Sep5	FBgn0026361	**0.454**	**2.36**		
Scp2	FBgn0020907	**0.451**			
Dyrk2	FBgn0016930	**0.448**		**2.13**	
ade3	FBgn0000053	**0.444**	**0.423**		
CG5854	FBgn0039130	**0.441**	**0.477**		
CG1942	FBgn0033215	**0.426**			
Oscillin	FBgn0031717	**0.426**	**0.302**		
CG15343	FBgn0030029	**0.423**	**0.124**		**3.07**
CG18528	FBgn0039189	**0.423**			
CG4546	FBgn0038373	**0.418**			
CG9509	FBgn0030594	**0.415**	**0.147**		
CG8565	FBgn0030697	**0.406**			
CG13833	FBgn0039040	**0.398**			
CG8112	FBgn0037612	**0.395**	**0.205**		
CG3264	FBgn0034712	**0.392**			
Dhpr	FBgn0035964	**0.387**			
CG6287	FBgn0032350	**0.382**		**2.03**	
CG11170	FBgn0034705	**0.379**	**0.365**		
slow	FBgn0035539	**0.376**	**2.34**		
CG4586	FBgn0029924	**0.376**	**0.432**		
CG17191	FBgn0039473	**0.376**			

CG12539	FBgn0030586	**0.374**			
MESR6	FBgn0036846	**0.361**	**0.364**		
CG33096	FBgn0053096	**0.361**			
yellow-f	FBgn0041710	**0.358**	**0.330**	**2.09**	
Pu	FBgn0003162	**0.356**			
CG3940	FBgn0037788	**0.356**		**8.33**	
CG7320	FBgn0036782	**0.349**			
Est-P	FBgn0000594	**0.342**			
CG8100	FBgn0036410	**0.339**			
Ady43A	FBgn0026602	**0.339**			
Nmdmc	FBgn0010222	**0.330**			
CG14946	FBgn0032405	**0.310**			
Asph	FBgn0034075	**0.308**	**0.427**		**2.25**
Oat	FBgn0022774	**0.306**	**0.077**		**3.00**
AANATL2	FBgn0031791	**0.295**	**0.305**		
CG4842	FBgn0036620	**0.289**			
spok	FBgn0086917	**0.289**			
Ddc	FBgn0000422	**0.259**			
CG5618	FBgn0036975	**0.252**			
Acp63F	FBgn0015585	**0.237**			
CG10178	FBgn0032684	**0.178**			
CG5171	FBgn0031907	**0.149**		**20.62**	
yellow-c	FBgn0041713	**0.146**			**4.92**
CG4382	FBgn0032132	**0.131**			
CG8093	FBgn0033999	**0.113**			
CG6277	FBgn0039475	**0.107**			
w	FBgn0003996	**0.079**	**4.39**		
CG9452	FBgn0036877	**0.073**			
CG18606	FBgn0034428	**0.069**			
CG14406	FBgn0030595	**0.061**	**0.314**		
CG2070	FBgn0033203	**0.047**			
St2	FBgn0037665	**0.045**			
e	FBgn0000527	**0.042**	**2.93**		**5.46**
**proteolysis**					
hh	FBgn0004644	**0.497**			
CG31778	FBgn0051778	**0.483**			
S-Lap3	FBgn0045770	**0.457**			
Rpt6R	FBgn0039788	**0.451**	**36.11**		
Spn88Eb	FBgn0038299	**0.451**	**6.72**		
S-Lap2	FBgn0052351	**0.451**			
CG8586	FBgn0033320	**0.438**		**6.89**	
Sb	FBgn0003319	**0.432**			
Spn42Dd	FBgn0028988	**0.426**	**0.341**		
CG11023	FBgn0031208	**0.420**			
Sp7	FBgn0037515	**0.401**			**0.466**
CG10764	FBgn0034221	**0.390**			**2.61**
Jon99Ci	FBgn0003358	**0.376**	**2.55**		
CG4408	FBgn0039073	**0.374**	**0.260**		
CG8550	FBgn0033742	**0.361**			
CG30289	FBgn0050289	**0.354**	**0.151**		
CG9850	FBgn0034903	**0.334**			
CG31777	FBgn0051777	**0.323**	**2.75**		

CG4914	FBgn0036436	**0.323**			
CG30288	FBgn0050288	**0.312**	**0.407**		
CG9850	FBgn0034903	**0.304**			
Nep2	FBgn0027570	**0.283**		**4.58**	
NnaD	FBgn0265726	**0.266**			
CG11529	FBgn0036264	**0.255**			
CG3355	FBgn0031619	**0.252**			
CG30098	FBgn0050098	**0.250**			
SP1029	FBgn0263236	**0.200**			
CG5470	FBgn0038384	**0.199**			
CG30091	FBgn0050091	**0.187**			**11.62**
CG4650	FBgn0032549	**0.173**	**3.36**		
CG1773	FBgn0033439	**0.168**	**0.148**		
CG3700	FBgn0034796	**0.157**			
CG10073	FBgn0034440	**0.154**			
CG3502	FBgn0046253	**0.153**			
CG3097	FBgn0029804	**0.149**			
CG7906	FBgn0036417	**0.139**			
CG13748	FBgn0033355	**0.133**			
CG7924	FBgn0036416	**0.076**			
CG32762	FBgn0052762	**0.067**			
CG11459	FBgn0037396	**0.062**			
CG12951	FBgn0037677	**0.062**			
**signaling**					
bib	FBgn0000180	**0.493**			
SoxN	FBgn0029123	**0.483**			
mthl5	FBgn0037960	**0.476**			**2.17**
E(spl)m4-BFM	FBgn0002629	**0.470**			
CG17760	FBgn0033756	**0.412**	**3.33**		
Takl1	FBgn0046689	**0.366**			
CG6908	FBgn0037936	**0.328**			
Wnt2	FBgn0004360	**0.304**			
Buffy	FBgn0040491	**0.297**	**0.258**		
CG9259	FBgn0032913	**0.252**			
CG32447	FBgn0052447	**0.228**			
CG31104	FBgn0051104	**0.133**			
Obp83ef	FBgn0046876	**0.063**	**0.106**		
CG11893	FBgn0039316	**0.045**	**40.01**		
**tissue structure**					
Cpr51A	FBgn0033942	**0.486**	**0.121**		
Cpr12A	FBgn0030494	**0.486**			
Sema-2b	FBgn0264273	**0.476**			
Pcp	FBgn0003046	**0.463**	**0.304**		
CG12009	FBgn0035430	**0.438**			
a	FBgn0000008	**0.420**			
CG11905	FBgn0036678	**0.374**			
Ama	FBgn0000071	**0.371**	**2.27**	**2.31**	**3.01**
TwdlE	FBgn0031957	**0.325**			
CG14957	FBgn0035412	**0.270**			
b	FBgn0000153	**0.250**			
Lcp65Ae	FBgn0020640	**0.245**			
mey	FBgn0039851	**0.235**			

Lcp65Ad	FBgn0020641	**0.230**			
Cpr65Ec	FBgn0035737	**0.230**	**2.41**		
Acp65Aa	FBgn0020765	**0.224**			
CG5756	FBgn0034301	**0.177**			
Cpr66Cb	FBgn0035875	**0.138**			
CG14687	FBgn0037835	**0.136**			
Muc68E	FBgn0053265	**0.080**			
**transport**					
Porin2	FBgn0069354	**0.493**			
CG7442	FBgn0037140	**0.490**	**0.403**		
CG5805	FBgn0039223	**0.486**			
CG31787	FBgn0051787	**0.483**			
Syx13	FBgn0036341	**0.480**			
Atet	FBgn0020762	**0.470**			**2.06**
CG8925	FBgn0038404	**0.438**			
Orct	FBgn0019952	**0.426**	**0.287**		
Tsp42Ek	FBgn0033133	**0.418**			
CG14855	FBgn0038260	**0.418**	**4.89**		
CG7777	FBgn0033635	**0.415**	**0.096**		
yin	FBgn0265575	**0.398**		**2.05**	
Glut1	FBgn0264574	**0.395**		**2.11**	
E23	FBgn0020445	**0.384**	**0.156**		
CG17036	FBgn0032449	**0.382**			
CG13426	FBgn0034510	**0.356**			
ZnT35C	FBgn0028516	**0.264**			
twz	FBgn0034636	**0.232**			**4.25**
CG3823	FBgn0029863	**0.212**			
CG31636	FBgn0051636	**0.210**			
CG2663	FBgn0037323	**0.209**			
Oatp26F	FBgn0051634	**0.193**			
CG1732	FBgn0039915	**0.189**	**2.49**		
bw	FBgn0000241	**0.163**			
sut2	FBgn0028562	**0.159**	**2.00**		
CG3649	FBgn0034785	**0.147**			
CG42269	FBgn0259164	**0.111**			
**unknown function**					
CG5780	FBgn0032446	**0.500**			
CG15525	FBgn0039732	**0.500**			
CG2837	FBgn0031646	**0.497**			
CG6685	FBgn0036062	**0.497**			
CG18249	FBgn0037553	**0.497**			
CG12674	FBgn0031388	**0.493**			
CG1124	FBgn0037290	**0.493**			**5.25**
CR6900	FBgn0030958	**0.493**	**0.489**		
CG11882	FBgn0039642	**0.490**			
CG5968	FBgn0032588	**0.490**			
CG13962	FBgn0032824	**0.490**	**2.09**		
CG15210	FBgn0040850	**0.490**	**0.350**		
CG9686	FBgn0030158	**0.486**			
HDC06936		**0.483**	**0.432**		
CG31600	FBgn0051600	**0.483**			
CG12880	FBgn0046258	**0.483**			

Atg18b	FBgn0032935	**0.480**			
l(1)G0469	FBgn0040153	**0.480**			
CG6675	FBgn0032973	**0.480**			
CG8031	FBgn0038110	**0.480**	**0.390**		
CG3831	FBgn0034804	**0.480**			**2.22**
IM18	FBgn0067903	**0.480**			
CG10933	FBgn0034264	**0.476**			
CG13905	FBgn0035176	**0.473**			
MESK4	FBgn0043069	**0.473**	**0.342**		**6.97**
Mur29B	FBgn0051901	**0.470**			
CT36057		**0.470**			
CG13618	FBgn0039203	**0.467**			
HDC06631		**0.467**	**2.80**		
CT34146		**0.463**			
CG32023	FBgn0052023	**0.460**			
LD44795	FBcl0167607	**0.457**			
TwdlT	FBgn0029170	**0.457**			
orb2	FBgn0264307	**0.457**			
CG42269	FBgn0259164	**0.457**			
CG1999	FBgn0029947	**0.457**			
CG31323	FBgn0051323	**0.457**	**0.254**		
CG13116	FBgn0032139	**0.454**		**3.50**	
CG31324	FBgn0051324	**0.454**			
CG5194	FBgn0035955	**0.454**			
CG10912	FBgn0034296	**0.448**			
CG33096	FBgn0053096	**0.444**			
CG11275	FBgn0034706	**0.444**	**0.279**		
CG18343	FBgn0033683	**0.444**			
spz6	FBgn0035056	**0.444**			
CG31538	FBgn0051538	**0.444**			
CG11474	FBgn0034688	**0.444**	**0.238**		
CG14567	FBgn0037126	**0.441**	**0.369**		
CG9837	FBgn0037635	**0.441**			**0.320**
CG34424	FBgn0085453	**0.441**			
CG13255	FBgn0040636	**0.438**	**0.270**		
CG3223	FBgn0037538	**0.438**			
CG32259	FBgn0052259	**0.438**			
CG31861	FBgn0051861	**0.432**			
CG13186	FBgn0033680	**0.432**			
CG14109	FBgn0036364	**0.432**	**8.40**		
CIAPIN1	FBgn0001977	**0.432**			
CG1273	FBgn0035522	**0.429**			
CG33252	FBgn0053252	**0.429**			
Gbp	FBgn0034199	**0.429**		**8.40**	**2.72**
CG18190	FBgn0034403	**0.429**	**8.35**		
CG13488	FBgn0034670	**0.426**			
CG8160	FBgn0034011	**0.423**	**12.11**		
CG9801	FBgn0037623	**0.423**	**0.360**		**4.13**
CG4438	FBgn0032115	**0.423**			
CG14275	FBgn0032022	**0.415**	**35.52**		
CG12655	FBgn0031080	**0.406**	**7.38**		
CG15544	FBgn0039804	**0.406**			**2.40**

LP03188	FBcl0187891	**0.406**			
CG15905	FBgn0034462	**0.403**			
NnaD	FBgn0265726	**0.401**			
HDC12400		**0.398**			
CG40294	FBgn0058294	**0.398**			
CG7841	FBgn0036502	**0.398**			
CG11737	FBgn0037592	**0.395**			
CG31525	FBgn0051525	**0.392**			
CG9040	FBgn0036394	**0.392**	**4.54**		
CR_TC_GH03576		**0.390**			
CG12470	FBgn0040371	**0.387**			
Dtg	FBgn0038071	**0.387**			
CG2444	FBgn0030326	**0.384**			
nyo	FBgn0039852	**0.374**			
CG15818	FBgn0031910	**0.374**			
CG30417	FBgn0050417	**0.371**			
HDC14735		**0.371**			
Ste	FBgn0003523	**0.366**			
CG15068	FBgn0040733	**0.363**	**14.12**		
RE54004	FBcl0204065	**0.361**			
DM.2L.4959		**0.361**			
CG7778	FBgn0032025	**0.356**			
CG2201	FBgn0032955	**0.349**			
CG13856	FBgn0038959	**0.342**			
CG32115	FBgn0052115	**0.332**	**0.362**		
CG12481	FBgn0030542	**0.325**			
CG13024	FBgn0036665	**0.325**			
CG13082	FBgn0032803	**0.321**			
CG1172	FBgn0264712	**0.316**			
CG2082	FBgn0027608	**0.314**	**0.284**		
CG15208	FBgn0030247	**0.308**			
CG33143	FBgn0053143	**0.304**			
CG15756	FBgn0030493	**0.301**			
CG10516	FBgn0036549	**0.293**			**2.10**
CG18622	FBgn0038460	**0.291**			
CG2082	FBgn0027608	**0.289**	**0.284**		
CG2277	FBgn0035204	**0.272**			
CG5391	FBgn0038943	**0.268**			
CG8483	FBgn0038126	**0.268**			
CG13722	FBgn0035553	**0.238**			
CG13314	FBgn0035949	**0.207**			
CG16836	FBgn0040735	**0.207**	**20.27**		
CG16886	FBgn0028938	**0.204**			
CG34296	FBgn0085325	**0.132**			
CG4151	FBgn0029770	**0.117**	**0.366**		
CR43242	FBgn0262887	**0.097**		**0.252**	
pncr015:3L	FBgn0063083	**0.086**			
CG5697	FBgn0038846	**0.045**			
CG9822	FBgn0034623	**0.036**			
CG42717	FBgn0261634	**0.032**			

Transcripts that are up- or down-regulated by homozygous null mutation of *Chd1* in L3 larvae or RNAi-mediated depletion of H1 in L3 salivary glands, HP1 in Kc cells and ISWI in SL2 cells. Fold change is calculated from Affymetrix microarray data relative to wild-type control.
