# Supplementary material for: A Genetic Screen and Transcript Profiling Reveal a Shared Regulatory Program for Drosophila Linker Histone H1 and Chromatin Remodeler CHD1
Source: G3 (Bethesda). 2015 Jan 27;5(4):677–87. doi: 10.1534/g3.115.016709 (PMC4390582; doi:10.1534/g3.115.016709)
Supplement: Supporting Information [file supp_g3.115.016709_TableS3.pdf]

Table S3 Overlap of transcripts regulated by HP1, H1 and CHD1.

SYMBOL/ TRANSCRIPT	FLYBASE ID	FOLD CHANGE		
		**HP1 KD**	**H1 KD**	***Chd1***
**HP1 depletion effect:** ***UP***				
CR31451	FBgn0051451	**181.09**	**8.26**	
CG31140	FBgn0051140	**79.80**	**6.31**	
G6	FBte0000958	**67.66**		
CG31642	FBgn0051642	**51.14**		**0.435**
AT07338	FBcl0481740	**50.00**		
TART		**35.15**		
CG3635	FBgn0032981	**34.71**	**6.77**	
CG18605	FBgn0034411	**24.98**		
Def	FBgn0010385	**22.33**		
CG10126	FBgn0038088	**21.46**		
springer	FBte0000333	**20.62**		
CG17124	FBgn0032297	**20.00**	**2.46**	
gypsy6	FBte0001175	**19.16**		
CG11714	FBgn0036170	**19.04**	**3.88**	
CG40295	FBgn0058295	**18.09**	**11.97**	
HDC20537		**16.14**	**2.13**	
CG5773	FBgn0034290	**15.22**		
CG13795	FBgn0031937	**13.84**		
CG5171	FBgn0031907	**12.85**		**0.149**
accord	FBte0000956	**11.76**		
CG5399	FBgn0038353	**11.64**	**28.01**	**2.06**
CG32091	FBgn0052091	**10.80**		
edin	FBgn0052185	**10.48**		
CG32032	FBgn0043806	**10.10**		
gypsy2	FBte0001040	**9.84**		
tok	FBgn0004885	**9.72**	**2.11**	
CG9691	FBgn0030160	**9.48**		
Yp3	FBgn0004047	**9.00**		**0.180**
invader3	FBte0000619	**8.99**		
CG13315	FBgn0040827	**8.90**		
CG3961	FBgn0036821	**8.86**		
CAH1	FBgn0027844	**8.65**	**2.25**	**0.493**
AttC	FBgn0041579	**8.50**		
gypsy	FBte0000021	**8.45**		
Cyp6a13	FBgn0033304	**8.40**	**5.64**	
Drs	FBgn0010381	**8.33**	**4.28**	**0.451**
Idgf1	FBgn0020416	**8.29**		
Cyp12e1	FBgn0037817	**8.15**		
HMS-Beagle	FBte0000726	**7.86**		
CG33775	FBgn0053775	**7.68**		
KCNQ	FBgn0033494	**7.66**		
Gpb5	FBgn0063368	**7.47**		
Gr94a	FBgn0041225	**7.33**	**5.19**	
CG33468	FBgn0053468	**7.30**	**4.91**	**2.46**
CG4752	FBgn0034733	**7.25**		
CG9981	FBgn0030746	**7.06**		**2.77**
aret	FBgn0000114	**6.89**		

CG45057	FBgn0266417	**6.74**	**2.20**	
Cyp6d4	FBgn0039006	**6.66**		
CG40115	FBgn0058115	**6.47**	**10.26**	
HDC20531		**6.34**		
Sid	FBgn0039593	**6.31**		**0.261**
CT39784		**6.11**	**14.84**	
HDC20523		**6.01**	**2.81**	
CG11672	FBgn0037563	**5.98**		
Tsf3	FBgn0034094	**5.89**		
Rh4	FBgn0003250	**5.82**		
CG10877	FBgn0038804	**5.73**	**0.157**	
CG9717	FBgn0039789	**5.71**		
CG18473	FBgn0037683	**5.69**	**3.14**	
bru-3	FBgn0264001	**5.67**		
rau	FBgn0031745	**5.63**		
CG15661	FBgn0034605	**5.55**	**12.72**	
Gbp	FBgn0034199	**5.52**		**0.429**
CG3940	FBgn0037788	**5.43**		**0.356**
CG10799	FBgn0033821	**5.41**	**3.26**	
PGRP-SD	FBgn0035806	**5.40**		
PGRP-SC2	FBgn0043575	**5.39**	**2.66**	**0.222**
Cyp12e1	FBgn0037817	**5.34**		
CG14164	FBgn0036057	**5.31**		
AttA	FBgn0012042	**5.30**		**0.337**
CG4928	FBgn0027556	**5.30**	**0.315**	
CG14695	FBgn0037850	**5.29**		
Zasp52	FBgn0265991	**5.26**	**0.492**	
CG5367	FBgn0032228	**5.24**		
CG18446	FBgn0033458	**5.20**	**2.05**	**0.395**
Mct1	FBgn0023549	**5.19**	**0.395**	
invader4	FBte0000292	**5.17**		
Pka-C3	FBgn0000489	**5.16**		
CG17124	FBgn0032297	**5.14**	**4.46**	
CG9919	FBgn0030742	**5.13**		
SPH93	FBgn0032638	**5.12**	**11.67**	
Corp	FBgn0030028	**5.08**	**4.57**	
Apoltp	FBgn0032136	**5.06**	**2.89**	**5.70**
spir	FBgn0003475	**5.06**		
CG8586	FBgn0033320	**5.03**		**0.438**
CG11052	FBgn0040524	**5.03**		
yin	FBgn0265575	**5.00**	**0.449**	**0.398**
CG43110	FBgn0262570	**4.99**	**0.432**	
Cyp9c1	FBgn0015040	**4.93**		**3.41**
CG5381	FBgn0032218	**4.85**		
CG32687	FBgn0052687	**4.82**	**2.09**	
Cad96Ca	FBgn0022800	**4.81**		
Tsp2A	FBgn0024361	**4.77**		
Cyp28d1	FBgn0031689	**4.76**		
Mctp	FBgn0034389	**4.72**	**0.300**	**2.95**
Ama	FBgn0000071	**4.72**	**2.27**	**0.371**
Jheh3	FBgn0034406	**4.69**		**2.68**
alphaTub67C	FBgn0087040	**4.63**		

CG5731	FBgn0032192	**4.60**	**2.72**	
GstE3	FBgn0063497	**4.59**		**0.432**
CG40124	FBgn0058124	**4.58**	**11.10**	
CG5853	FBgn0032167	**4.57**		
CT39116		**4.56**	**103.1**	
CG14606	FBgn0037485	**4.54**		
shf	FBgn0003390	**4.52**	**2.97**	
CG1294	FBgn0033030	**4.49**	**4.55**	
ItgalphaPS4	FBgn0034005	**4.49**	**3.91**	
PGRP-SA	FBgn0030310	**4.46**	**2.20**	
Cyp4p3	FBgn0033397	**4.43**	**9.73**	**8.75**
CG6709	FBgn0036056	**4.43**	**2.66**	
HDC20116		**4.41**		
Cyp12a5	FBgn0038680	**4.30**		
CG12698	FBgn0030721	**4.26**		
CG9170	FBgn0030716	**4.26**	**3.83**	
qbert		**4.26**		
RpS5b	FBgn0038277	**4.25**		
AttB	FBgn0041581	**4.22**		
Hf	FBgn0014000	**4.22**		
l(3)neo38	FBgn0265276	**4.17**		
HDC20280		**4.17**		
CG6330	FBgn0039464	**4.15**		
beat-IIIc	FBgn0032629	**4.13**	**2.39**	
alphaTub85E	FBgn0003886	**4.12**		
Swim	FBgn0034709	**4.11**	**9.06**	
Mal-A5	FBgn0050359	**4.11**		
GstE11	FBgn0034354	**4.06**	**0.341**	
CG2930	FBgn0028491	**4.06**		
Eip78C	FBgn0004865	**4.04**	**0.136**	**0.144**
Epac	FBgn0085421	**4.01**	**9.43**	
CG3246	FBgn0031538	**4.00**		
TpnC4	FBgn0033027	**4.00**	**3.70**	
TART		**3.97**		
Myo28B1	FBgn0040299	**3.95**	**2.21**	
CG14451	FBgn0037183	**3.94**		
kek1	FBgn0015399	**3.90**	**5.43**	
Esyt2	FBgn0266758	**3.88**		
CG13117	FBgn0032140	**3.85**	**2.71**	
CG40274	FBgn0058274	**3.85**	**5.22**	
micropia	FBte0000042	**3.83**		
CG5322	FBgn0032253	**3.80**		
CG11400	FBgn0034198	**3.78**	**19.24**	
CG16947	FBgn0031816	**3.78**	**0.285**	
GATE	FBte0000359	**3.78**		
NT5E-2	FBgn0050104	**3.76**	**0.196**	
CG9150	FBgn0031775	**3.72**		
spir	FBgn0003475	**3.67**		
Ir41a	FBgn0040849	**3.67**		
Tsp42Ed	FBgn0029507	**3.66**		
l(3)neo38	FBgn0265276	**3.65**		
l(3)neo38	FBgn0265276	**3.65**		

CG12868	FBgn0033945	**3.64**	**2.74**	
CG17032	FBgn0036547	**3.63**		
CG31272	FBgn0051272	**3.62**		
Hsp68	FBgn0001230	**3.60**		
loh	FBgn0032252	**3.59**	**2.15**	
Tl	FBgn0262473	**3.58**	**2.30**	
sdk	FBgn0021764	**3.58**		
CG4301	FBgn0030747	**3.58**		
aret	FBgn0000114	**3.58**		
rdgB	FBgn0003218	**3.58**		
bgm	FBgn0027348	**3.58**	**4.62**	
vir-1	FBgn0043841	**3.56**	**8.76**	
CG43658	FBgn0263706	**3.53**		
LysX	FBgn0004431	**3.51**	**33.21**	**0.344**
eIF4E-6	FBgn0039622	**3.50**		
fend	FBgn0030090	**3.47**	**3.52**	
CG3679	FBgn0027521	**3.43**	**4.21**	
wrapper	FBgn0025878	**3.42**		
Glut1	FBgn0264574	**3.36**	**0.312**	**0.395**
CG34232	FBgn0085261	**3.35**		
CG12926	FBgn0033437	**3.34**	**0.415**	
HDC20112		**3.32**		
HMS-Beagle	FBte0000726	**3.32**		
Pepck	FBgn0003067	**3.29**		**2.30**
Mtk	FBgn0014865	**3.29**		**0.438**
Listericin	FBgn0033593	**3.28**		
Ugt35a	FBgn0026315	**3.27**	**3.35**	**2.53**
Ndae1	FBgn0259111	**3.26**		**2.10**
rdgB	FBgn0003218	**3.26**		
CG4269	FBgn0034741	**3.25**		
sda	FBgn0015541	**3.24**		
CG17207	FBgn0038051	**3.24**		
mthl14	FBgn0052476	**3.23**	**15.70**	
Spn31A	FBgn0032178	**3.22**		
CG5096	FBgn0032235	**3.19**	**3.96**	
nerfin-2	FBgn0041105	**3.18**		
CG10405	FBgn0038431	**3.16**		
hiw	FBgn0030600	**3.15**		
Arc1	FBgn0033926	**3.15**		
CG6357	FBgn0033875	**3.14**	**10.96**	
Nep2	FBgn0027570	**3.13**		**0.283**
d	FBgn0262029	**3.13**		
AttD	FBgn0038530	**3.13**	**72.76**	
Fie	FBgn0026592	**3.12**	**2.46**	
CG40040	FBgn0058040	**3.10**		
l(3)neo38	FBgn0265276	**3.10**		
CG32625	FBgn0052625	**3.09**	**39.17**	
CG9360	FBgn0030332	**3.06**	**10.35**	
CG3568	FBgn0029710	**3.03**		
CG31626	FBgn0051626	**3.00**	**19.15**	
CG32313	FBgn0052313	**3.00**		
spir	FBgn0003475	**3.00**	**2.15**	

tyn	FBgn0029128	**3.00**		
hdm	FBgn0029977	**2.98**		
CG42822	FBgn0262004	**2.98**		
CG32944	FBgn0052944	**2.97**		
CG33346	FBgn0053346	**2.97**		
alpha-Est3	FBgn0015571	**2.97**		
CG5191	FBgn0038803	**2.97**	**0.403**	
CG4297	FBgn0031258	**2.97**	**0.429**	
dys	FBgn0039411	**2.96**		
Ef1alpha100E	FBgn0000557	**2.95**		
QC	FBgn0052412	**2.95**		
stv	FBgn0086708	**2.94**		**2.53**
ZnT77C	FBgn0037000	**2.92**		
CG8738	FBgn0033321	**2.91**		
CG3251	FBgn0031622	**2.90**		
d	FBgn0262029	**2.90**		
Idgf2	FBgn0020415	**2.90**	**2.56**	
CG33494	FBgn0053494	**2.89**		
CG16749	FBgn0037678	**2.88**		
HDC20377		**2.86**		
CG33144	FBgn0053144	**2.86**		
Mdr49	FBgn0004512	**2.86**	**3.38**	
Cyp6a8	FBgn0013772	**2.83**		
Cyp6a2	FBgn0000473	**2.80**		**30.5**
RapGAP1	FBgn0264895	**2.80**	**0.339**	
CG6495	FBgn0027550	**2.80**		
CG32982	FBgn0052982	**2.79**	**2.33**	
Cyp4p1	FBgn0015037	**2.79**		
GstD5	FBgn0010041	**2.78**	**20.38**	
Het-A		**2.75**		
Kaz1-ORFB	FBgn0063923	**2.75**	**4.27**	
CG30456	FBgn0050456	**2.74**		
wisp	FBgn0260780	**2.74**		
CG8349	FBgn0032003	**2.73**		
CHKov2	FBgn0039328	**2.72**		
CG8046	FBgn0033388	**2.71**		
R1		**2.71**		
Rbf	FBgn0015799	**2.71**		
CG4741	FBgn0035040	**2.70**		
CG2217	FBgn0027544	**2.69**	**0.431**	
Ugt86Da	FBgn0040259	**2.68**	**8.97**	**2.28**
btn	FBgn0014949	**2.68**		
Jhl-26	FBgn0028424	**2.68**	**7.00**	**3.84**
AOX1	FBgn0267408	**2.67**	**14.75**	**2.60**
stai	FBgn0266521	**2.67**		
Ir41a	FBgn0040849	**2.66**		
Ugt86Dd	FBgn0040256	**2.65**	**4.15**	
PKD	FBgn0038603	**2.65**		**2.04**
Thor	FBgn0261560	**2.63**		
CG4398	FBgn0034126	**2.62**	**0.052**	
CG14692	FBgn0037836	**2.61**		
CG16712	FBgn0031561	**2.60**	**7.20**	**2.48**

Arc1	FBgn0033926	**2.59**	**5.93**	
Rhp	FBgn0026374	**2.57**	**0.328**	
COX7AL	FBgn0037579	**2.57**		
CG8066	FBgn0038243	**2.56**	**5.94**	
Muc14A	FBgn0052580	**2.56**		
CG15611	FBgn0034194	**2.56**		
Gs2	FBgn0001145	**2.55**	**3.09**	
CG33784	FBgn0053784	**2.55**		
KFase	FBgn0031821	**2.53**		
Gnmt	FBgn0038074	**2.53**	**8.13**	
CG17549	FBgn0032774	**2.52**		
pirk	FBgn0034647	**2.51**		
Doc2	FBgn0035956	**2.50**		
Hsp67Bc	FBgn0001229	**2.49**	**2.74**	**0.200**
egr	FBgn0033483	**2.49**		
CG7191	FBgn0031945	**2.48**		
CG3397	FBgn0037975	**2.48**		**0.162**
GstE7	FBgn0063493	**2.48**		**8.94**
GstE10	FBgn0063499	**2.48**		
CG11897	FBgn0039644	**2.48**	**5.52**	**2.14**
ome	FBgn0259175	**2.47**		
CG30022	FBgn0050022	**2.47**		
CG8008	FBgn0033387	**2.46**		
CG4822	FBgn0031220	**2.45**	**0.410**	
CG5783	FBgn0032670	**2.45**	**0.388**	
spri	FBgn0085443	**2.45**		
Traf4	FBgn0026319	**2.44**		
CG4829	FBgn0030796	**2.43**		
be	FBgn0052594	**2.42**		
CG8870	FBgn0038144	**2.41**		
Ptth	FBgn0013323	**2.39**		
KP78b	FBgn0026063	**2.38**	**6.25**	
CG14692	FBgn0037836	**2.38**		
Ror	FBgn0010407	**2.38**		
CG14642	FBgn0037222	**2.37**	**3.23**	
pdm3	FBgn0261588	**2.37**		
CG17029	FBgn0036551	**2.37**		
CG1358	FBgn0033196	**2.36**		
CG13813	FBgn0036956	**2.36**		
egr	FBgn0033483	**2.36**		
Inx3	FBgn0265274	**2.35**		
HDC20468		**2.35**	**8.59**	
CG6287	FBgn0032350	**2.35**		**0.382**
CG43064	FBgn0262366	**2.34**		
Pask	FBgn0034950	**2.32**		
DptB	FBgn0034407	**2.32**		
CG32521	FBgn0052521	**2.32**		
mtt	FBgn0050361	**2.32**		
Mef2	FBgn0011656	**2.32**		
CG30022	FBgn0050022	**2.31**		
CG18547	FBgn0037973	**2.30**	**4.73**	
Mmp1	FBgn0035049	**2.30**	**15.55**	**0.264**

GstD2	FBgn0010038	**2.30**	**9.61**	**0.235**
Nha2	FBgn0263390	**2.29**	**2.28**	**6.11**
IP3K2	FBgn0266375	**2.29**		
CG13116	FBgn0032139	**2.29**		**0.454**
Sip1	FBgn0010620	**2.29**	**0.396**	
CG4502	FBgn0031896	**2.28**		
Ect4	FBgn0262579	**2.28**		**2.06**
yellow-f	FBgn0041710	**2.27**	**0.330**	**0.358**
CG31743	FBgn0032618	**2.27**		**2.60**
tilB	FBgn0014395	**2.27**		
CG13893	FBgn0035146	**2.27**		
l(2)03659	FBgn0010549	**2.26**	**3.15**	
CR32207	FBgn0052207	**2.26**	**12.38**	
GstE2	FBgn0063498	**2.25**		
CG7255	FBgn0036493	**2.24**	**0.448**	
CG31253	FBgn0051253	**2.24**		
eater	FBgn0243514	**2.24**	**8.00**	
HDC20224		**2.23**	**5.63**	
CG7203	FBgn0031942	**2.23**		
CG13912	FBgn0035186	**2.23**		**2.27**
CG42240	FBgn0250869	**2.23**	**0.403**	
CG13698	FBgn0036773	**2.23**		
CG11686	FBgn0040551	**2.22**		
CG44325	FBgn0265413	**2.21**		
rst	FBgn0003285	**2.21**	**13.17**	
GstT4	FBgn0030484	**2.21**	**2.14**	**2.11**
CG7627	FBgn0032026	**2.21**		
IP3K2	FBgn0266375	**2.21**		
CG30485	FBgn0050485	**2.20**		
CG31038	FBgn0051038	**2.20**		
regucalcin	FBgn0030362	**2.20**		
CG14857	FBgn0038262	**2.20**		
GlyP	FBgn0004507	**2.19**		
Lsd-1	FBgn0039114	**2.19**		
CG3168	FBgn0029896	**2.19**		
Dyrk2	FBgn0016930	**2.18**		**0.448**
Tsp42Ea	FBgn0029508	**2.17**		
CG18063	FBgn0028856	**2.17**		
dnr1	FBgn0260866	**2.17**		
Rab23	FBgn0037364	**2.16**	**3.31**	
CG4404	FBgn0030432	**2.16**	**2.28**	
CG14291	FBgn0038660	**2.16**		
bbg	FBgn0087007	**2.15**	**0.392**	
Hsp23	FBgn0001224	**2.15**		
clos	FBgn0261016	**2.15**		
CG32368	FBgn0052368	**2.13**		
CG43164	FBgn0262720	**2.12**		
CG13654	FBgn0039290	**2.12**	**2.49**	
CG12428	FBgn0039543	**2.12**		
sick	FBgn0263873	**2.11**		
CG31436	FBgn0051436	**2.11**		
CG44251	FBgn0265186	**2.11**	**2.21**	

Inx2	FBgn0027108	**2.11**		
CG2065	FBgn0033204	**2.11**	**9.00**	**0.163**
CDase	FBgn0039774	**2.10**	**2.07**	
PGRP-SB1	FBgn0043578	**2.10**		**0.108**
CG12825	FBgn0033221	**2.10**	**2.64**	
brp	FBgn0259246	**2.09**		
CG13650	FBgn0039277	**2.08**		
MESK2	FBgn0043070	**2.08**		
CG31274	FBgn0051274	**2.08**	**0.326**	**0.418**
CG10283	FBgn0032681	**2.08**		
ebd2	FBgn0037076	**2.08**		
Ela	FBgn0013949	**2.08**		
pyd	FBgn0262614	**2.06**		
CG6231	FBgn0038720	**2.06**		
CecB	FBgn0000278	**2.06**		
CG14636	FBgn0037217	**2.05**	**0.355**	
veil	FBgn0034225	**2.05**		
CG43340	FBgn0263077	**2.05**		**2.22**
CG30460	FBgn0050460	**2.05**		
Cyp6d5	FBgn0038194	**2.05**		**0.490**
CG31705	FBgn0028490	**2.05**		
PGRP-LA	FBgn0035975	**2.05**		
CG6701	FBgn0033889	**2.05**		
GstD9	FBgn0038020	**2.04**		
pdgy	FBgn0027601	**2.03**		
spri	FBgn0085443	**2.03**		
mlt	FBgn0265512	**2.03**	**4.24**	
GstE6	FBgn0063494	**2.03**	**43.50**	**4.17**
CG32436	FBgn0052436	**2.03**		
Cyp4e2	FBgn0014469	**2.03**		
Gel	FBgn0010225	**2.02**		
Sans	FBgn0033785	**2.02**		
Tep4	FBgn0041180	**2.02**	**3.86**	
fat-spondin	FBgn0026721	**2.01**		
HDC03722		**2.00**		
HP1D3csd	FBgn0030994	**2.00**		
su(r)	FBgn0086450	**2.00**	**3.20**	**2.17**
Ih	FBgn0263397	**2.00**	**2.34**	
**HP1 depletion effect: *DOWN***				
CG32850	FBgn0052850	**0.498**	**4.43**	
CG41128	FBgn0069923	**0.490**	**0.338**	
CG3262	FBgn0032986	**0.486**		
Dbp80	FBgn0024804	**0.480**		
CG32350	FBgn0052350	**0.480**		
Acf1	FBgn0027620	**0.480**		
Fas2	FBgn0000635	**0.479**		**2.11**
ND-AGGG	FBgn0058002	**0.477**		
PlexB	FBgn0025740	**0.474**		
COX4L	FBgn0033020	**0.464**		
PlexB	FBgn0025740	**0.455**		
CG34330	FBgn0085359	**0.452**	**4.33**	
CG6329	FBgn0033872	**0.435**		

Nipped-A	FBgn0053554	**0.430**		
CG2709	FBgn0024977	**0.425**		
l(2)41Ab	FBgn0262123	**0.417**	**4.24**	
dob	FBgn0030607	**0.402**	**3.62**	
TpnC41C	FBgn0013348	**0.398**		
CG3829	FBgn0035091	**0.394**		
HDC20114		**0.370**		
Ser	FBgn0004197	**0.366**	**0.385**	
Transpac	FBte0001377	**0.363**		
CG41099	FBgn0039955	**0.362**		
CG3788	FBgn0034800	**0.353**		
CG17698	FBgn0040056	**0.347**		
br	FBgn0000210	**0.327**		
slgA	FBgn0003423	**0.316**	**0.345**	
mos	FBgn0033773	**0.309**		
Caps	FBgn0053653	**0.284**		
CR43242	FBgn0262887	**0.252**		**0.097**
CG40160	FBgn0058160	**0.239**	**0.441**	
CR43242	FBgn0262887	**0.239**		**0.097**
CG40006	FBgn0058006	**0.230**	**28.55**	
CG40294	FBgn0058294	**0.157**		**0.398**
CG30440	FBgn0050440	**0.132**		
Maf1	FBgn0267861	**0.129**		
Caps	FBgn0053653	**0.116**		
CR45923	FBgn0267585	**0.093**	**6.95**	
CG17698	FBgn0040056	**0.093**		
CG32017	FBgn0052017	**0.058**	**5.13**	
Cht3	FBgn0250907	**0.029**		
Su(var)205	FBgn0003607	**0.025**		

Transcripts that are up- or down-regulated by RNAi-mediated depletion of HP1 in Kc cells and H1 in L3 salivary glands or a homozygous null mutation of *Chd1* in L3 larvae. Fold change is calculated from Affymetrix microarray data relative to wild-type control. Transposons, repetitive and presumed heterochromatic sequences (Chr Xh, Chr 2h, Chr 3h and Chr4) are shown in red type.
