# Supplementary material for: A Genetic Screen and Transcript Profiling Reveal a Shared Regulatory Program for Drosophila Linker Histone H1 and Chromatin Remodeler CHD1
Source: G3 (Bethesda). 2015 Jan 27;5(4):677–87. doi: 10.1534/g3.115.016709 (PMC4390582; doi:10.1534/g3.115.016709)
Supplement: Supporting Information [file supp_g3.115.016709_TableS4.pdf]

Table S4 Overlap of transcripts regulated by ISWI, H1 and CHD1.

SYMBOL/ TRANSCRIPT	FLYBASE ID	FOLD CHANGE		
		ISWI KD	H1 KD	*Chd1*
**ISWI depletion effect:** ***UP***				
CG42675	FBgn0261561	**48.78**	**0.376**	
Ance-5	FBgn0035076	**37.27**	**2.73**	
CG33225	FBgn0053225	**27.57**	**31.76**	
Acer	FBgn0016122	**24.68**	**3.27**	
CG14787	FBgn0027793	**17.33**		**2.91**
CG33465	FBgn0053465	**16.53**	**2.80**	
CG42807	FBgn0261989	**16.34**		
CG9008	FBgn0028540	**15.47**		
Cyp6d5	FBgn0038194	**13.46**		**0.490**
CG45057	FBgn0266417	**13.26**	**2.20**	
GstD7	FBgn0010043	**12.65**	**3.98**	**3.61**
CG8399	FBgn0034067	**12.34**	**5.09**	
CG30091	FBgn0050091	**11.62**		**0.187**
yellow-f2	FBgn0038105	**11.48**	**5.78**	
nvy	FBgn0005636	**10.70**		
Obp44a	FBgn0033268	**10.65**	**32.99**	
zye	FBgn0036985	**10.55**		
ClC-a	FBgn0051116	**10.16**		
CG6357	FBgn0033875	**9.69**	**10.96**	
pgant2	FBgn0031530	**9.31**		
CG12910	FBgn0033502	**9.24**		
CG15202	FBgn0030271	**9.00**	**0.412**	
CG5397	FBgn0031327	**8.87**	**3.33**	
CG13077	FBgn0032810	**8.67**		
Cht2	FBgn0022702	**8.62**		
NimB3	FBgn0054003	**8.52**		
CG31219	FBgn0051219	**8.14**		
CG17321	FBgn0032719	**7.96**		
CG5731	FBgn0032192	**7.79**	**2.72**	
CG33120	FBgn0053120	**7.62**		
wdp	FBgn0034718	**7.45**		
Ggamma30A	FBgn0267252	**7.22**	**3.07**	
CG32483	FBgn0052483	**7.18**		**2.77**
Sodh-2	FBgn0022359	**7.12**		
CG17681	FBgn0032668	**7.00**		
CG10126	FBgn0038088	**6.97**		
MESK4	FBgn0043069	**6.97**	**0.342**	**0.473**
Obp49a	FBgn0050052	**6.88**		
CG10311	FBgn0038420	**6.87**		
fng	FBgn0011591	**6.74**		
Gli	FBgn0001987	**6.60**		
CG16710	FBgn0039101	**6.33**		
CG14990	FBgn0035496	**6.14**		
Mtk	FBgn0014865	**6.09**		**0.438**
mthl14	FBgn0052476	**6.02**	**15.70**	
CG11638	FBgn0040351	**5.97**		

CG30345	FBgn0050345	**5.74**		
CG5001	FBgn0031322	**5.55**		
e	FBgn0000527	**5.46**	**2.93**	**0.042**
CG42369	FBgn0259715	**5.45**	**7.66**	
Mdr49	FBgn0004512	**5.34**	**3.38**	
RpS30	FBgn0038834	**5.33**		
CG1124	FBgn0037290	**5.25**		**0.493**
CG31274	FBgn0051274	**5.24**	**0.326**	**0.418**
Kal1	FBgn0039155	**5.17**	**0.168**	
dmGlut	FBgn0010497	**5.17**	**0.167**	
CG17029	FBgn0036551	**5.17**		
Fuca	FBgn0036169	**5.14**	**3.88**	
Tsp42Ea	FBgn0029508	**5.03**		
qsm	FBgn0028622	**4.98**		
lea	FBgn0002543	**4.97**	**3.29**	
yellow-c	FBgn0041713	**4.92**		**0.146**
cad	FBgn0000251	**4.91**	**8.04**	
CG6830	FBgn0037934	**4.83**		
dpr17	FBgn0051361	**4.81**		
CG13377	FBgn0261446	**4.65**		
CG11378	FBgn0040364	**4.62**	**0.331**	
CG9691	FBgn0030160	**4.55**		
ChLD3	FBgn0032598	**4.44**		
CG17751	FBgn0038717	**4.43**		
CG13921	FBgn0035267	**4.27**	**4.49**	
twz	FBgn0034636	**4.25**		**0.232**
CG30148	FBgn0050148	**4.22**		
CG7966	FBgn0038115	**4.19**		
CG31743	FBgn0032618	**4.18**		**2.60**
Snap25	FBgn0011288	**4.17**	**33.90**	
CG9801	FBgn0037623	**4.13**	**0.360**	**0.423**
CG14516	FBgn0039640	**4.05**		
Gs1	FBgn0001142	**3.89**		
CG10359	FBgn0035452	**3.89**		
Ude	FBgn0039226	**3.85**		**5.03**
Drs	FBgn0010381	**3.78**	**4.28**	**0.451**
CG7607	FBgn0036145	**3.71**	**3.73**	
CG15772	FBgn0029799	**3.70**		
sPLA2	FBgn0033170	**3.67**		
CG32313	FBgn0052313	**3.63**		
twit	FBgn0032895	**3.59**		
PCB	FBgn0027580	**3.58**		
Gyc-89Db	FBgn0038436	**3.56**		
Cyp6d2	FBgn0034756	**3.53**		
CG16789	FBgn0037712	**3.47**		
Gk	FBgn0035266	**3.39**		
Cby	FBgn0067317	**3.38**		
CG7860	FBgn0030653	**3.36**		
Mmp1	FBgn0035049	**3.34**	**11.63**	**0.264**
yellow-b	FBgn0032601	**3.34**		
Pka-C3	FBgn0000489	**3.28**		
CG7781	FBgn0032021	**3.26**		

Cyp6a17	FBgn0015714	**3.22**	**3.26**	
CG45263	FBgn0266801	**3.22**		
GlcAT-S	FBgn0032135	**3.20**	**0.331**	
CG10877	FBgn0038804	**3.18**	**0.157**	
btn	FBgn0014949	**3.16**		
PlexB	FBgn0025740	**3.14**		
CG3505	FBgn0038250	**3.10**		
CG30380	FBgn0050380	**3.09**		
SkpB	FBgn0026176	**3.08**		
GLaz	FBgn0033799	**3.07**	**4.64**	
Cyp6a23	FBgn0033978	**3.07**	**16.25**	**5.98**
Dhc36C	FBgn0013810	**3.07**		
CG15343	FBgn0030029	**3.07**	**0.124**	**0.423**
ft	FBgn0001075	**3.06**		
MESK2	FBgn0043070	**3.04**		
CG6218	FBgn0038321	**3.01**		
Ama	FBgn0000071	**3.01**	**2.27**	**0.371**
Oat	FBgn0022774	**3.00**	**0.077**	**0.306**
fax	FBgn0014163	**2.98**		
CG34288	FBgn0085317	**2.96**		
baz	FBgn0000163	**2.96**	**0.369**	
CG4630	FBgn0033809	**2.95**	**0.301**	
CG5783	FBgn0032670	**2.95**	**0.388**	
LamC	FBgn0010397	**2.95**		**2.08**
CG3625	FBgn0031245	**2.92**		
Cyp6a22	FBgn0013773	**2.92**	**7.50**	
PPO1	FBgn0261362	**2.92**	**6.89**	
CG12262	FBgn0035811	**2.91**		
miple2	FBgn0029002	**2.87**		
LpR1	FBgn0066101	**2.87**		
Jheh1	FBgn0010053	**2.85**		
slgA	FBgn0003423	**2.85**	**0.345**	
Pepck	FBgn0003067	**2.84**		**2.30**
Cyp4d20	FBgn0035344	**2.81**		
CG5577	FBgn0036759	**2.81**	**0.359**	
CG34409	FBgn0085438	**2.80**		
brv3	FBgn0040333	**2.80**		
AdamTS-A	FBgn0038341	**2.79**	**0.207**	
rgn	FBgn0261258	**2.78**	**11.07**	
Dat	FBgn0019643	**2.74**		
CG8547	FBgn0033919	**2.72**		
Gbp	FBgn0034199	**2.72**		**0.429**
CG10232	FBgn0039108	**2.68**	**4.13**	
CG14036	FBgn0031677	**2.68**		
HDC15448		**2.66**		
corolla	FBgn0267967	**2.66**		
Ror	FBgn0010407	**2.66**		
pdgy	FBgn0027601	**2.66**		
fra	FBgn0011592	**2.65**		
CG1969	FBgn0039690	**2.65**	**0.218**	
S.C3R003414		**2.64**		
wus	FBgn0030805	**2.62**		

CG8353	FBgn0032002	**2.62**		
CG10764	FBgn0034221	**2.61**		**0.390**
CG30503	FBgn0050503	**2.60**		**3.14**
CG11983	FBgn0037654	**2.60**		
CG11897	FBgn0039644	**2.59**	**5.52**	**2.14**
Mmp1	FBgn0035049	**2.57**	**9.53**	**0.264**
Cyp310a1	FBgn0032693	**2.57**	**6.31**	
Cyp4d2	FBgn0011576	**2.57**		
whd	FBgn0261862	**2.56**		
CG8066	FBgn0038243	**2.55**	**5.94**	
betaTub60D	FBgn0003888	**2.55**	**3.13**	
CG32436	FBgn0052436	**2.53**		
Cyp6a20	FBgn0033980	**2.52**		
Cyp12a4	FBgn0038681	**2.52**	**3.42**	
Src64B	FBgn0262733	**2.50**		
c(2)M	FBgn0028525	**2.50**	**2.93**	
Npc2b	FBgn0038198	**2.49**		
CG42390	FBgn0259736	**2.48**	**0.464**	**2.51**
mbc	FBgn0015513	**2.47**		
CG31751	FBgn0086909	**2.47**	**0.375**	
Adk3	FBgn0042094	**2.47**		
Spn27A	FBgn0028990	**2.47**		
dpp	FBgn0000490	**2.46**		
Mmp1	FBgn0035049	**2.46**	**15.55**	**0.264**
CG17549	FBgn0032774	**2.44**		
CG34331	FBgn0085360	**2.44**		
Hydr1	FBgn0033382	**2.43**		
CG32280	FBgn0052280	**2.43**		
Tequila	FBgn0023479	**2.43**		
Atpalpha	FBgn0002921	**2.42**		
nrv3	FBgn0032946	**2.42**	**8.16**	
Hsp27	FBgn0001226	**2.41**		**0.457**
corn	FBgn0259173	**2.41**	**12.44**	
CG17350	FBgn0032772	**2.40**		
CG15544	FBgn0039804	**2.40**		**0.406**
CG8501	FBgn0033724	**2.40**		
CG12206	FBgn0029662	**2.40**		
GlyP	FBgn0004507	**2.39**		
Tsp42Ed	FBgn0029507	**2.39**		
NtR	FBgn0029147	**2.39**	**2.51**	
CG7142	FBgn0038595	**2.38**	**5.56**	
CG15093	FBgn0034390	**2.37**	**2.02**	
CDase	FBgn0039774	**2.36**	**2.07**	
sano	FBgn0034408	**2.35**	**4.83**	
EF-G2	FBgn0051159	**2.35**		
Dmtn	FBgn0037443	**2.34**		
CG1969	FBgn0039690	**2.34**	**0.189**	
CG13907	FBgn0035173	**2.33**		
sdk	FBgn0021764	**2.33**		
CG40115	FBgn0058115	**2.33**	**10.26**	
CG10824	FBgn0038865	**2.30**	**2.24**	
CG14984	FBgn0035480	**2.30**	**2.07**	

Jabba	FBgn0259682	**2.30**		
Cyp6a13	FBgn0033304	**2.30**	**5.64**	
CG17919	FBgn0037433	**2.29**	**9.35**	
Ance	FBgn0012037	**2.28**		
CG18508	FBgn0028746	**2.28**		
CG33111	FBgn0053111	**2.28**		
Itgbetanu	FBgn0010395	**2.27**		
CG43658	FBgn0263706	**2.27**		
CG16947	FBgn0031816	**2.27**	**0.285**	
Cyp12d1-d	FBgn0053503	**2.26**		
CG33181	FBgn0053181	**2.26**	**7.63**	
CG13252	FBgn0037016	**2.25**	**0.440**	
Asph	FBgn0034075	**2.25**	**0.427**	**0.308**
dnd	FBgn0038916	**2.25**		
Mctp	FBgn0034389	**2.25**	**0.300**	**2.95**
CG15914	FBgn0030700	**2.24**		
dlp	FBgn0041604	**2.24**		
bgcn	FBgn0004581	**2.23**		
Apoltp	FBgn0032136	**2.22**	**2.89**	**5.70**
CG40115	FBgn0058115	**2.22**	**7.20**	
CG3831	FBgn0034804	**2.22**		**0.480**
CG5853	FBgn0032167	**2.20**		
CG12746	FBgn0037341	**2.20**		
springer	FBte0000333	**2.19**		
SD02481	FBcl0277517	**2.18**		
fat-spondin	FBgn0026721	**2.18**		
Fem-1	FBgn0034542	**2.17**		
Oatp30B	FBgn0032123	**2.17**		
mthl5	FBgn0037960	**2.17**		**0.476**
SAK	FBgn0026371	**2.16**		
Vha68-1	FBgn0265262	**2.15**	**2.42**	
norpA	FBgn0262738	**2.15**		
Ect3	FBgn0260746	**2.15**	**2.21**	
CG3038	FBgn0040373	**2.14**		
CG32032	FBgn0043806	**2.14**		
CG9547	FBgn0031824	**2.13**		
CG12643	FBgn0040942	**2.12**		
CG31974	FBgn0051974	**2.12**	**4.74**	**2.23**
CAH1	FBgn0027844	**2.11**	**2.25**	**0.493**
mtg	FBgn0260386	**2.11**	**2.25**	**2.83**
CG30441	FBgn0050441	**2.11**		
TM4SF	FBgn0020372	**2.11**		
Cpr49Ag	FBgn0033730	**2.10**		
ImpE3	FBgn0001255	**2.10**		**0.171**
CG10516	FBgn0036549	**2.10**		**0.293**
zuc	FBgn0261266	**2.09**		
Tret1-1	FBgn0050035	**2.07**		
Muc68Ca	FBgn0036181	**2.07**		**7.36**
CG17754	FBgn0030114	**2.07**		**0.490**
CG45186	FBgn0266696	**2.06**	**0.433**	
Atet	FBgn0020762	**2.06**		**0.470**
CG5080	FBgn0031313	**2.06**		

cyc	FBgn0023094	**2.05**		
CG33947	FBgn0083068	**2.05**		
SLC5A11	FBgn0031998	**2.05**		
CG14523	FBgn0039612	**2.04**		
CG42684	FBgn0261570	**2.04**		
CG17265	FBgn0031488	**2.03**		
CG42327	FBgn0259227	**2.03**		
CG5096	FBgn0032235	**2.03**	**3.96**	
CG9914	FBgn0030737	**2.03**	**4.84**	
Dip3	FBgn0040465	**2.03**		
mthl10	FBgn0035132	**2.03**		
Dark	FBgn0263864	**2.02**	**0.249**	**0.418**
Apf	FBgn0051713	**2.02**		
CG6330	FBgn0039464	**2.01**		
ItgalphaPS4	FBgn0034005	**2.01**	**3.91**	
FER	FBgn0000723	**2.01**		
CG41128	FBgn0069923	**2.00**	**0.338**	
**ISWI depletion effect: *DOWN***				
CG40191	FBgn0058191	**0.498**		
laccase2	FBgn0259247	**0.498**		
CG7322	FBgn0030968	**0.495**		
ppk29	FBgn0034965	**0.495**		
CG31619	FBgn0051619	**0.495**	**2.82**	
CG12391	FBgn0033581	**0.494**		
Nap1	FBgn0015268	**0.494**		
Gpo-1	FBgn0022160	**0.493**	**3.37**	**3.32**
Doc3	FBgn0035954	**0.490**		
HDC14725		**0.488**		
CG10433	FBgn0034638	**0.487**	**3.25**	**3.05**
CG8765	FBgn0036900	**0.487**		
HDC16707		**0.485**		
cactin	FBgn0031114	**0.484**	**2.77**	
Marcal1	FBgn0031655	**0.484**		
lute	FBgn0262871	**0.484**	**0.286**	
CG32625	FBgn0052625	**0.482**	**39.17**	
ham	FBgn0045852	**0.480**		
CG7900	FBgn0037548	**0.476**	**2.96**	**2.71**
CG7120	FBgn0035888	**0.475**		
Gs2	FBgn0001145	**0.474**	**3.09**	
Cdep	FBgn0265082	**0.472**	**0.481**	
mgl	FBgn0261260	**0.471**		
Arpc3B	FBgn0065032	**0.470**	**30.56**	
Sp7	FBgn0037515	**0.466**		**0.401**
CG31997	FBgn0051997	**0.465**	**3.22**	
Aats-val	FBgn0027079	**0.465**		
Eip55E	FBgn0000566	**0.463**		
Aats-asn	FBgn0086443	**0.456**		
CG40131	FBgn0058131	**0.454**		
CG34228	FBgn0085257	**0.454**		
CG18747	FBgn0042104	**0.453**		
blanks	FBgn0035608	**0.453**	**98.58**	**0.321**
CG44251	FBgn0265186	**0.450**	**2.21**	

PGRP-SA	FBgn0030310	**0.449**	**2.20**	
squ	FBgn0267347	**0.448**		
CG12288	FBgn0032620	**0.447**	**5.29**	
CG42724	FBgn0261641	**0.443**		
Taf2	FBgn0011836	**0.443**		
Nop60B	FBgn0259937	**0.443**	**3.70**	
gce	FBgn0261703	**0.443**		
CG1503	FBgn0031157	**0.443**		
CG10089	FBgn0036369	**0.436**	**13.85**	
Ssk	FBgn0036945	**0.431**		
Ac3	FBgn0023416	**0.430**		
r-l	FBgn0003257	**0.430**		
CG32088	FBgn0052088	**0.430**		
CG14798	FBgn0029588	**0.428**	**3.41**	
sgll	FBgn0051472	**0.424**		
CG32795	FBgn0040384	**0.422**	**0.468**	
png	FBgn0000826	**0.420**		
CG1550	FBgn0033225	**0.419**		
CG30339	FBgn0050339	**0.417**		
CG18643	FBgn0037898	**0.415**		
CG7458	FBgn0037144	**0.413**		**2.06**
CG2709	FBgn0024977	**0.411**		
Aats-trp	FBgn0010803	**0.411**	**0.171**	
CG9902	FBgn0030757	**0.411**		
CG6912	FBgn0038290	**0.408**		
Gip	FBgn0011770	**0.407**		
CG30340	FBgn0050340	**0.406**		
sage	FBgn0037672	**0.404**	**0.183**	
HDC17231		**0.399**		
CG32017	FBgn0052017	**0.396**	**5.13**	
Cyt-b5-r	FBgn0000406	**0.393**	**10.17**	**2.57**
Mal-B2	FBgn0032382	**0.391**		
CG8613	FBgn0033924	**0.390**		
f-cup	FBgn0028487	**0.385**		
CG31769	FBgn0051769	**0.380**	**2.49**	**2.33**
trk	FBgn0003751	**0.380**	**4.61**	
CG32196	FBgn0052196	**0.378**		
CG1674	FBgn0039897	**0.378**		
GstO1	FBgn0035907	**0.377**	**6.65**	
CG4793	FBgn0028514	**0.374**	**15.37**	
alpha-Est8	FBgn0015576	**0.374**		**2.83**
Bx	FBgn0265598	**0.373**	**2.05**	
CG14545	FBgn0040602	**0.372**	**157.17**	
CG14615	FBgn0031184	**0.370**	**2.16**	
CG4587	FBgn0028863	**0.370**		
CG15100	FBgn0034401	**0.362**		
nonA-l	FBgn0015520	**0.353**		
CG30010	FBgn0050010	**0.352**		
CG18586	FBgn0035642	**0.347**		
CG1677	FBgn0029941	**0.346**		
CT32987		**0.346**		
CG9305	FBgn0032512	**0.342**		

CG10063	FBgn0035727	**0.335**		
CG5568	FBgn0035641	**0.334**		
fend	FBgn0030090	**0.324**	**3.52**	
CG15820	FBgn0035312	**0.322**		
Vago	FBgn0030262	**0.322**	**2.46**	
Sox102F	FBgn0039938	**0.321**		
CG9837	FBgn0037635	**0.320**		**0.441**
aub	FBgn0000146	**0.319**		
ham	FBgn0045852	**0.301**		
CG16888	FBgn0032533	**0.301**		
blot	FBgn0027660	**0.298**	**0.273**	
TfIIFalpha	FBgn0010282	**0.292**		
CG31619	FBgn0051619	**0.291**		
CG5285	FBgn0038490	**0.291**	**2.33**	
betaTub97EF	FBgn0003890	**0.284**		
CG17928	FBgn0032603	**0.280**	**2.79**	
stg1	FBgn0064123	**0.270**		
CG34330	FBgn0085359	**0.260**	**4.33**	
CG32582	FBgn0052582	**0.252**		
AGO3	FBgn0250816	**0.250**	**11.47**	
GstE13	FBgn0033381	**0.246**		
CG8336	FBgn0036020	**0.244**		
CG43103	FBgn0262563	**0.239**	**2.59**	
veil	FBgn0034225	**0.232**		
CG5144	FBgn0035957	**0.223**		
CG3457	FBgn0024984	**0.220**		
piwi	FBgn0004872	**0.209**		
CG10581	FBgn0037046	**0.206**	**5.98**	
CG4872	FBgn0030799	**0.183**	**10.25**	
Nxf3	FBgn0263232	**0.183**		
Ugt36Bc	FBgn0040260	**0.181**		
CG4210	FBgn0038302	**0.169**	**2.30**	
Nrg	FBgn0264975	**0.144**		
CG13602	FBgn0264740	**0.130**		
Iswi	FBgn0011604	**0.126**		
Nrg	FBgn0264975	**0.113**		
eIF4E-6	FBgn0039622	**0.112**		
fu12	FBgn0026718	**0.101**	**3.56**	
CG5367	FBgn0032228	**0.053**		

Transcripts that are up- or down-regulated by RNAi-mediated depletion of ISWI in SL2 cells and H1 in L3 salivary glands or a homozygous null mutation of *Chd1* in L3 larvae. Fold change is calculated from Affymetrix microarray data relative to wild-type control.
